# Adaptation Model for Patient and Caregiver Dyads in Hospital-to-Home Transition: Theory Development and Content Validation

**DOI:** 10.3390/healthcare14182905

**Published:** 2026-09-08

**Authors:** Gloria Carvajal-Carrascal, Alejandra Fuentes-Ramírez, Ricardo Sotaquirá-Gutiérrez, Mayerly Andrea Medina-Jutinico, Alejandra Rojas-Rivera, Beatriz Sánchez-Herrera

**Affiliations:** 1 School of Life Sciences and Well-Being, Universidad de La Sabana, Chía 250001, Colombia; gloria.carvajal@unisabana.edu.co (G.C.-C.); publifer@unisabana.edu.co (B.S.-H.); 2 School of Engineering, Universidad de La Sabana, Chía 250001, Colombia; ricardo.sotaquira@unisabana.edu.co; 3 Nursing Deputy Directorate, Clinica Universidad de La Sabana, Chía 250001, Colombia; mayerly.medina@clinicaunisabana.edu.co; 4 Nursing and Midwifery School, Universidad de los Andes, Santiago 12455, Chile; arojas@uandes.cl

**Keywords:** hospital-to-home transition, transitional care, patient discharge, caregivers, adaptation, psychological, nursing models, healthcare models

## Abstract

**Highlights:**

**What are the main findings?**

- This study introduces the *Adaptarte Model*, a middle-range nursing theory developed through a sequential multimethod design to provide a contextualized conceptual framework for dyadic adaptation during the hospital-to-home transition (H-HT).
- The model establishes high content validity and conceptual coherence, positioning the patient–family caregiver dyad as the primary unit of care and proposing testable theoretical propositions to establish a foundation for subsequent empirical testing in Latin America.

**What are the implications of the main findings?**

- The content-validated *Adaptarte Model* offers health institutions a structured theoretical blueprint to conceptualize and design contextualized, dyad-centered transitional care.
- The model provides the conceptual basis for the prospective development of specialized clinical protocols, training programs, and nursing quality indicators focused on transitional care.
- These findings establish a robust theoretical foundation for future empirical studies evaluating the model’s implementation, construct validity, and clinical effectiveness within health systems.

**Abstract:**

**Background/Objective**: The H-HT represents a critical vulnerability for patient–family caregiver dyads. This study developed and content-validated a middle-range nursing theory, the *Adaptarte Model*, designed to guide dyadic adaptation during the H-HT within the Latin American healthcare context. **Methods**: A sequential exploratory multimethod design was executed in two phases. Phase 1 integrated three evidence streams: clinical practice insights, a JBI-guided scoping review, and two focus groups with transitional care professionals. Qualitative content analysis and iterative consensus refined the model’s core concepts, assumptions, and propositions. Phase 2 evaluated the model’s content, structure, functionality, and projection using an international panel of eleven Latin American experts meeting strict eligibility criteria. Data were analyzed using Lawshe’s Content Validity Ratio (CVR) modified by Tristán (cutoff = 0.58) and the overall Content Validity Index (*CVI*). Reporting followed PRISMA-ScR and GRAMMS guidelines. **Results**: Expert consensus confirmed the essential model components. Item-level CVR values ranged from 0.90 to 0.99, yielding an overall *CVI* of 0.96, while external functionality and conceptual projection achieved an average rating of 0.88. **Conclusions**: The *Adaptarte Model* demonstrates high content validity and structural clarity, establishing a rigorous theoretical foundation for subsequent empirical research. Rather than being ready for immediate clinical implementation, it provides a structured blueprint for prospective protocol development. Systematic empirical testing and longitudinal studies are now imperative to evaluate its clinical utility and drive future healthcare transformations. The scoping review protocol was prospectively registered on the Open Science Framework (OSF) URL (accessed on 23 September 2024).

## 1. Introduction

The World Health Organization (WHO) has established a global commitment to enhancing health and safety during healthcare transitions [1]. These transitions encompass dynamic processes of change that demand adaptation, support, and professional guidance to ensure optimal clinical outcomes [2]. The H-HT is widely acknowledged as a pivotal phase during which patient–family caregiver dyads experience heightened vulnerability [3].

The H-HT is defined as a process that commences upon hospitalization and extends through the 30-day period following the patient’s return home, accompanied by a family or designated caregiver. This period encompasses significant shifts in health services, support structures, and clinical conditions experienced by the patient–family caregiver dyad [4]. Consequently, the H-HT impacts patient–family caregiver dyads, healthcare professionals, and the institutions responsible for their care [5]. Addressing this transition effectively requires meticulously planned and coordinated processes among individuals, healthcare institutions, and provider networks to ensure continuity of care [6]. For these reasons, the H-HT has become a primary area of interest within nursing, serving as a critical scenario for safeguarding dyadic safety and autonomy [7].

From the perspective of patient–family caregiver dyads, the H-HT is a period that demands coping and the strengthening of autonomy, both of which require adequate support [8]. However, dyads often perceive transitional services as impersonal, leading to the feeling that individual expectations are unmet, which results in stress and anxiety [9]. Healthcare recipients also report a sense of inconsistency, as well as a lack of opportunity and continuity in service delivery during this period [10]. These challenges highlight the need for a systematic intervention designed to assist the dyad and promote adaptation throughout this process.

From the perspective of healthcare professionals and institutions, the H-HT demands careful planning capable of anticipating the dyads’ changing needs to prevent complications and clinical deterioration. This planning encompasses staffing, comprehensive assessment, situation management, education, motivation, the active inclusion of individuals facing the H-HT, follow-up management, and the coordination of required services [11]. Currently, staff may fail to recognize the support and communication needs of these dyads [12]. Similarly, there is a clear requirement for harmonized guidelines and approaches to comprehensively evaluate the cost–benefit ratio of care during the H-HT [13].

At a societal level, literature reports considerable progress in care during hospital stays, responding to biomedical, care-based, and interactive paradigms [14]. Increasingly relevant networked approaches—as well as their integration to achieve better health experiences, improved clinical outcomes, and lower healthcare costs—demand the development of explicit guidelines for nursing practice in this field [15]. Evidence also indicates that health transition services are directly correlated with the guarantee of health equity [16].

Nursing-led interventions proposed to support this transition, especially those of low-to-medium complexity incorporating community-based follow-up, have successfully reduced readmissions and emergency room visits while enhancing quality of life [17,18]. These interventions highlight the crucial role of the family caregiver [19]. Programs that include multiple care components—such as home visits, the utilization of appropriate technologies, case management models, multidisciplinary teams, and support for self-management—are especially vital to guaranteeing access, resources, and adaptation within a specific context [20].

In Latin America, the Pan American Health Organization (PAHO) promotes policies to strengthen long-term care through integrated and continuous services that reduce the overall burden of care. These policies emphasize strengthening professional nursing competencies to achieve higher-quality care and developing theoretical and methodological guidelines to support implementation [21]. While multiple models exist to inform nursing practice during the H-HT, their implementation by nurses and healthcare teams within the Latin American context remains insufficient. Recent experiences reported in Brazil [22], Argentina [23], Chile [24], and Mexico [25] do not clearly reflect the nature and specific practice of nursing in supporting adaptation among patient–family caregiver dyads during the H-HT within this regional context.

Based on this gap between nursing theory and practice, our research group sought to develop a middle-range nursing theory (the *Adaptarte Model*) specifically designed to support adaptation within patient–family caregiver dyads during the H-HT.

The main objective of this study is to develop a middle-range nursing theory to support adaptation in patient–family caregiver dyads during the H-HT within the Latin American context. Two specific objectives directed the course of this research: first, to design a model informed by both experiential knowledge and the best available evidence to guide nursing practice during the H-HT; and second, to validate the content, functionality, and projection of the proposed model by engaging an international panel of Latin American experts in a comprehensive review process.

Ultimately, this study aims to provide an evidence-based framework that serves as a clear guide to orienting practice, systematizing processes, improving intra- and interdisciplinary communication, and promoting optimized care for patient–family caregiver dyads across the Latin American healthcare landscape.

## 2. Materials and Methods

The study employed a sequential exploratory multi-method design. This design was selected because it is particularly appropriate for the systematic development and validation of new theoretical frameworks when significant conceptual or contextual gaps persist in the application of existing models to a specific population or setting [26].

The study was conducted in three sequential phases. These phases enabled the development of a preliminary theoretical framework, the validation of its content, structure, functionality, and potential impact, and the integration of the findings into the final version of the *Adaptarte Model*. The resulting model provides a theoretical framework that requires subsequent empirical validation to evaluate its contribution to promoting adaptation within the patient–family caregiver dyad during the H-HT.

### 2.1. Phase 1, Model Design and Theoretical Derivation

Phase 1 focused on the conceptual design and theoretical construction of the *Adaptarte Model* through a sequential, iterative, and inductive–deductive approach. This phase was executed across three structured steps: (1) evidence foundation through a scoping review, (2) empirical refinement via qualitative inquiry with clinical key informants, and (3) conceptual abstraction, integration, and refinement of the initial model proposal.

Step 1: Evidence Foundation and Scoping Review

The evidence synthesis step was driven by a scoping review conducted in accordance with the JBI methodology [27], serving explicitly as the empirical groundwork for theoretical derivation. This approach enabled a systematic mapping of available literature on adaptation during the H-HT, identifying core concepts, theoretical perspectives, empirical findings, and knowledge gaps.

The search strategy combined Medical Subject Headings (MeSH) and free-text terms: [(“Hospital to Home Transition” OR “Transitional Care”) AND (“Adaptation”) AND (“Nursing” OR “Nursing Model” OR “Nursing Theory”) AND (“Patient” OR “Caregivers”)]. Electronic searches were conducted across PubMed, Embase, SciELO, CINAHL, and ScienceDirect, supplemented by Google Scholar to capture additional peer-reviewed literature. Records were imported into reference management software, deduplicated, and screened according to a prospective protocol registered on the OSF URL (accessed on 23 September 2024): https://osf.io/mf2ux/files/5znps.

Inclusion was restricted to peer-reviewed articles published between 2014 and March 2025, with no language or geographic restrictions. To maintain conceptual density and methodological consistency, non-peer-reviewed sources, grey literature, and studies without full-text access were excluded.

Title, abstract, and full-text screening were conducted independently by three reviewers, with discrepancies resolved through consensus with three senior researchers. Included studies underwent bibliometric mapping, critical appraisal, and evidence hierarchy classification using the LoBiondo-Wood scale [28]. Reporting adhered strictly to PRISMA-ScR guidelines [29].

To maintain methodological rigor during theoretical synthesis, the evidence was continuously weighted. Once the studies were reviewed, the researchers used an analysis matrix to classify each study’s conceptual, methodological, and operational contributions. Through this process, descriptive and qualitative studies enriched the understanding of essential concepts, core assumptions, and caregiving dynamics and contexts. Methodologically robust studies further strengthened this conceptual and contextual foundation while providing the empirical basis for expected clinical outcomes, central relational propositions, and hypotheses to be tested in future empirical research. Continuous iteration, alongside the research team’s clinical and methodological expertise, ensured the structural and operational coherence of the developing proposal. Finally, all constructs were cross-validated through triangulation with the qualitative focus group data from the empirical phase of this study.

*Step 2: Empirical Refinement via Qualitative Synthesis*

To ground the preliminary theoretical framework in real-world clinical practice, empirical refinement was conducted through two qualitative focus groups aimed at identifying core attributes of dyadic adaptation during H-HT. For the first group participants were purposively selected based on their recognized proficiency in transitional care and nursing practice. This group comprised nine clinical nurses with recognized expertise in transitional care (n=9), with a minimum of three years of professional experience in H-HT. The second group included four healthcare professionals from different disciplines with experience in transitional care management (n=4).

Focus groups were conducted in person, lasted two hours on average, and were audio-recorded and transcribed verbatim. Rather than pursuing theoretical saturation through iterative sampling, the focus groups were designed to complement and critically examine the concepts identified in the scoping review. Data collection was considered sufficient when discussions yielded no substantively new conceptual insights and consistently confirmed, refined, or contextualized the preliminary theoretical framework.

Qualitative content analysis was performed manually by three independent researchers who coded transcripts, identified meaning units, generated initial codes, and organized them into thematic categories, resolving discrepancies through team consensus. Methodological rigor and credibility were ensured through researcher triangulation, iterative team debriefings, cross-comparison between focus groups, and member checking, wherein synthesized findings were returned to participants for validation. The analysis identified critical contextual determinants that complemented the evidence derived from the scoping review, including perceived clinical needs, professional and institutional barriers, priority components of continuity of care, and structural characteristics specific to Latin American healthcare systems.

*Step 3: Integration, Conceptual Abstraction, and Model Optimization*

In the final step, qualitative findings were integrated with scoping review evidence through constant comparative analysis to identify conceptual gaps, shared elements, and structural attributes. This iterative process synthesized the findings into a unified logical framework encompassing the model’s central concepts: the primary subject of care (the patient–family caregiver dyad) and the nurse as a complementary subject of care within the adaptation process; the care environment (structural facilitators and systemic barriers), therapeutic nursing actions, the goal of care (multidimensional dyadic adaptation), and clinical-psychosocial outcomes.

Convergent findings reinforced core theoretical structures, whereas divergent elements were deliberated by the research team until conceptual consensus was reached. This analytical process yielded a second version of the framework, which was first evaluated by a multidisciplinary panel of nine health researchers and subsequently reviewed by four experts in nursing theory. Feedback from both groups informed the refinement of a preliminary version of the framework. This preliminary review confirmed the framework’s theoretical rigor, internal coherence, and potential clinical utility, while consolidating the core concepts, foundational assumptions, relational propositions, and testable hypotheses of the *Adaptarte Model* before its formal content validation by an expert panel.

### 2.2. Phase 2, Model Content Validation

To ensure structural rigor regarding content, functionality, and projection, the *Adaptarte Model* underwent formal content validation by an international panel of 11 Latin American experts (*n* = 11), selected via purposive sampling. In alignment with established psychometric guidelines in research a panel size of 11 experts is methodologically sound to secure content validity while controlling for agreement by chance [30]. Potential judges were identified through professional and academic networks specializing in healthcare transitions. Eligibility criteria required experts to hold a postgraduate degree, maintain active practice within an interdisciplinary healthcare team alongside nursing professionals, possess a documented track record of peer-reviewed publications, and have over three years of specialized experience in transitional care within the Latin American context.

For the evaluation process, each expert received a copy of the proposed model along with an evaluation tool to assess its content based on clarity, coherence, relevance, and sufficiency. Clarity measured whether each dimension utilized suitable syntax and semantics for clear comprehension. Coherence evaluated the logical relationship among the elements within each dimension. Relevance determined the significance of the content in supporting the proposed themes, while sufficiency assessed whether the content of a given dimension was adequate to support the overall model. To rate these criteria, a four-point Likert scale was used, ranging from 1 (does not meet the criterion) to 4 (completely meets the criterion). The research group also collected qualitative suggestions and comments to further refine the proposal. Subsequently, within the same tool, the experts evaluated the functionality and projection of the model. Functionality reflected the extent to which the model provides an effective framework for nursing practice, clinical procedures, and evaluation. Projection appraised the model’s capacity to transcend and influence the broader landscapes of health, science, and society. Qualitative suggestions were also collected to refine the proposal. Expert participation was voluntary and bound by formal confidentiality agreements.

Data were compiled into an analytical matrix following Lawshe’s approach as modified by Tristán [30]. To calculate essentiality, Likert-scale ratings were recoded into three response categories: ratings of 4 were classified as “essential”, ratings of 3 as “useful but not essential”, and ratings of 1 or 2 as “not necessary”.

The modified *CVR′* for each item was calculated using the following formula: 

*CVR′* = *n_e_*_/*N*_. Where *CVR′* represents the modified ratio proposed by Tristán [30], *n_e_* is the number of experts who classified the item as essential, and *N* is the total number of participating experts (*N* = 11 experts).

Values were summarized by dimension, and the overall Scale-Level *CVI* was computed as the average of the *CVR′* values across all evaluated items. Although Tristán’s approach establishes a minimum acceptance threshold of 0.27 for a panel of 11 judges [30], a more conservative minimum threshold of 0.58 was adopted in this study to maximize methodological rigor. Only items meeting or exceeding this cutoff were retained. Finally, qualitative feedback was analyzed and integrated to refine the structural components of the model.

### 2.3. Integration of Phases I and II

In Phase 1, qualitative data derived from empirical evidence and experiential knowledge were synthesized to develop the preliminary model. This model was subsequently refined in Phase 2 through quantitative content validation and expert feedback, which served as an external check on the initial theoretical constructs. Consequently, the final model proposal reflects a rigorous, integrated two-phase execution of the exploratory sequential multimethod design. In pursuit of maximum methodological rigor, international reporting guidelines—specifically the GRAMMS and PRISMA-ScR statements—were utilized to audit and guarantee the transparency and completeness of the qualitative, quantitative, and integrated components [31] (See Supplementary Materials S1 and S2).

*Ethical considerations*

The institution’s Research Ethics Committee approved the study under Act 026-221121.

## 3. Results

### 3.1. Model Design

**Figure 1:**
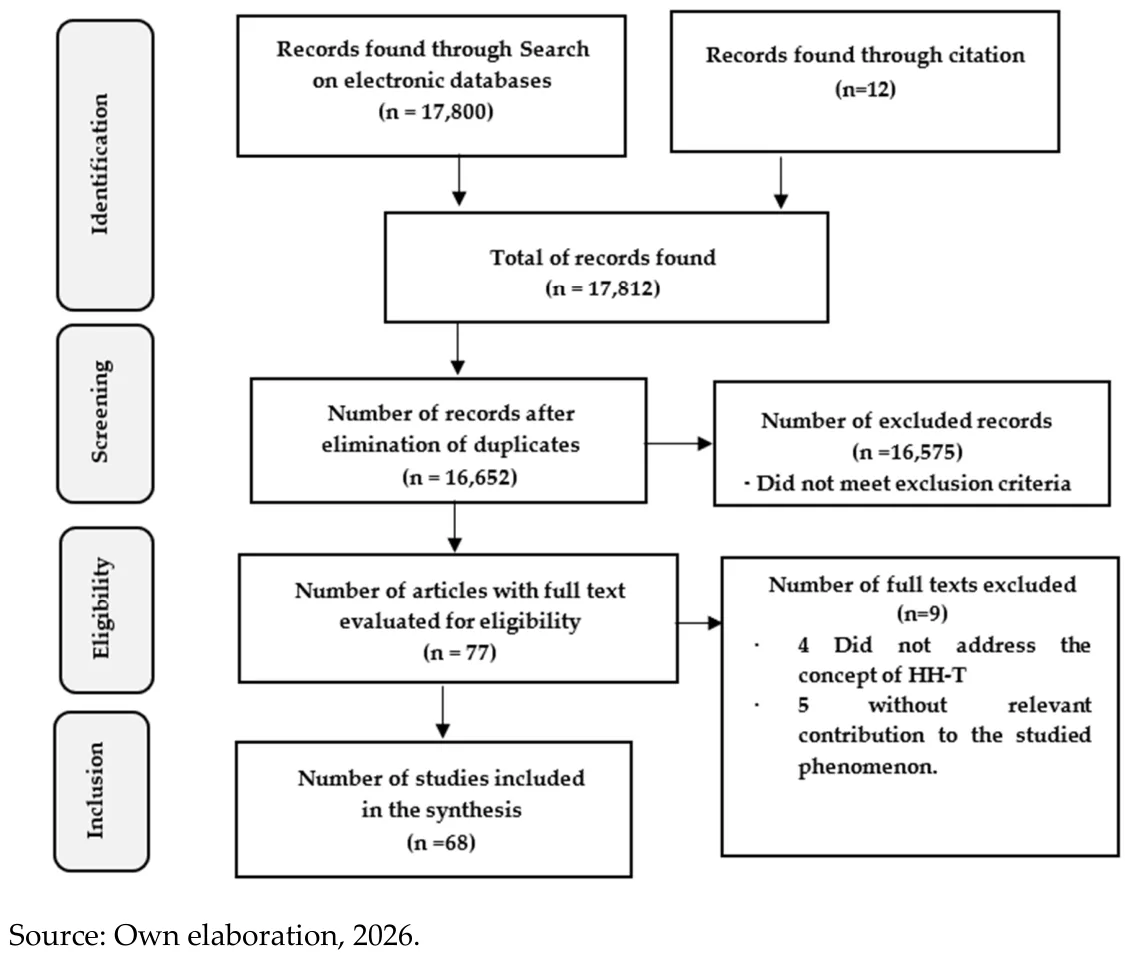
Flowchart of study selection and inclusion (adapted from the PRISMA statement for publishing scoping reviews).

The original version of the model comprised its metaparadigmatic concepts, assumptions, propositions, and hypotheses, which were subsequently refined and strengthened using the best available evidence. The literature search identified 17,812 records, of which 68 were included in the final analysis (see Figure 1).

The overall quality of the evidence was low, with only 10 of the 68 included studies classified within Levels 1 to 3 on the seven-level hierarchy of evidence. Geographically, the majority of studies originated from North America and Europe (see Supplementary Materials S3 and S4).

Based on the synthesized evidence, nursing practice guided by these models facilitates effective discharge planning and transitional support [32,33,34,35,36,37,38,39,40,41,42,43,44,45,46,47,48,49,50,51,52,53,54,55,56,57,58].

The primary goal of these frameworks is to optimize the well-being and quality of life of patient–family caregiver dyads [5,32,34,39,40,42,43,44,46,47,48,50,52,55,57,58,59,60,61,62,63,64,65,66,67,68,69,70,71,72].

To achieve this, the reviewed models delineate four core nursing functions designed to promote dyadic adaptation during the H-HT: (1) facilitating patient and family caregiver empowerment; (2) providing therapeutic support; (3) ensuring continuity of transitional care; and (4) advocating for the dyad (see Table 1).

Conceptual clarity in nursing practice facilitates seamless communication and collaboration among patient–family caregiver dyads, the healthcare team, and service providers [8,11,32,36,37,38,40,41,42,43,44,46,47,51,52,56,57,58,59,62,63,64,65,69,73,74,75,76,77,78,80,82,84,86,87,92,93,94]. Furthermore, it enhances clinical coordination across diverse levels of care [8,33,60,75,83,84] and drives the efficient utilization of digital health and communication technologies designed to optimize transitional care [64].

These models generate more robust clinical follow-up [10,32,33,34,36,42,47,48,55,57,58,59,60,61,62,64,72,74,75,76,78,84,86,87,89,92,93] and help establish favorable conditions for patient recovery [40,47,54,72]. Furthermore, they foster therapeutic relationships built on trust [8,37,38,43,51,52,57,73,79], increase access to appropriate health services [33,66,76,80], and ensure the delivery of timely, necessary, and continuous care [34,48,51,56,61,65,82,83,96].

Utilizing a theoretical framework to guide practice promotes dyadic autonomy—frequently conceptualized as self-care, self-management, self-efficacy, or self-control—for both patients and family caregivers [11,32,33,35,36,37,40,41,42,43,44,45,47,50,52,58,59,60,62,65,67,68,69,70,71,74,75,76,82,83,84,88,89,90]. Additionally, it supports dyads by providing appropriate information and tailored education throughout the transitional trajectory [10,11,32,34,35,36,37,40,41,42,43,44,47,48,49,50,51,52,54,55,57,59,61,62,63,64,65,66,67,69,72,73,74,75,76,77,78,79,80,82,83,84,96].

Model-guided practice during the H-HT effectively reduces hospital readmissions [10,36,37,46,50,55,57,62,64,67,69,74,81,82,87,92,96] and emergency department visits [43,44,61,62,63,64,69,76,82,89]. Furthermore, it shortens the length of hospital stays [33], decreases personal, social, and economic costs [10,11,32,40,52,56,57,59,61,63,64,68,69,70,71,72,74,76,84,89,92], and supports the optimization of healthcare resources [34,36,43,69]. Ultimately, utilizing a conceptual model allows the nursing profession to achieve greater clinical visibility and more robust evaluation of transitional care outcomes through structured performance indicators, thereby enabling continuous quality improvement [34,40,44,51,61,63,64,74].

Overall, the reviewed evidence characterizes the adaptation of patient–family caregiver dyads during the H-HT as a multidimensional phenomenon requiring integrative, theoretical, and practical approaches. Notably, this body of evidence reveals a critical scarcity of transitional nursing care models specifically developed or validated within the Latin American context.

All analyzed studies and their specific conceptual contributions to this framework are detailed in the Supplementary Files (See Supplementary Materials S3 and S4).

### 3.2. Model Overview

#### 3.2.1. Name

The nursing model designed to guide the adaptation of the patient–family caregiver dyads during the H-HT is called the *Adaptarte Model*. This name emphasizes its purpose of promoting adaptation in the care provided to these individuals.

#### 3.2.2. Emergence

The *Adaptarte Model* emerged within the framework of a Social Innovation project, as a response to a problem identified in society: the high vulnerability of patient–family caregiver dyads during the H-HT. This situation is often exacerbated by the lack of a structured guide that, in addition to providing guidance, allows for the continuous evaluation and improvement of nursing care, thus facilitating the adaptation for patient–family caregiver dyads during this process.

#### 3.2.3. Conceptual Framework for Development

This model is based on a humanistic philosophy that views individuals as unique, free, autonomous, and transcendent beings in a changing context. It conceptualizes nursing as a professional discipline dedicated to healthcare, from which it can contribute positively to society.

#### 3.2.4. Purpose of the Model

The *Adaptarte Model* aims to guide nursing practice during the caregiving phase of patient–family caregiver dyads, providing a structural framework for this practice. The expected outcomes are made explicit through the proposed effects and impacts on the individuals comprising the patient–family caregiver dyads, including improved clinical outcomes and reductions in complications, readmissions, and perceived burden.

#### 3.2.5. Scope of Application

The *Adaptarte Model* provides a theoretical framework designed to guide nursing research and transitional care practices for patient–family caregiver dyads within the Latin American healthcare context. Following future empirical validation, its scope encompasses nursing practice, research, and policy formulation tailored to regional caregiving contexts.

#### 3.2.6. Core Concepts of the Model

- *The subject of care*: Within the H-HT, the primary subject of care is the patient–family caregiver dyads. The patient is defined as the individual who has been admitted and received inpatient clinical care, with subsequent discharge to return home. The caregiver is an individual who provides care for the patient and is linked to them by kinship or a close personal relationship. Furthermore, the nursing professional is recognized as a complementary subject of care, highlighting the importance of creating conditions that support their professional well-being and personal well-being while facilitating adaptation within the patient–family caregiver dyad.
- *The context of the H-HT*: This concept encompasses the continuum between the hospital and the home environment. This transition period commences upon hospital admission and extends to 30 days post discharge. This phase accounts for shifts in clinical conditions and health service utilization. Within this context, the model seeks to ensure continuous, high-quality care through a collaborative network involving the dyad, the interdisciplinary team, healthcare institutions, community networks, and the broader healthcare system.
- *The nursing role*: Nursing facilitates adaptation within patient–family caregiver dyads by integrating scientific evidence with compassionate care. The professional role operates through four core functions: (a) psychoeducation and coaching to foster dyadic capability; (b) therapeutic accompaniment to motivate and support the dyad; (c) care coordination and longitudinal monitoring to guarantee transitional continuity; and (d) system navigation and advocacy to safeguard the dyad’s needs across the healthcare network.
- *The health or goal of nursing*: The primary objective is to promote adaptation in the patient–family caregiver dyads. This objective focuses on strengthening the dyad’s capacity to comprehend health challenges, provide mutual support, mitigate risks, and operationalize therapeutic instructions to ensure treatment adherence and effective health monitoring.

**Figure 2:**
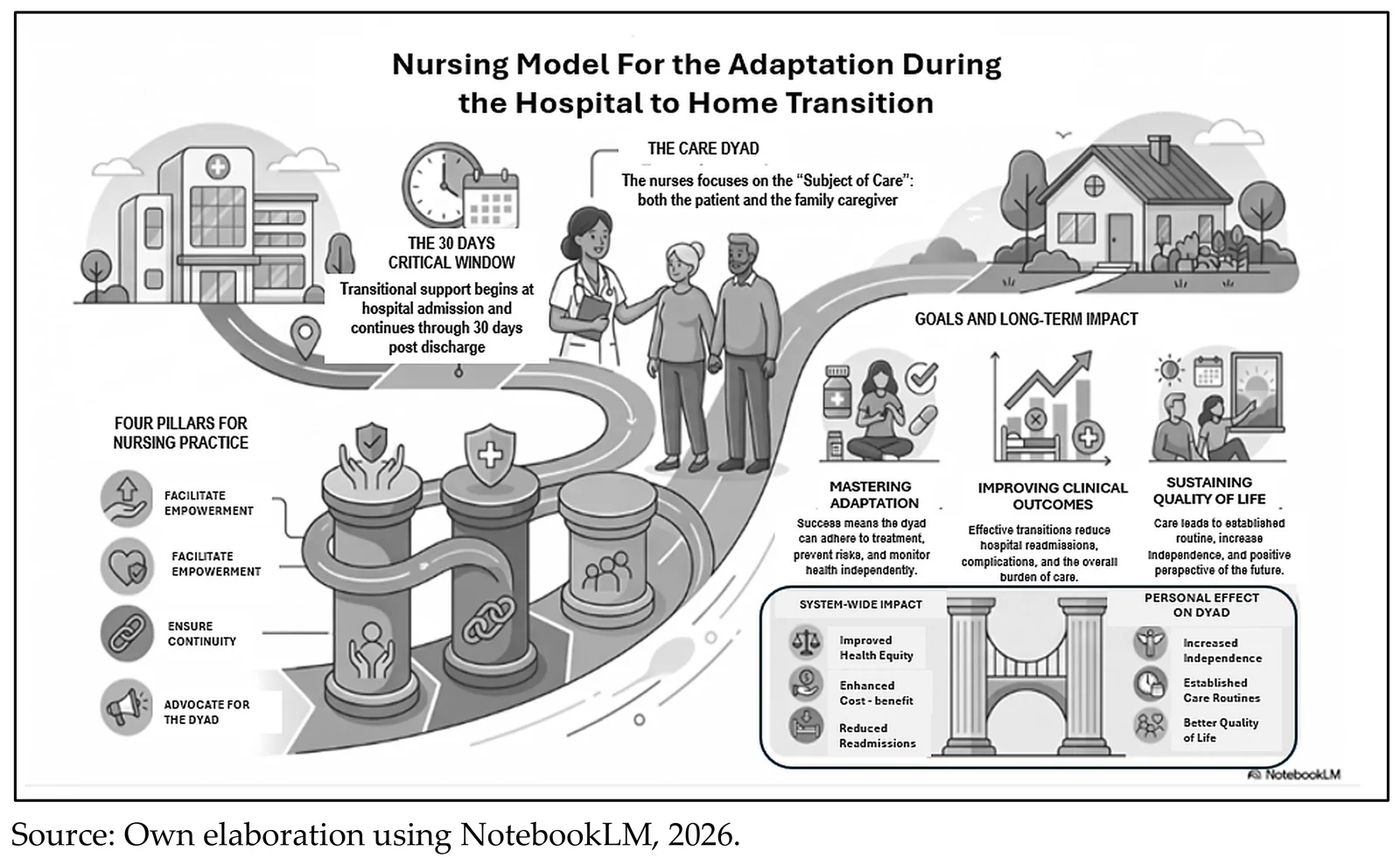
Graphic illustration of the *Adaptarte Model*.

The figure represents the dynamic nature of the *Adaptarte Model* in guiding adaptation in the patient–family caregiver dyads during the H-HT, where interactions among care recipients, the environment, and nursing actions generate positive effects and impacts throughout the process (see Figure 2).

#### 3.2.7. Theoretical Structure of the *Adaptarte Model*

The *Adaptarte Model* constitutes an organized conceptual system designed to underpin and explain the adaptation process in the patient–family caregiver dyads. This structure is composed of foundational assumptions, relational propositions, and testable hypotheses, which collectively form a logical framework for interpreting the adaptation during the H-HT.

- Assumptions: These establish fundamental beliefs and ontological statements regarding the nature of the individual, health, the environment, and nursing care. The model identifies six core assumptions that characterize the H-HT as a dynamic process of change, requiring continuous adaptation by the dyad in constant interaction with nursing professionals and their environmental context.
- Propositions: These statements articulate the relationships between the model’s central constructs. Nine propositions are defined to guide nursing practice toward measurable outcomes in health status, patient–family caregiver dyad experience, and clinical efficiency. These propositions serve as the basis for designing targeted nursing interventions and establishing evaluative indicators for the adaptation process.
- Hypotheses: Derived directly from the theoretical propositions, the *Adaptarte Model* proposes seven hypotheses articulated across multiple levels of analysis to guide future empirical research and evidence-based development in transitional care (see Table 2).

#### 3.2.8. Utility of the Model

The *Adaptarte Model* is designed to enhance clinical practice, health administration, and the pedagogical training of nursing professionals. Furthermore, it has the potential to stimulate the generation of new knowledge and provide a theoretical foundation for the development of public policies and regulatory frameworks. However, these applications are contingent upon the model’s complete validation and subsequent empirical testing across diverse healthcare settings and cultural contexts, particularly within Latin America. Once fully validated, the model may contribute to institutionalizing strategies that promote optimal adaptation in patient–family caregiver dyads during the H-HT.

### 3.3. Model Content Validation

#### 3.3.1. Experts’ Characteristics

Of the eleven experts summoned, all responded positively to the request to validate the model. They included professionals from engineering, medicine, and nursing. All had postgraduate training and between 5 and 30 years of experience in transitional care, working with or as part of nursing teams. They were from Chile, Mexico, Peru, and Colombia (See Supplementary Material S5).

#### 3.3.2. Content Validation Results

The experts’ evaluation of each of the model dimensions reflected a *CVR′* ranging from 0.90, in “support from empirical evidence”, to 0.99, in “Scope of application”. For the overall model, the *CVI* was 0.96 (See Table 3).

Similarly, the experts’ ratings for the model’s functionality and projection averaged 0.88. The highest score, 0.91, was achieved for three criteria: “Support the generation of new knowledge,” “Support for the generation of public policy,” and “Level of external coherence within a specific context.” Conversely, the lowest score, 0.82, was recorded for the criterion “Support for generation of structural indicators” (see Table 4).

The content validation process served not only to quantify expert agreement but also to support the systematic refinement of the *Adaptarte Model.* The experts’ qualitative comments informed revisions aimed at improving conceptual clarity, terminology, internal consistency, and logical organization. Suggestions considered theoretically congruent were incorporated through iterative discussions among the research team, resulting in the optimized final version of the model while preserving its core conceptual foundations.

Based on the qualitative analysis of these expert observations, the model was substantially refined to enhance its conceptual robustness and clinical applicability. The panel’s recommendations focused on strengthening the epistemological foundation, refining core concepts, broadening the framework’s scope to encompass the Latin American context, and addressing transferability limitations. Furthermore, the judges suggested optimizing practical utility by developing an implementation guide, integrating evaluative metrics, incorporating longitudinal strategies, and making structural adjustments to the diagram to visually clarify the operational workflow. All expert feedback was thoroughly addressed and integrated into the final version of the *Adaptarte Model* (See Supplementary Material S6).

## 4. Discussion

This study proposes the *Adaptarte Model*—a middle-range nursing theory—and establishes its content validity as a conceptual framework intended to inform future nursing practice and facilitate adaptation within patient–family caregiver dyads during the H-HT. Developed specifically to address the socio-institutional realities of the Latin American context, this theoretical framework offers a contextualized contribution to health systems research while providing testable propositions subject to future empirical verification and global testing. In addressing contemporary social demands, the model responds to urgent imperatives to optimize transitional care and advance frameworks for safe, humanized care transitions [1,2,21].

Grounded in its epistemological foundations, the *Adaptarte Model* adopts a constructivist and integrative approach wherein knowledge is generated through the synthesis of empirical evidence, clinical expertise, and the socio-cultural context of care. Consequently, the model proposes theoretical links between nursing actions and measurable outcomes—spanning health status, dyadic experience, and systemic efficiency—thereby offering a preliminary framework for future empirical evaluation through targeted clinical indicators [97].

By establishing the patient–family caregiver dyad as the primary, indivisible unit of care, the model defines the transition period as commencing upon hospital admission and extending through 30 days post-discharge. This timeline aligns with international standards for managing the peak risk period for post-acute complications, readmissions, and caregiver strain [98,99], encompassing a critical window of dynamic mutual adjustment where the adaptive responses and well-being of both dyad members directly shape transitional health outcomes.

From a conceptual standpoint, the *Adaptarte Model* views adaptation during H-HT dual-dimensionally: as both a process and an outcome. As a process, it aligns with Meleis’s Transition Theory regarding how awareness, commitment, and environmental changes influence the lived experience [2]. As an outcome, it echoes Roy’s Adaptation Model, where health is conceptualized as successful adaptation [100]. The *Adaptarte Model* integrates both perspectives by conceiving adaptation as a dynamic process leading to observable improvements in quality of life and dyadic well-being, confirming the conceptual complementarity of these foundational theories while establishing a platform for subsequent empirical testing.

The theoretical grounding of the model integrated global empirical evidence with clinical expertise. Although most primary studies originated in North America and Europe, our objective was not to replicate models built for highly resourced infrastructures. Rather, we extracted universal adaptation processes and interpreted them through the lens of Latin American healthcare systems, specifically addressing structural resource constraints, system fragmentation, and the central reliance on informal caregiving. Rather than invalidating the framework, the scarcity of regional evidence underscores the critical need for a contextualized theoretical model, the *Adaptarte Model*, to be empirically tested, evaluated, and refined within Latin American populations.

The central theoretical novelty of the *Adaptarte Model* lies in four analytical views. From an ontological perspective, the model establishes the patient–family caregiver dyad as the primary, indivisible, and interdependent unit of care, defining adaptation as a synchronous process wherein the health and adaptive capacity of each member directly condition those of the other. Moreover, the Model explicitly conceptualizes the nurse as a relational subject of care whose physical, emotional, and professional well-being constitutes an essential prerequisite for optimal clinical delivery. From an ecological-contextual perspective, unlike existing models that treat socio-institutional constraints as peripheral variables, the *Adaptarte Model* incorporates system fragmentation and structural scarcity of formal support networks as core determinants of the transitional process within Latin America. From a praxiological perspective, the model delineates prescriptive theoretical mechanisms to operationalize theory into practice by identifying specific, autonomous nursing actions designed to mediate dyadic adaptation. Finally, from an evaluative perspective, the model proposes a multi-level assessment framework that links proximal effects, including the achievement of dyadic adaptive goals, to projected distal impacts on organizational performance and health system outcomes, providing a comprehensive roadmap for future empirical testing.

To establish the conceptual contribution of the *Adaptarte Model*, four established nursing and transitional care frameworks were systematically compared across the same analytical perspectives.

Meleis’ Transitions Theory: Provides a comprehensive foundation for transition conditions and response patterns; however, it lacks an explicit focus on simultaneous reciprocal dyadic adaptation and Latin American healthcare dynamics [2]. The *Adaptarte Model* extends Meleis’ work by conceptualizing adaptation as a multidimensional, reciprocal process co-experienced by patients, caregivers, and nurses within regional health systems.

Roy’s Adaptation Model: Offers a robust framework for individual adaptive responses, yet its primary unit of analysis remains the individual rather than the structural interdependence of the dyad [100]. *Adaptarte* expands Roy’s perspective by positioning the patient–family caregiver dyad as the central unit of care.

Naylor’s Transitional Care Model: Demonstrates high efficacy in care coordination and readmission reduction within high-income health systems, but views caregivers primarily as supportive resources [43,65]. *Adaptarte* builds on Naylor’s operational strengths while embedding Latin American sociocultural contexts and treating both dyad members as equal, interdependent participants.

Coleman’s Care Transitions Intervention: Successfully drives patient activation and self-management coaching, though its focus remains patient-centric [101]. *Adaptarte* incorporates family caregiving as a structural, non-negotiable component of successful transitions tailored to regional realities.

Across all four dimensions, existing frameworks offer robust individual mechanisms or high-income clinical tools, whereas the *Adaptarte Model* addresses a key theoretical gap: an empirically designed, context-sensitive framework prioritizing reciprocal dyadic adaptation, with formal empirical validation identified as its essential next phase.

The structural integrity of the *Adaptarte Model* was confirmed through formal expert content validation, a fundamental prerequisite for internal coherence and structural validity in theoretical modeling [102,103]. The expert panel evaluation demonstrated high consensus regarding clarity, coherence, relevance, and sufficiency, while strong functionality scores suggest robust conceptual and potential practical applicability [102,104]. Specifically, the tautological, teleological, and visual representations of the model were validated as concise and logical [102]. Furthermore, the model reflects a circular flow connecting subjects, environment, transition process, and nursing actions, thereby capturing the non-linear, dynamic nature of adaptation [2].

The flexible structure of the *Adaptarte Model* enables nurses to adapt interventions across varying levels of care complexity, facilitating gradual implementation across diverse healthcare networks [105,106]. Derived from teaching-assistance partnerships, the framework bridges research, education, administration, and clinical practice [102]. While explicitly defining nursing’s autonomous disciplinary role, its primary collaborative contribution lies in articulating interdisciplinary care during H-HT, fostering effective communication across families, healthcare teams, institutions, and society [14].

To operationalize the *Adaptarte Model* for empirical verification, its hypotheses are structured across three complementary levels—dyadic, organizational, and health-system—serving as a progressive research roadmap rather than a framework to be validated in a single study. Proximal dyadic outcomes such as adaptation or quality of life can be evaluated directly through clinical trials or longitudinal designs, whereas distal macro-level impacts require broader, longitudinal health services methodologies. Furthermore, the model does not posit direct causal links between dyadic adaptation and distal outcomes; instead, it proposes that successful adaptation contributes to these broader outcomes through complex, multifactorial mechanisms. This multilevel structure provides a coherent theoretical framework to guide future empirical research and clinical application.

Future studies should operationalize the model’s projected outcomes using validated psychometric and clinical instruments, such as the Zarit Burden Interview for caregiver strain [107], the SF-36 for health-related quality of life [108] and established functional assessment scales for autonomy [109,110]. To support subsequent clinical translation, the development of a formal implementation guide is recommended to outline phased adoption, professional roles, interdisciplinary protocols, indicator tracking, and leadership development [34,40,42,44,46,52,55,57,58,59,61,63,69,70,71,72]. Longitudinal studies expanding the model into specialized clinical domains—such as palliative care and rehabilitation—will further fortify its empirical foundation, progressively consolidating the *Adaptarte Model* as a comprehensive theoretical framework for transitional care.

### Study Limitations

While the *Adaptarte Model* meets the criteria for theoretical construction as a middle-range nursing theory intended to integrate disciplinary considerations with practice and context during the H-HT in Latin American settings, its development is subject to certain limitations. First, the underlying literature retrieved during the conceptual synthesis remains constrained in scope and exhibits substantial heterogeneity in outcome measures, hindering direct comparisons across studies. Structurally, the published evidence presents a fragmented conceptualization of the H-HT process, lacking a unified theoretical framework to integrate its diverse components. Moreover, most available studies originate from North America and Europe, representing health systems and cultural contexts that differ significantly from Latin American realities. Additionally, the exclusion of grey literature, non-peer-reviewed sources, and inaccessible full texts may have led to the omission of regionally relevant, practice-based evidence not typically captured in traditional peer-reviewed indexing.

Regarding evidence quality and theory derivation, the predominance of descriptive and qualitative literature retrieved during the initial scoping review reflects the current stage of theoretical maturity surrounding transitional care in the region rather than a structural flaw in concept extraction. To mitigate potential bias during theory generation, critical appraisal informed a nuanced conceptual synthesis rather than arbitrary study exclusion. Core theoretical propositions remain firmly grounded in methodologically robust studies and were triangulated with primary insights from clinical practitioners. Because middle-range theories derived from heterogeneous literature establish a foundation for future empirical verification, the testable hypotheses arising from the *Adaptarte Model* will undergo rigorous testing in subsequent clinical trials and observational studies.

A methodological constraint of the content validation phase relates to the sample size and regional concentration of the expert panel. Although the panel fulfilled rigorous academic criteria and met established psychometric standards for content validation, its composition represents a potential constraint on immediate external validity and transferability. Furthermore, the non-probability, purposive sampling strategy employed introduces an inherent risk of selection bias that could narrow the diversity of perspectives represented. Consequently, this study represents an initial content validation phase; future research should employ multi-round Delphi processes with expanded, multidisciplinary, and international panels to strengthen consensus, perform construct and predictive validation, and enhance the model’s external validity across diverse healthcare systems.

Finally, although the *Adaptarte Model* demonstrated strong content validity through expert evaluation, it has not yet undergone construct, predictive, or direct clinical validation. Therefore, the proposed relationships among its concepts, assumptions, propositions, and operational hypotheses remain to be empirically tested in clinical practice. While the initial propositions of the model offer a contextualized framework, generating additional empirical evidence, training qualified personnel, and securing institutional resources remain imperative to strengthen its credibility, demonstrate its clinical utility, and facilitate successful deployment within Latin American health systems.

## 5. Conclusions

The *Adaptarte Model* fulfills the theoretical criteria for a middle-range nursing theory, offering a contextualized conceptual framework designed to inform nursing practice and promote adaptation within patient–family caregiver dyads during the H-HT. By establishing the dyad as the primary, indivisible unit of care and providing a structured approach to transition, the model lays a preliminary foundation for interdisciplinary alignment and future health system optimization tailored to the complexities of Latin American healthcare settings.

The methodological derivation of the model integrating empirical evidence, clinical expertise, and formal expert content validation demonstrates robust clarity, coherence, relevance, sufficiency, and structural functionality. Furthermore, the model makes visible the central, autonomous role of the nursing professional in mediating adaptation as both a dynamic process and a measurable outcome. In defining these theoretical linkages, the *Adaptarte Model* reinforces nursing’s disciplinary identity while articulating a collaborative framework to enhance continuity, safety, and humanized care during critical transition periods.

As a newly proposed middle-range theory, the model represents an essential starting point rather than a finalized clinical solution. Realizing its full potential will require generating empirical evidence through systematic testing across diverse clinical environments. Developing specialized implementation protocols, expanding international content validation, and conducting subsequent clinical trials will be paramount to establishing its construct validity, evaluating its distal impacts, and progressively consolidating its utility as a comprehensive guide for nursing practice, education, and health systems research.

## Figures and Tables

**Table 1:** Nursing main functions in promoting adaptation of the patient–family caregiver dyads during H-HT.

Nursing Functions	Core Components	Component Description	Supporting Evidence
Facilitating the empowerment of the patient–family caregiver dyads	Education	Teaching patient–family caregiver dyads about illness, treatments, care procedures, self-care, and caregiving roles to strengthen knowledge and skills.	[11,33,36,38,40,44,48,50,51,55,57,58,60,62,63,67,68,69,71,72,73,74,75,76,77,78,79,80,81,82,83]
	Demonstration, involvement, and coaching	Showing proper care routines, engaging patient–family caregiver dyads in direct activities and providing guidance throughout the process to foster greater confidence, skill, and independence	[11,33,34,36,37,43,49,52,57,58,68,69,74,78,84,85]
	Information, communication, and guidance	Delivering clear, timely, and understandable information about health status, procedures, expectations, and decision-making, helping patient–family caregiver dyads reduce uncertainty and improve understanding.	[8,32,33,36,37,41,43,47,48,50,51,53,55,57,61,63,71,72,74,77,79,83,84,86,87]
Providing therapeutic support	Facilitating care and providing accompaniment	Supporting patient–family caregiver dyads in accessing resources, performing care activities, and coping with hospitalization by easing care tasks with continuous presence and assistance.	[8,33,35,36,37,38,42,43,45,47,51,57,58,60,67,70,71,72,74,75,77,79,80,81,82,83,84,85,88,89,90]
	Inspiring and motivating through the caring relationship	Promoting hope, emotional strength, and confidence through a therapeutic, empathetic, and supportive nurse–patient–family caregiver dyad relationship.	[8,38,44,47,53,60,68,69,74,83,89,91]
Ensuring continuity of transitional care	Monitoring and follow-up	Continuously assessing care processes, patient–family caregiver dyads adaptation, and goal achievement to identify problems and adjust nursing interventions.	[32,35,40,48,49,55,57,63,65,69,72,74,75,76,78,80,84,85,87,89,92,93]
	Coordination of services, reporting, and referral	Organizing and integrating health services, communicating relevant information to the care team, and making timely referrals to ensure continuity, safety, and quality of care.	[8,11,36,38,39,40,41,43,44,45,46,47,51,52,54,55,56,57,58,59,60,61,62,63,67,68,69,70,71,72,73,74,76,77,78,80,81,82,83,84,86,87,88,89,92,94,95]
Advocating for the dyad	Advocacy and direct support	Defending the rights and needs of patient–family caregiver dyads, supporting decision-making, and promoting policies or actions that help them adapt to the caregiving role.	[44,49,68,73,77,78,79,84,89,94]

Source: Own elaboration, 2026.

**Table 2:** Theoretical structure of the *Adaptarte Model*.

Theoretical Component	Component Description	
**Assumptions**	1. The individuals who comprise the patient–family caregiver dyads are primarily responsible for their own health care. 2. The individuals who comprise the patient–family caregiver dyads seek to adapt during the transition to a family home. 3. Self-care can be learned during the transition to the family home. 4. Adapting to caregiving during the transition to a family home is a process that involves preparation, participation, and acceptance. 5. Adaptation in the patient–family caregiver dyads during the H-HT occurs within a changing context. 6. During the H-HT, nursing support facilitates a safe, autonomous, and well-being-focused transition.	
**Propositions**	1. Adaptation as a Dynamic Process: Adaptation during the H-HT is a multifaceted and dynamic process characterized by continuous adjustments. This process is influenced by individual attributes, clinical conditions, and environmental contexts, often manifesting as periods of stress and uncertainty. 2. Restoration of Equilibrium: Successful transition requires that the patient–family caregiver dyads reassume agency and establish new normalcy, which is essential for optimizing health-related quality of life. 3. Institutional Support for Nursing: The efficacy of the nursing role in facilitating H-HT adaptation is contingent upon supportive institutional conditions that enable nurses to perform essential coordination and clinical support functions. 4. Continuity and Interdisciplinary Coordination: Adaptation necessitates a continuum of high-quality care. Within this framework, the nurses act as the primary coordinator, aligning the dyad with the interdisciplinary team, healthcare institutions, and broader community networks. 5. Facilitation of Adaptation: During the H-HT, the nurse facilitates dyadic adaptation through targeted interventions, including information, health education, motivational support, clinical demonstration, simplification of care, advocacy, and systematic monitoring and referral to specialized services. 6. Mechanisms of Coping: Adaptation during the H-HT requires the dyad to employ effective coping strategies, comprehend complex care instructions, leverage social support networks, adhere to prescribed treatment plans, and proactively monitor health status to anticipate and prevent complications. 7. Impact on Health Outcomes: Enhanced adaptation within the patient–family caregiver dyads promotes positive clinical outcomes and prevents avoidable complications. 8. Promotion of Autonomy and Well-being: Positive adaptation is achieved through continuous care that fosters the dyad’s autonomy, facilitates the re-establishment of daily routines, increases satisfaction with care milestones, and promotes a resilient outlook on the future. 9. Systemic and Clinical Efficiency: Optimal adaptation during the H-HT yields systemic benefits, including enhanced clinical outcomes, improved experiences for both dyads and professionals, improved equity and cost-effectiveness, and a significant reduction in readmission rates and caregiver burden.	
**Hypotheses**	Dyadic Level (Proximal clinical and psychosocial outcomes evaluated directly)	1. Clinical Outcomes: Higher levels of adaptation within the patient–family caregiver dyads during the H-HT are positively correlated with improved clinical outcomes for the patient. 2. Dyadic Experience: A positive healthcare experience for the patient–family caregiver dyads is significantly associated with their degree of adaptation during the H-HT. 3. Complication Rates: Increased adaptation in the patient–family caregiver dyads during the H-HT is inversely associated with the incidence of health complications. 4. Caregiver Burden: Optimal adaptation in the patient–family caregiver dyads during the H-HT is inversely correlated with the perceived burden of care experienced by the family caregiver.
	Organizational Level (Distal outcomes related to staff and service delivery)	5. Professional Meaning: Optimal adaptation of the patient–family caregiver dyads during the H-HT is associated with higher levels of professional satisfaction and a more favorable healthcare experience for the nursing and interdisciplinary staff.
	Health-System Level (Macro distal outcomes evaluated via longitudinal and health services designs)	6. Economic Efficiency: Greater adaptation within patient–family caregiver dyads during the H-HT is associated with an improved cost–benefit ratio for the healthcare system. 7. Resource Utilization: Higher levels of adaptation in the patient–family caregiver dyads during the H-HT are associated with a reduction in hospital readmissions and a decrease in the occupancy of hospital beds.

Source: Own elaboration, 2026.

**Table 3:** Results of the Content Validation of the Model.

Model Dimension		Expert Rating of Content Components				*CVR′*
		Clarity	Coherence	Relevance	Sufficiency	
**Attributes**	Name	3.91	3.91	4.00	3.68	0.97
	Definition of concepts	3.82	3.91	3.91		0.96
	Statement of assumptions	3.64	3.91	4.00		0.95
	Logical propositions	3.82	3.82	3.91		0.95
	Statement of hypotheses	3.82	3.82	3.91		0.95
	Illustrative diagram	3.64	3.91	3.91		0.95
**Relational significance**	Underlying philosophy	3.73	4.00	4.00	3.81	0.97
	Purpose of the model	3.91	4.00	4.00		0.98
	Scope of application	4.00	4.00	4.00		0.99
	Usefulness of the model	3.91	4.00	4.00		0.98
**Rigor**	Support from theoretical evidence	3.64	3.91	4.00	3.54	0.94
	Clarity of limitations	3.82	4.00	4.00		0.96
	Support from empirical evidence	3.45	3.73	3.73		0.90
	*CVI*					0.96

Abbreviations: *CVR′* = modified Content Validity Ratio proposed by Tristán; *CVI* = Content Validity Index. Source: Own elaboration, 2026.

**Table 4:** Results of the functionality and projection validation.

Condition	Expert Ratings of Functionality and Projection	*CVR′*
Support the generation of new knowledge	3.64	0.91
Support for the generation of public policy	3.64	0.91
Support for generation of structural indicators	3.27	0.82
Support for generation of process indicators	3.36	0.84
Support for generation of outcome indicators	3.55	0.89
Support for generation of impact indicators	3.55	0.89
Level of internal coherence	3.36	0.84
Level of external coherence (within a specific context)	3.64	0.91
*CVI*	3.50	0.88

Abbreviations: *CVR′* = modified Content Validity Ratio proposed by Tristán; *CVI* = Content Validity Index. Source: Own elaboration, 2026.

## Data Availability

The data supporting the findings of this study are available from the corresponding author upon reasonable request. Data are not publicly available due to the qualitative nature of the information, which was collected under strict confidentiality agreements with the participants. However, anonymized data may be shared for academic research purposes upon request to the corresponding author, subject to institutional ethical approvals.

## Supplementary Materials

The following supporting information can be downloaded at: https://www.mdpi.com/article/10.3390/healthcare14182905/s1, Supplementary Material S1: GRAMMS checklist [111]; Supplementary Material S2: PRISMA ScR checklist; Supplementary Material S3: Databases and search strategy; Supplementary Material S4: Search results of models for patient–family caregiver dyads adaptation during the H-HT; Supplementary Material S5: Experts’ profile; and Supplementary Material S6: Expert feedback during the *Adaptarte Model* Content Validation.
