# Supplementary material for: Adaptation Model for Patient and Caregiver Dyads in Hospital-to-Home Transition: Theory Development and Content Validation
Source: Healthcare (Basel). 2026 Sep 8;14(18):2905. doi: 10.3390/healthcare14182905 (PMC13606408; doi:10.3390/healthcare14182905)
Supplement: Supplementary file 1 [file healthcare-14-02905-s001.zip › Supplementary material S1 18 09 2026.pdf]

**Supplementary Material S1. GRAMMS Reporting Criteria.**

| **GRAMMS Criterion** | **Focus Area** | **Checklist** | **Notes** |
|---|---|---|---|
| 1. Justification for mixed methods | State the justification for utilizing mixed methods in this study. | ✓ | Pg.4-6. |
| 2. Description of design | Describe the type, priority and sequence of the different methods of study employed. | ✓ | Pg.4-6. |
| 3. Methods detail | Describe the sampling approach for both quantitative and qualitative data collection and description of data analysis method for both methods. | ✓ | Pg.4 -6. |
| 4. Integration | State how the quantitative and qualitative data was integrated. | ✓ | Pg. 6. |
| 5. Method limitations | Identify the limitations associated with combining the two methods used for the study (mixed methods). | ✓ | Pg 19. |
| 6. Insights from integration | Provide details of any new knowledge gained through the integration of quantitative and qualitative data. | ✓ | Pg. 6; 16-20. |

Source: Own elaboration, 2026. Based on O'Cathain A, Murphy E, Nicholl J. *The quality of mixed methods studies in health services research*. J Health Serv Res Policy. 2008;13(2):92-98. doi:10.1258/jhsrp.2007.007074.
