# Supplementary material for: Adaptation Model for Patient and Caregiver Dyads in Hospital-to-Home Transition: Theory Development and Content Validation
Source: Healthcare (Basel). 2026 Sep 8;14(18):2905. doi: 10.3390/healthcare14182905 (PMC13606408; doi:10.3390/healthcare14182905)
Supplement: Supplementary file 1 [file healthcare-14-02905-s001.zip › Supplementary material S2 18 09 2026.PDF]

Supplementary Material S2. PRISMA Extension for Scoping Reviews (PRISMA-ScR) Checklist

**Section**	**Item**	**PRISMA-ScR Checklist Item**	**Check**
**Title**	1	Identify the report as a scoping review.	✓
**Abstract**	2	Provide a structured summary including background, objectives, eligibility criteria, sources of evidence, charting methods, results, and conclusions.	✓
**Introduction**	3	Describe the rationale for the review in the context of existing knowledge.	✓
	4	State the review objectives and/or questions.	✓
**Methods**	5	Indicate whether a review protocol exists and where it can be accessed (optional).	✓
	6	Specify the eligibility criteria for sources of evidence.	✓
	7	Describe all information sources (databases, websites, reference lists, etc.) and the date of the last search.	✓
	8	Present the complete electronic search strategy for at least one database so it can be replicated.	✓
	9	Describe the process for selecting sources of evidence (screening and eligibility).	✓
	10	Describe the data charting (data extraction) process.	✓
	11	List and define all variables for which data were sought.	✓
	12	If conducted, describe the critical appraisal of individual sources of evidence (optional).	✓
	13	Describe the methods used to synthesize the charted data.	✓
**Results**	14	Report the number of sources screened, assessed for eligibility, included in the review, and reasons for exclusions at each stage (preferably using a PRISMA flow diagram).	✓
	15	Present the characteristics of the included sources of evidence.	✓
	16	Present the results of the critical appraisal, if performed (optional).	✓
	17	Present the relevant data extracted from each included source of evidence.	✓

	18	Summarize and synthesize the main findings in relation to the review objectives and questions.	✓
**Discussion**	19	Summarize the main results, including key concepts, themes, and types of evidence identified.	✓
	20	Discuss the limitations of the scoping review process.	✓
	21	Provide an overall interpretation of the findings and their implications for research, policy, and/or practice.	✓
**Funding**	22	Describe the sources of funding for the included sources of evidence (if available) and for the scoping review itself, including the role of the funders.	✓

Source: Own elaboration, 2026. Based on the PRISMA -ScR guideline.
