# Supplementary material for: Adaptation Model for Patient and Caregiver Dyads in Hospital-to-Home Transition: Theory Development and Content Validation
Source: Healthcare (Basel). 2026 Sep 8;14(18):2905. doi: 10.3390/healthcare14182905 (PMC13606408; doi:10.3390/healthcare14182905)
Supplement: Supplementary file 1 [file healthcare-14-02905-s001.zip › Supplementary material S3 18 09 2026.PDF]

Supplementary Material S3. Databases and search strategy

1. **Results by Database and Reference Count**

Database	RoI in the search	Keywords	Boolean terms	Number of available articles (n)	Number of selected articles (n)	Retrieved references
PubMed / MEDLINE / PMC	Main source of high-impact clinical and nursing evidence	"Hospital to Home Transition", "Transitional Care", "Adaptation", "Nursing", "Patient", Caregivers".	[(Hospital to Home Transition OR Transitional Care) AND (Adaptation) AND (Nursing) AND (Patient OR Caregivers)].	164	63	Albert (2016); Alizadeh (2021); Allen (2014, 2017); Balaban (2015); Berthelsen (2023); Brown (2021); Bucknall (2020); Chen (2021); Cho (2015); De Regge (2017); Desai (2015); Eliassen (2023); Groenvynck (2021); Hestevik (2019); Hewner (2021); Hirschman (2015); Jones (2022); Kast (2021); Kim (2023); Le Berre (2017); Leithaus (2022); Liebzeit (2021, 2024); Malley (2015); Markiewicz (2019); Miller (2019); Mitchell (2018); Monkong (2020); Mora (2017); Morkisch (2020); Morrison (2016); Murray (2019); Naylor (2017, 2018); Nordmark (2016); O'Donnell (2021); Rathert (2015); Rezapour-Nasrabad (2018); Salehi (2021); Saragosa (2023);

						Solan (2015); Sun (2023); Takahashi (2020); Testa (2020); Thrasher (2018); Tutar (2022); Tyler (2019); Ulin (2016); Weiss (2015); Werner (2019); Wyer (2016); Xu (2024); Ye (2016); Zupanc (2020); Zwart (2021)
SciELO / Regional databases	Identification of Latin American and contextual nursing evidence	"Hospital to Home Transition", "Transitional Care", "Adaptation", "Nursing", "Patient", Caregivers".	[(Hospital to Home Transition OR Transitional Care) AND (Adaptation) AND (Nursing) AND (Patient OR Caregivers)].	88	4	Aued (2019); Costa MFBNA (2020); Dias da Silva Lima (2018); Feil Weber (2017)
Science-Direct (Elsevier)	Retrieval of articles published in high-impact journals	"Hospital to Home Transition", "Transitional Care", "Adaptation", "Nursing", "Patient", Caregivers".	[(Hospital to Home Transition OR Transitional Care) AND (Adaptation) AND (Nursing) AND (Patient OR Caregivers)].	5377	17	Albert (2016); Breneol (2017); Bucknall (2020); Comin-Colet (2016); Green (2025); Groenvynck (2021); Malley (2015); Miller (2019); Naylor (2018); Salehi (2021); Saragosa (2023); Takahashi (2020); Testa (2020); Thrasher (2018); Tutar (2022); Ye (2016); Zupanc (2020)
CINAHL / Embase /	Complementary	"Hospital to Home	[(Hospital to Home	12171	19	Chakurian (2021); Hirschman (2015);

Google Scholar/ other sources	search in nursing and extended literature	Transition", "Transitional Care", "Adaptation", "Nursing", "Patient", Caregivers".	Transition OR Transitional Care) AND (Adaptation) AND (Nursing) AND (Patient OR Caregivers)].			Lee (2017);<br>Mora (2017);)<br>Miller (2019);<br>Mitchell (2018);<br>Monkong (2020);<br>Nasrabad (2018);<br>Nordmark (2016);<br>O'Donnell (2021);<br>Paudel (2022);<br>Pollack (2016);<br>Rathert (2015);<br>Rezapour- Salehi (2021);<br>Testa (2020);<br>Thrasher (2018);<br>Tutar (2022);<br>Ye (2016);<br>Zupanc (2020)

Source: Own elaboration, 2026.

2. Results by duplicate screening

Database	Duplicate Status	References
1. PubMed / MEDLINE / PMC (n = 63)	Duplicate records	Albert (2016); Aued (2019); Breneol (2017); Bucknall (2020); Comin-Colet (2016); Dias da Silva Lima (2018); Groenvynck (2021); Hirschman (2015); Malley (2015); Miller (2019); Mora (2017); Naylor (2018); Salehi (2021); Saragosa (2023); Takahashi (2020); Testa (2020); Thrasher (2018); Tutar (2022); Weeks (2021); Ye (2016); Zupanc (2020)
	Unique records	Alizadeh (2021); Allen (2014, 2017); Balaban (2015); Berthelsen (2023); Brown (2021); Chakurian (2021); Chen (2021); Cho (2015); De Regge (2017); Desai (2015); Eliassen (2023); Hestevik (2019); Hewner (2021); Jones (2022); Kast (2021); Kim (2023); Le Berre (2017); Leithaus (2022); Liebzeit (2021, 2024); Markiewicz (2019); Mitchell (2018); Monkong (2020); Morkisch (2020); Morrison (2016); Murray (2019); Naylor (2017); Nordmark (2016); O'Donnell (2021); Rathert (2015); Rezapour-Nasrabad (2018); Solan (2015); Sun (2023); Tyler (2019); Ulin (2016);

		Weiss (2015); Werner (2019); Wyer (2016); Xu (2024); Zwart (2021)
2. SciELO / Regional databases (n = 4)	Duplicate records	Aued (2019); Dias da Silva Lima (2018)
	Unique records	Costa MFBNA (2020); Feil Weber (2017)
3. ScienceDirect (n = 17)	Duplicate records	Albert (2016); Breneol (2017); Bucknall (2020); Comin-Colet (2016); Groenvynck (2021); Malley (2015); Miller (2019); Naylor (2018); Salehi (2021); Saragosa (2023); Takahashi (2020); Testa (2020); Thrasher (2018); Tutar (2022); Ye (2016); Zupanc (2020)
	Unique records	Green (2025)
4. CINAHL / Embase / Google Scholar / other sources (n = 7 Directly identified articles)	Duplicate records	Chakurian (2021); Hirschman (2015); Mora (2017); Weeks (2021)
	Unique records	Lee (2017); Paudel (2022); Pollack (2016)
Consolidated summary of duplicates	Total articles	68
	Duplicate Articles (≥2 databases)	25
	Unique articles	43

Source: Own elaboration, 2026.
