# Supplementary material for: Adaptation Model for Patient and Caregiver Dyads in Hospital-to-Home Transition: Theory Development and Content Validation
Source: Healthcare (Basel). 2026 Sep 8;14(18):2905. doi: 10.3390/healthcare14182905 (PMC13606408; doi:10.3390/healthcare14182905)
Supplement: Supplementary file 1 [file healthcare-14-02905-s001.zip › Supplementary material S4 18 09 2026.pdf]

Supplementary material S4. Search results of models for patient- family dyads adaptation during the H-HT

1. **Search Results:**

Reference author, site, date	Objective	Method	Sample	Main results	Main conclusions	Applicability to the study	Quali-ty	Evi-dence
Albert, 2016 USA	To evaluate existing care transition models to identify common themes that minimize the exacerbation of rehospitalizations and improve the quality of life of patients with heart failure.	Systematic literature review	24 studies.	Models employed included:<br><br>Models that helped<br>The Bridge model<br>The Care Transitions model<br>The Enhanced Discharge Planning Program (EDPP)<br>Partners in Care for Congestive HF (PCCHF)<br>Postdischarge Care Transition (PDCT)<br>Project Better Outcomes for Older Adults Through Safe Transitions (BOOST)<br>Reengineering Design (RED)<br>State of Action on Avoidable Rehospitalization (STAAR) and the Transitional Care model.<br><br>Support includes:<br>1. Discharge planning<br>2. Communication and coordination among the healthcare team<br>3. Clear, organized, and timely information<br>4. Medication reconciliation and adherence<br>5. Community support networks and support groups<br>6. Monitoring and management of post-discharge signs and symptoms with patient education<br>7. Post-discharge follow-up<br>8. Care planning with alternatives for chronic conditions and palliative care.	When professionals operate within a comprehensive model, they help patients manage their condition and reduce readmissions.<br>Include practical skills<br><br>The models are aimed at patients and caregivers and take the healthcare team into account.<br><br>The models help offer services that promote knowledge and commitment to self-care and active communication with healthcare professionals.<br><br>Help save costs and reduce rehospitalizations	Subject: Patient, personal healthcare provider<br><br>Goal: Improve QOL<br><br>Context: Hospital-home transition<br><br>Nursing/Service<br>Follow-up<br>Include practical skills<br>Offer services that promote knowledge and commitment to self-care and actively communicate with healthcare professionals<br>Support includes:<br>1. Discharge planning<br>2. Communication and coordination among the healthcare team<br>3. Clear, organized, and timely information<br>4. Medication reconciliation and adherence<br>5. Community support networks and support groups<br>6. Monitoring and management of post-discharge signs and symptoms with patient education<br>7. Post-discharge follow-up<br>8. Care planning with alternatives for chronic conditions and palliative care<br><br>Consequences<br><br>Reduce readmissions and emergency department admissions	CASP 10/10	5

						Reduce costs<br><br>Other considerations<br><br>They highlight the usefulness of having a theoretical guide that generates practical benefits.		
Alizadeh et al., 2021 Iran & Sweden.	Developing a Hospital-to-Home Transitional Cancer Care Program in Iran's Healthcare System. //	Phased-stage health systems and policy research: 1) qualitative study through semi-structured interviews. 2) literature review of transitional care models. 3) preliminary program proposal; and 4) analysis of program validity and feasibility using the Delphi method. //	24 participants in the qualitative component and 8 experts in phase 1. 3 studies in phase 2. And 23 experts in phase 4.	// Six main categories were extracted from the qualitative outcomes, consisting of "integrated services for continuity of care," "holistic care," "standardization of care," "use of telemedicine," "transparency of rules," and "delivery of the care process." Using these results and the three common models of transitional care, the initial program was developed in three phases: pre-discharge, post-discharge, and transition care, with six protocols. The program's content validity (98.7%) and feasibility (95.8%) were approved by experts in the Delphi rounds.	A hospital discharge program coordinated with the home care center plan is essential in the provision of healthcare services for cancer patients.<br><br>It is essential to have:<br>1. Integrated services<br>2. Continuity of care<br>3. Holistic care<br>4. Standardization of care<br>5. Use of telemedicine<br>7. Transparency of regulations<br>8. Care delivery process	Subject: Patients and families with home care needs. There is a follow-up nurse.<br><br>Goal: Improve self-care and empower families.<br><br>Context: Begins with a pre-discharge assessment and includes the home, family, and community. Support tools are required to determine necessary services based on patient needs. Visits and follow-up are provided for 4 weeks. Records are shared between different levels.<br><br>Nursing/Service: Strengthen self-care through training. Communicate and follow up, refer as needed. Facilitate processes. Encourage patient and family participation. Provide support and visits to ensure continuity of care. Respond to concerns on an ongoing basis.<br><br>Consequences: Shorter hospital stays. Greater access to required services. Fewer hospital readmissions. Empowers families; increases patient self-efficacy.<br><br>Other considerations: The program has a pre-discharge, post-discharge, and transition phase. It includes the development of protocols for each phase.	MMAT 5/5	4
Allen et al. 2014 Australia.	To identify and synthesize research,	Systematic review across eight	12 RCTs published between 1990	The transitional care interventions reported in most studies reduced rehospitalization, except for the	Gaps in the evidence base were evident in the quality domains of timeliness, equity, efficiencies for	Subject: Person and Family	CASP 10/10	1

	using randomized controlled trial designs, on the quality of transitional care interventions compared with standard hospital discharge for older people with chronic conditions, with recommendations for research and practice.	databases. Quality indicators were reviewed in relation to a transitional care intervention with hospital discharge care and follow-up support at home.	and 2013 included people aged 60 and over undergoing hospital-to-home transition.	GP and primary care nurse models. All included rehospitalization and length of stay outcomes as quality measures. Patient satisfaction was assessed in only six and was high. There is limited information on patient and caregiver experience, caregiver burden and support, and emotional support for older adults and their caregivers. Outcome measures in some cases included timeliness, equity, efficiencies for community providers, and symptom management.	community providers, effectiveness/symptom management, and person- and family-centered care domains. There is a need to improve understanding and evidence on the quality of transitional care for older adults and their caregivers in particular domains of person- and family-centered care: patient and caregiver experience, caregiver burden and support, and emotional support for older adults and their caregivers during care transitions.	Goal: A positive experience for the patient and caregiver Effectiveness, equity, symptom management, timeliness, efficiencies. Reduce burden on the family<br><br>Context:<br>Transitions of Care<br>Nursing/Service<br>Person-centered care. Shared decisions with care recipients.<br><br>Consequences:<br>Reduced hospital readmissions<br><br>Other Considerations:<br>Six quality indicators were considered:<br>1. Effectiveness: Effective care is evidence-based and<br>is provided to those most likely to benefit<br>2. Efficiency: Efficient care or care without waste, including wasted resources<br>3. Timeliness: Timely care is care provided without long waiting periods for patients and their families<br>4. Safety and Risk: Care is low-risk and safe when it does not cause harm to patients, families, or healthcare staff 5. Equity: Care that is fair for all. No group of people receives inferior care based on differences in gender, culture, ethnicity, age, sexuality, geographic location, or socioeconomic status.<br>6. Person- and family-centered care and experience respects the preferences, values, and goals of patients and their families.<br>Care decisions involve patients and families.		
Allen et al., 2017 Australia	Improve understanding of the	Meta-synthesis	20 studies	Four themes emerged:<br>1) Trying to work together<br>2) Not reaching the goal	Greater participation and adaptation depend on the type of care provided.	Subject: Patients/caregivers and healthcare providers	CASP 10/10	5

	experience of older adults' users, caregivers, and healthcare providers; and the integration of care into the care of older adults transitioning from hospital to home.			3) An appropriate discharge<br>4) Somehow you adapt.<br><br>The transition is seen as a social process of negotiation and navigation that fluctuates between independence for older adults and their caregivers or dependence on healthcare providers.<br><br>Users participated through strategies of questioning, discussion, information gathering, information seeking, evaluation, and translation. //	Findings from the following models are confirmed:<br>1) U.S.-based Care Transitions Intervention (Coleman, Parry, Chalmers, & Min, 2006)<br>2) Transitional Care Model (Naylor et al., 1999; Naylor et al., 2004).	Goal: Achievement of autonomy for care, greater independence.<br><br>Context: Transition from hospital to home. The context influences the user's discharge experience.<br><br>Nursing/Service: Providing participatory, inclusive services with support and information, facilitating and evaluating achievements. Clarity of roles and responsibilities. Integration of care.<br><br>Consequences: More positive experience.<br><br>Other considerations: The experience of returning home should be matched with training and support.<br>//		
Aued et al 2019 Canada & Brazil	To describe the activities carried out by liaison nurses to ensure continuity of care after hospital discharge. //<br>.	Descriptive, qualitative study based on the theoretical framework "Strength-Based Care." Using an electronic semi-structured questionnaire and its content analysis.	23 liaison nurses. //	Professionals participate in:<br>1) identify patients who need care after hospital discharge.<br>2) coordinating and planning the hospital discharge with the transfer of clinical information to the outpatient service.	The activities carried out by liaison nurses:<br>Focus on the patient's needs.<br>Articulate outpatient services.<br>Adapted to the context to ensure continuity of care after hospital discharge.	Subject: Patient, family, and nurse as unique individuals.<br>Goal: ensuring continuity of care for people undergoing the hospital-to-home transition.<br>Context: The patient is part of a healthcare system and belongs to a family and a community; each contributes to the patient's recovery.<br>Nursing/Service<br><br>Person- and family-centered<br><br>Ensure continuity of care<br><br>Coordination based on needs<br><br>Educators<br><br>Articulators between professionals and services<br><br>Advocates for the patient<br><br>Transmits information	CASP 10/10	6

						Able to work as a team<br><br>Knowledgeable about in- and out-of-hospital resources<br><br>Competence in recognizing conditions that help achieve health<br><br>Consequences<br><br>Favorable conditions for recovery<br><br>Attitude of trust<br><br>Other considerations<br><br>Contribution to Latin American services.		
Balaban et al., 2015<br>USA	To determine whether an intervention by patient navigators (PNs), hospital-based community health workers, reduces readmissions among high-risk, low-socio economic status patients.<br>.	Randomized controlled trial.	585 people in the experimental group and 925 in the comparison group	Overall, 30-day readmission rates did not differ between intervention and control patients. However, the two age groups demonstrated marked differences. Intervention patients aged 60 years and older showed a statistically significant adjusted absolute decrease of 4.1% [95% CI: −8.0%, −0.2%] in readmissions with an increase in 30-day outpatient follow-up.<br>Intervention patients aged ≤60 years<br>showed a statistically significant adjusted absolute increase of 11.8% [95% CI: 4.4%, 19.0%] in readmissions, with no change at 30-day outpatient follow-up.	A patient navigator intervention among high-risk and safety-net patients reduced readmissions.	Subject: Patient, family, nurse, healthcare professionals.<br>Goal: Reduce readmissions of high-risk and low socioeconomic status individuals.<br>Context: Prepare for hospital discharge up to 30 days post-discharge and should only end upon return to primary care.<br>Nursing/Service: Guidance and assistance to navigate the transition from hospital to home through hospital visits and weekly telephone outreach, discharge preparation, medication management, scheduling follow-up appointments, communication with primary care, and symptom management.<br>Consequences: Fewer readmissions.<br>Outpatient follow-up rates.<br><br>Other considerations: The model showed greater utility and stability for individuals younger than 60 years of age.	MMAT 5/5	2

Berthelsen et al<br>2023<br>Denmark	To synthesize the combined evidence from systematic reviews of interventions using elements of the Transitional Care Model (TCM) regarding their content, timeframe, and improvement in older patients with multiple chronic conditions.	Review of systematic literature reviews under PRISMA guidelines between 2011 and 2020.	5 of 62 systematic reviews analyzed.	TCM generates significant improvements in the transitions of older patients from hospital to home, with decreased readmissions and financial costs and increased quality of life and patient satisfaction during discharge.	The transition from hospital to home is a complex process for older patients with multiple chronic conditions. A specific focus on coordination, continuity, and patient education should be implemented throughout the discharge process.<br>Nurses with specialized knowledge in transitional care are needed to ensure safe transitions.<br>The results of the overview indicate that several elements of the Transitional Care Model can achieve significant improvements in older patients' transitions from hospital to home, as all elements of the model cover vital aspects of the discharge process.<br>However, a trend toward a decrease in hospital readmissions and costs was found, as well as an increase in patient quality of life and satisfaction with discharge.<br>The results of the overview indicated significant outcomes for patients when transitional care nurses and multidisciplinary care teams implemented elements of the Transitional Care Model.	Subject Patient<br>Goal: Promote self-management<br>Context: Hospital and home<br>Nursing/Service<br>Nurses with specialized knowledge in transitional care are needed to ensure safe transitions.<br>The Transitional Care Model has elements to support transitions between hospital and home.<br>Multiple elements of the model have achieved improvements.<br>These include:<br>1. Coordination<br>2. Communication<br>3. Collaboration<br>4. Information<br>5. Education<br>6. Pre-established structured follow-ups after discharge<br>Consequences<br>Continuity of care in transitions between hospital and community care.<br>A trend toward a decrease in hospital readmissions and costs, as well as an increase in patient quality of life and satisfaction with discharge.<br>Other considerations<br>Having a model is useful to ensure positive outcomes in the hospital-home transition process.	CASP<br>10/10	1
Breneol et al<br>2017<br>Canada	To identify evidence-based interventions, frameworks, programs, or models to support the hospital-to-home transition for children with medical complexity.	A scoping literature review was conducted using the JBI method, with quality measured using the MMAT, and published any time through 2016.	14 of 2,088 studies were analyzed.	Three main categories of interventions were identified: 1. comprehensive care plans; 2. complex care programs; and 3. integrated delivery models.<br><br>The overall quality of the studies included was moderate.	Evidence-based guidelines to ensure appropriate hospital-to-home transitions for children with medical complexity are lacking.<br>It is imperative to identify potential models to support this transition.<br>There is a need for consensus around the definition of children with medical complexity, and the limited number of these studies and the lack of high-quality evidence indicate the need for further research to improve the hospital-to-home transition and,	The subject is a patient and family with high medical complexity.<br>Goal: Achieving a Successful Transition with Positive Health Outcomes<br>Hospital-Home Context<br><br>Nursing/Service: Ensuring a successful hospital-home transition requires a nursing care coordinator role and the development of integrated solutions for this population that address the needs identified by the family and support positive outcomes across the hospital-	CASP<br>10/10	5

					ultimately, improve outcomes for patients and their families.	home and community-based care continuum.<br>Consequences<br><br>Other Considerations<br><br>A precise definition of the recipients of care during the hospital-home transition is essential.		
Brown et al, 2021<br>Canada	To determine the feasibility of using hospital chart data to measure integrated care processes.	Development of an integrated care transition framework and associated care characteristics with a pilot study to test whether the characteristics could be applied	214 inpatient charts.	Twenty-four characteristics were tested, and fifteen characteristics could be reliably measured through chart review. Of these, the percentage of patients classified as receiving integrated care varied widely across items, from 0.05% to 84.1%.<br>The framework presented in this article can guide measurement of system and clinical delivery of integrated care transitions. In combination with other tools, chart review can provide insight into daily care delivery that would not otherwise be accessible and highlight areas requiring practice changes.	Chart review cannot address the full breadth of integrated care, but it can help understand how care processes are implemented in routine care.<br>Integration is key to improving the quality-of-care transitions for older adults. The list of integrated care characteristics developed here can help systems determine ways to improve the degree of integration occurring within the clinical system and at the individual level, in conjunction with other patient-centered care transition tools	Subject: Patient and Family<br>Goal: Achieve balance between the pressure of discharge from the hospital and the patient's needs to ensure the best clinical outcomes for the patient.<br>Context: Coordination of care between the hospital and the community with available clinical information. Individualized interdisciplinary care. Planning for the disposition of multidisciplinary services follows a care pathway or guide (developed with the discharge risk tool).<br>Nursing/Service:<br>Should involve the patient and ensure basic self-care knowledge. Coordination between professionals and services at the in-hospital level.<br>Specific education or training for everyone between hospital and community health care providers for discharge care. All recommended post-hospital home care is implemented at the time of discharge. An individual's risk is assessed to determine the level of care transition support needed during the hospitalization (using the hospital discharge screening tool). The individual's health and social care needs for discharge are discussed in regular multidisciplinary meetings.<br>Patient involvement in care planning	JBI<br>7/8	6

						The discharge plan is discussed with the family.<br>A written discharge information form is provided to the client.<br>Discharge instructions are jargon-free.<br>Consequences<br><br>Fewer adverse events and fewer hospital readmissions, resulting in greater resource efficiency.<br>Other considerations<br><br>The client has a primary care physician (PCP) who is registered upon admission to the hospital.<br>Medications are guaranteed.<br>Follow-up appointments are scheduled.		
Bucknall et al., 2020<br>Australia	To determine the current evidence on patient and family engagement in communication with healthcare professionals during care transitions to, within, and from acute care settings.	Integrative review	40 studies	Four themes: 1) Collaboration in care: patients and families should be partners in decision-making and care; 2) Increasing communication during transitions: intrinsic and extrinsic factors that support transition communication between patients, families, and healthcare professionals; 3) Barriers to information exchange: difficulties patients and families face in taking an active role in the transition; and 4) Outcomes of communication during transitions: experiences reported by patients, families, and healthcare professionals	While attitudes toward patient and family engagement in transition communication in acute settings are generally positive, current practices vary. Structural supports for practice are not always present. Implications for Practice: Organizational strategies to improve communication must incorporate an understanding of patients' needs. A structured approach that considers time, privacy, location, and appropriateness for patients and families is needed. Communication training for patients, families, and healthcare professionals is required. Healthcare professionals must respect the patient's right to be informed by communicating regularly	Subject: Patient, family member, healthcare professional<br>Goal: Facilitate the hospital-home transition experience for the patient, their family, and healthcare staff<br>Context: Hospital-home transition.<br>Nursing/Service<br>Collaboration in care with participation in decision-making by the patient and family.<br>Increase communication and support to facilitate it during transitions between patients, families, and healthcare professionals.<br>Overcome barriers for patients and families by taking an active role in the transition.<br>Consider the experiences reported by patients, families, and healthcare professionals.<br>Consequences<br>Positive experience with adequate communication<br>Other considerations<br>Patient preferences: Requesting permission prior to procedures; being identified as a person;	CASP 10/10	5

						receiving information about the treatment plan and implementation; warmth, a positive approach, and trust in the relationship; being included in the transition plan; being involved in their own care; receiving advance information about special procedures or transfers; being informed about their health status; explaining the role of team members; receiving detailed explanations about discharge medications.<br><br>Family preferences: ensuring that patients' treatment preferences are respected; being encouraged to participate in transition care to reduce readmissions; warmth and quality in treatment; including the family to support patient safety; receiving information about the patient's health status and how to advocate for the patient and support them with self-care during admission, stay, and discharge; knowing which nurse is in charge of discharge.<br><br>Healthcare professional communications: communicating the care and treatment plan; Provide space to clarify patient concerns; ensure that patients are recognized and respected as individuals with sensitivity to their feelings and fears about discharge; actively and explicitly encourage patient participation by soliciting input and providing clear instructions, particularly when there is a potential for misunderstanding; Include patients in discussions about their care to help them feel more confident and in control; tailor goal-setting and teaching self-management skills to the patient's		

						cognitive and activation abilities; Avoid jargon or acronyms to allow patients to participate equally in the conversation; Involving older adults in transfer conversations and procedures requires ongoing support from healthcare professionals who take into account the patient's ability and willingness to contribute; Explain the role of each participant in the handover to the bedside to avoid disrupting the process; Explain and discuss discharge medications with a patient at a time when the patient is receptive to understanding and retaining the information.		
Chakurian et al 2021<br>USA	To describe two transitional care models based on the Care Coordination Atlas, analyzing their components, dynamics, and impact on hospital readmissions.	2015–2020 integrative review methodology in PubMed and Scopus.	Fourteen studies conducted at 18 skilled nursing facilities and 50 hospitals were used for data extraction and analysis	The Redesigned Discharge model had five components, and the Improved Outcomes by Optimizing Safe Transitions model had eight components across the nine domains of the Care Coordination Atlas. Communication dominated activities in both models, while neither addressed accountability/responsibility. Implementation was influenced by leadership commitment to understanding the complexity of the models, culture change, integration of the models into workflows, and associated labor costs. Model implementation studies consistently reported improvements in facilities past 30-day all-cause hospital readmission rates.	The Care Coordination Atlas was a useful framework to guide the analysis of transitional care models.<br><br>Leadership commitment to and participation in model implementation is vital. Models do not focus beyond the immediate post-discharge period, limiting the impact on chronic disease management. Frameworks such as the Care Coordination Atlas are useful in helping guide the development of care coordination activities and their associations with readmission rates.<br>Care coordination is critical for safe and effective patient transitions across the healthcare continuum and community services.<br><br>The Care Coordination Atlas as a theoretical framework provided a way to compare critical components of the models to identify opportunities for shared measurement and comparison of	Subjects: Patients, healthcare professionals, healthcare system (with its available technology).<br><br>Goal: Reduce readmissions, ensure safe transitions.<br><br>Context: The models do not focus beyond the immediate post-discharge period, which limits the impact on chronic disease management.<br><br>Nursing/Service: It is based on coordination. Leadership commitment to and participation in the implementation of the model are vital.<br><br>It defines nine domains of care coordination activities:<br>1. Establish accountability or negotiating responsibility,<br>2. Communicate,<br>3. Facilitate transitions,<br>4. Assess needs and goals,<br>5. Create a proactive care plan,<br>6. Monitor, follow up, and respond to change,<br>7. Support self-management goals,	CASP 10/10	5

					findings across different settings, providers, and levels of care	8. Connect with community resources,<br>9. Align resources with patient and population needs.<br><br>The Atlas identifies five approaches to support comprehensive care coordination across all populations and settings:<br>1. Coordination-focused teamwork,<br>2. Healthcare facilities,<br>3. Care management,<br>4. Medication management, and<br>5. Health information technology-enabled coordination.<br><br>Consequences<br>Transitional care programs, guided by proven models, are an important component of effective care coordination, and care coordination has been shown to:<br>Reduce fragmentation and costs.<br>Improve the quality of healthcare.<br>Reduce adverse events.<br>Prevent hospital readmissions.<br>Other considerations<br><br>The Care Coordination Atlas is a useful framework to guide the analysis of transitional care models and to help guide the development of care coordination activities and the associations with readmission rates.		
Chen et al, 2021 Australia	Identifying challenges and opportunities for stroke survivors and their caregivers in the transition from hospital to home care.	An integrated systematic review	23 studies	. The main findings were:<br>1) Health and social care systems facilitate or hinder the care of stroke patients and their caregivers. 2) Organizational resources and adequate coordination of services are required. 3) The professional-patient and caregiver relationship is essential for care and support. Patients and caregivers should be assessed to determine their level	The stroke patient and caregiver dyad require support during the hospital-home transition to cope with the challenges. This includes, among other things, strengthening coping and support.<br>Adequate care improves the transition experience for those involved.	Subject: Patient, Caregiver, Healthcare Professional<br>Goal: Strengthen coping skills, support, and the caregiving experience during the transition<br>Context: The hospital, the home, the healthcare system, and the multidisciplinary team<br>Nursing/Service: Educate, demonstrate, facilitate, coordinate, and motivate through the caregiving relationship	CASP 10/10	5

				of readiness to adapt during the transition from hospital to home.		Consequences: Improve coping, support, discernment, and caregiving experience.<br>Other Considerations: A healthy transition allows for a greater sense of control and a more positive outlook: resilience.		
Cho et al., 2015<br>USA	Describe the meal program model to facilitate the hospital-home transition.	Descriptive study of	121 patients	The model was developed to address risk factors commonly associated with preventable hospital readmissions. A total of 18,010 meals were delivered to 121 clients.<br>On average, clients received 6.25 meals per week, and meal delivery began, on average, 8.95 days after discharge.	. The program was well accepted.<br>Hospital readmissions at three and six months decreased compared to typical rates.<br>The risk of malnutrition among people undergoing hospital-home transition was prevented.<br>This program and the delivery of medications complement and facilitate care during the transition.<br>Medical and community services can synergize to promote quality of life among high-risk homebound clients	.<br>Subject: People in transition<br>Goal: Prevent malnutrition in high-risk individuals. Context: interdisciplinary team, institution and health system, community.<br>Nursing/Service: Support in home food delivery.<br>Consequences: Support quality of life and reduce hospital readmissions.<br>Other considerations: prevention generates cost-benefit for health.	JBI 6/6	6
Comin Colet et al., 2016<br>Spain	To describe the stages and characteristics of a model that supports care during care transitions in people with acute and chronic heart failure.	Document Review	N.A.	Early care models should be based on management that responds to the best available evidence.<br>Follow-ups should be planned and structured to promote patient empowerment.<br>There should be coordinated and integrated care to promote continuity of care.<br>The expected effect is to reduce mortality and readmissions and improve quality of life	Integrated care models for heart failure revolve around interventions during care transitions.<br><br>They are multidisciplinary and patient centered.<br>They are designed to ensure continuity of care.<br>They have been shown to reduce potentially avoidable hospital admissions.<br><br>Key components of these models are early intervention during the hospitalization phase,<br>discharge planning, early review and structured follow-up after discharge, advanced transition planning,<br>and the participation of physicians and nurses specializing in heart failure.	Subject: patient, family, caregivers, healthcare team.<br>Goal: Respond to specific needs to support hospital-to-home transitions.<br>Context: Multidisciplinary team; coordinated services; availability of necessary equipment and supplies; availability of required technology.<br>Nursing/Service:<br>1. Patient-centered care<br>2. Comprehensive patient assessment in context<br>3. Integrated patient assessment and intervention<br>4. Promoting patient empowerment by promoting self-care and self-sufficiency<br>5. Interventions based on multidisciplinary teams, through... 6. Systematic use of interventions in patients (planned contact)<br>7. Systematic management based on clinical practice guidelines	JBI 6/6	6

						8. Promotion of new nursing roles to care for, manage, educate, promote self-care, motivate, facilitate, coordinate, monitor, communicate, anticipate, prevent, clarify doubts, and evaluate results.<br>9. Conduct follow-up.<br>Consequences<br>Reduce readmissions<br>Improve health conditions of people with heart failure.<br>Improve quality of life<br>Other considerations<br>The levels of clinical complexity must be considered when scheduling follow-up.		
Da Costa et al 2020<br>Brazil	To assess the conformity of nursing care with best evidence in the transition of care for older adults admitted to hospital to home.	This best evidence implementatio n project, based on the Joanna Briggs Institute model, was conducted at a university hospital with older adults, caregivers, and nurses between July and August 2019.	Eight evidence-based criteria were audited through interviews, medical records, and a computer system	At baseline, a lack of knowledge and ongoing updating regarding the transition of care and hospital discharge plan was observed. Educational programs contributed to reducing barriers. By applying the best available evidence to the transition, there was an improvement in compliance with all the evaluated criteria:<br>1. People received education about the transition.<br>2. Patients and caregivers were educated about interventions and self-management.<br>3. People had an individualized care plan for their hospital-home transition.<br>4. Patients and caregivers were involved in developing their care plan for the transition.<br>5. Coordinated service delivery between the hospital and the home or community.<br>6. Care needs communicated from the hospital to community care providers.<br>7. Post-discharge follow-up.<br>8. Multifaceted approaches to caring for people during the transition.	Audited evidence-based criteria showed an increase in compliance rates for the strategies implemented, contributing to improved transitions of care for older adults.	Subject: Patients, caregivers, family members, nurse<br>Goal: Achieving a successful transition<br>Context includes the patient, their caregiver, the healthcare team, the hospital, the home, and the community. During transitional care, clients/individuals must feel situated, that is, an integral part of the context in which they are located.<br>The transition must begin upon hospitalization.<br>Nursing/Service<br>Education, guidance, outreach to patients to guide, educate, and facilitate them. Strengthen patient self-management.<br>Involve patients and caregivers in their transition plan and management. Monitor, evaluate, and inform.<br>Involve caregivers in care by preparing them for discharge alongside patients. Involve them in decisions.<br>It is important to evaluate with performance indicators.<br>Encourage post-discharge communication; increase treatment adherence.	JBI 8/8	6

						create personalized care plans based on patients' needs; post-discharge follow-up with patients through home visits and phone calls; and train the primary care team.<br>Consequences<br>Reduced length of stay and decreased readmissions. Reduced costs.<br>Other considerations<br>Transition involves creating new meanings and perceptions and reframing meanings in relation to the environment. This is a dynamic state<br>that leads us to the notion of adaptation to the environment, and<br>subsequently to the development of trust, which occurs when making the transition, demonstrating deeper knowledge and greater understanding of essential and critical aspects.<br>Discharge planning is essential. Discharge protocols and discharge management plans are useful for structuring the work of multidisciplinary teams in discharge planning.		
De Regge et al, 2017<br>Belgium	To describe in a general and comprehensive manner the role of hospitals by synthesizing the advantages and disadvantages of hospital intervention in chronic care for patients with chronic illnesses found in published	Systematic literature review	32 articles.	Overall, the quality of the studies is high. Four major themes were identified: 1) the impact of hospital-initiated transitional care interventions; 2) the role of specialized care settings; 3) the comparison of inpatient and outpatient care; and 4) the effect of chronic care coordination on patient experience.	Hospitals can play an important role in transitional care interventions and chronic care coordination with improved patient outcomes by taking a leadership role in integrated care programs. Furthermore, patient experiences are positively influenced by the coordinating role of a specialist.<br>Specialized care settings, as components of the hospital, facilitate the coordination of care processes. Hospitals play an important role in transitional care interventions and care coordination.	Subject Patient Nurse healthcare team Hospital other care facilities Goal Improve care during transition by ensuring continuity.<br>Context Hospital Community Living site Primary care facilities Nursing/Service Includes clinical follow-up and integrated care.<br>Should include case management and coordination with the patient care team.<br>Consequences Maintain health; decrease readmissions and lower costs.<br>Improve patient experience<br>Other considerations Hospitals play an important role in	CASP 8/10	5

	empirical studies.					coordinating transition processes that needs to be clarified.		
Desai et al., 2015 USA	The objective of this study was to conduct a targeted literature review of studies examining the effectiveness of family-centered transition processes from the hospital and ED to home in improving patient health outcomes and healthcare utilization.	A systematic search	16 randomized clinical trials conducted..	Family-tailored discharge education is associated with improved patient health outcomes<br>An assessment of transition needs or the provision of an individualized transition record is associated with improved patient health outcomes and reductions in healthcare utilization.<br>The effectiveness of post-discharge telephone follow-up and/or home visits on healthcare utilization showed mixed results.	Patient-tailored discharge education is associated with improved health outcomes in pediatric patients.<br>Improving the quality-of-care transitions from hospital to home or the ED reduces unnecessary healthcare utilization and ensures that patients and families are better prepared and have the necessary resources to continue managing their child's healthcare at home.<br>Family-centered education at discharge appears to be associated with better outcomes in pediatric patients during ED-to-home transitions, but the hospital-home transition needs further study.<br>An individualized plan is associated with better outcomes in adult patients and needs further study in children.	Subject: Patient, Family, Healthcare Team<br>Goal: To achieve the best healthcare outcomes and optimal healthcare utilization.<br>Context: Hospital, Home<br>Nursing/Service: Assess and plan individually, including the family. Coordinate with the team, inform, communicate, schedule, record, and follow up.<br>Consequences: Better clinical outcomes, lower unnecessary healthcare costs, and a more positive patient and family experience.<br>Other considerations<br>It also includes other outcomes such as satisfaction, level of discharge readiness, functional and health-related quality of life assessment, absence from routine roles, level of knowledge of the care plan, level of adherence to follow-up and treatment, and completeness of outpatient workup.<br>Regarding the use of external indicators, these include 30-day emergency room use, readmission, and costs.	CASP 10/10	5
Dias da Silva et al., 2018 Brazil & y Canada	To identify the available literature on care transition strategies between healthcare levels in Latin American countries.	This integrative review included studies indexed in the PubMed, LILACS, Web of Science Core Collection, CINAHL, Scopus, and Scientific Electronic Library Online	Eleven articles	Hospital-home transition strategies are grouped into:<br>1. Discharge planning.<br>2. Advance care planning.<br>3. Patient education and promotion of self-management.<br>4. Medication safety.<br>5. Comprehensive communication of information.<br>6. Outpatient follow-up.	Nursing plays a leadership role within the team during the hospital-home transition.<br>Transition activities begin close to the patient's discharge. It is suggested that they begin from the moment of admission.	Subject: Patients<br>Goal: Promote and protect the health of patients undergoing the hospital-home transition.<br>Hospital - Home Context<br>Nursing/Service: Nurses are members of the multidisciplinary team with leadership roles. They must plan, anticipate, educate, ensure medication safety, communicate, and provide support or follow-up.<br><br>Consequences	CASP 10/10	5

		(SciELO) databases, published in Portuguese, Spanish, or English between 2010 and 2017.				Health care must be integrated.<br>Results must impact on patients, providers, and health systems.<br>Other Considerations<br>The concepts need to be developed in the Latin American region.<br>Evidence is lacking to determine the effectiveness of the strategies.		
Eliassen et al., 2020<br>Norway	To identify how rehabilitation models that support the hospital-to-home transition for patients with acquired brain injury (ABI) are described in the scientific literature and analyze the content of these models.	Scoping review with different designs.		Five categories were identified:<br>1. Home-based multidisciplinary teams<br>2. Key coordinators<br>3. Trained family caregivers or lay health professionals<br>4. Pre-discharge planning<br>5. Self-management programs<br><br>Comprehensive professional and contextual descriptions are lacking.	There is a wide variety of rehabilitation models that support the hospital-to-home transition phase for people with ABI. This variety may indicate a lack of consensus on best practices. However, it may also reflect contextual adaptations.<br><br>This study indicates that health services research lacks robust and comprehensive descriptions of contextual characteristics, which may limit feasibility and transferability to diverse contexts.	Subject: Patient, formal caregiver or family member, multidisciplinary team<br>Goal: Meets patient expectations and seeks to facilitate their return to the community.<br>Context: Hospital-Home Transition<br>Nursing/Service: Coordinate, plan, support self-management.<br>Consequences:<br>There is no global consensus on how to organize services upon return home. Economics, geography, and culture are factors. Models have limitations for transferability.<br>Other Considerations:<br>Models include home multidisciplinary teams; key coordinators; training of skilled family caregivers or non-professional health professionals; pre-discharge planning; and self-management programs.<br>There is a lack of continuity of care during the Hospital-Home transition.<br>Standardization of service offerings is necessary.<br>The model must be applied in context.	CASP 9/10	5
Feil et al. 2017<br>Brazil	To identify nursing activities in the transition from hospital to home care, considering	An integrative review	22 articles out of 2,279 articles	The results indicated five thematic categories with the main activities of nurses in the transition of care: discharge care planning; assistance with social rehabilitation; health education;	There is a need to improve nursing care practices and the organization of nursing activities to promote care coordination, with an emphasis on the transition from hospital to home.	Subjects: patients, family members and caregivers, former caregivers, and those who provided care to the patient at home.	CASP 10/10	5

	evidence from the literature.			coordination with other services; and support after discharge.<br><br>Category 1 – Discharge Care Planning<br>Discharge planning is carried out jointly by hospital nurses, the patient and family members, or with the multidisciplinary team, and is revised during the hospital stay, according to the patient's medical and psychosocial changes. Discharge plans contained information on previous diagnoses, medication administration, medical history, assessment of psychosocial conditions, financial and housing conditions, and follow-up care after hospital discharge. The plan was given to the patient at the time of hospital discharge.<br><br>Category 2 – Social Rehabilitation Assistance<br>These activities aim to enable patients to resume their daily activities after hospital discharge. They focus on interaction between family and the community, promoting positive attitudes, acceptance and adaptation of the disease to daily living activities, mitigating feelings of abandonment, and fostering favorable rehabilitation expectations. Nurses assessed aspects that could improve or hinder the patient's recovery, such as the physical, social, psychological, and motivational conditions of the patient and their family.<br><br>Category 3 – Health Education<br>Nurses carry out health education activities in a variety of ways: dietary changes and possible food restrictions, physical exercise,	The findings of this study allowed us to identify the main care transition activities performed by nurses.<br><br>This study provides useful information for improving care practices and the organization of nursing activities to promote care coordination during hospital discharges, with a focus on care transitions.<br>Aspects related to hospital discharge preparation, health education for patients and families, support for continuity of care at home, and post-discharge follow-up are indicators for assessing the quality of care provided in health services. This study provides useful information for improving care practices and the organization of nursing activities to promote care coordination during hospital discharges, with special attention to care transitions. According to the results obtained, there are few studies on nurses' routine activities during care transitions. More studies on this topic are needed in Brazil, as care transition is a strategy that can contribute to the implementation of integrated health systems.	Goal: Positively impact quality of life, avoid unnecessary hospitalizations, and reduce healthcare costs.<br>Context: Different care settings within the same institution or between this institution and other care settings.<br>Nursing/Service: Discharge care planning, Social rehabilitation assistance, Health education, Coordination with other services, Post-discharge follow-up.<br><br>Consequences: Efficient and safe transitions<br>Other considerations: Nursing actions, along with the interdisciplinary team, should be aimed at ensuring coordination and continuity of care as the transition between different settings occurs, where it is necessary to overcome fragmentation of care and ensure ongoing care for patients.		

				correct medication use, including dosage, frequency of administration, and schedules, interactions with continuously used medications, recognition of signs and symptoms of the current illness, and self-care at home. Some nurses perform medication reconciliation, assessing the effects of medications used before hospitalization and medications prescribed in the hospital setting. Others use informational support leaflets to reinforce care instructions and simple, concise language, as well as feedback to check the patient's understanding of the information provided.<br><br>Category 4 – Coordination with Other Services<br>This activity was described in three sample studies. Nurses communicate information about the patient's discharge plan to primary care teams, managing the transition of care between different points in the healthcare network. This coordination between services can be achieved through notifications from hospital nurses about the patient's discharge to the referring service.<br><br>It can also be achieved through telephone calls through which hospital nurses advise primary care nurses to visit patients at home and clarify their concerns. The hospital's computerized system interconnected with the local health network is also used. The discharge plan is sent electronically to the primary health care service and is used by the care team to monitor home care.				

				Category 5 – Post-Discharge Follow-Up<br>Post-discharge care follow-up activities were described in the first 16 articles. Telephone calls or home visits were conducted to assess the discharge plan and address the instructions provided in the hospital setting, as well as clarify any questions regarding this information. The main points are identifying and providing guidance on treatment aspects, warning signs and symptoms, medication administration, and verifying the patient's understanding of self-management activities. Clarification of appropriate locations for care and follow-up consultations was provided. Telephone contacts made it possible to identify patient and caregiver questions regarding home care, and to designate home visits for patients at risk for rapid intervention during the phone call.				
Green et al., 2025 Australia	The objectives were (i) to explore existing knowledge and practice in Australia regarding transitions of maternal postnatal care between hospital and primary care and (ii) to understand the factors that facilitate and hinder the implementatio	A scoping review was conducted according to the PRISMA-ScR guidelines. Medline, Embase, CINAHL, Scopus, and The Cochrane Library were searched using MeSH terms, subject headings, and keywords. Full-text articles in	Eighteen studies were included.	The transition of maternal postnatal care between hospital and primary care varied. The critical components of care that were valued by women and health professionals and that promoted effective care transitions were grouped into four concepts: "Woman-Centered Discharge Planning and Process," "Integrated Care," "Follow-Up Care," and "Continuity of Care." Discharge communication in Australian health services is diverse. Women and health professionals require clear discharge communication that highlights complications, guides follow-up, and promotes continuity.	Australian postnatal transition between hospital and primary care is inconsistent and ineffective.<br>The lack of robust handover between services hinders evidence-based follow-up care after postnatal discharge from hospital, particularly after pregnancy complications.	Subject: Mothers, children, family, health professionals<br>Goal: Promote an effective transition with positive effects on current and future health. Prevent long-term risks.<br>Hospital-Home Transition Context<br>Nursing/Service: Discharge planning centered on the woman and her newborn, comprehensive care, follow-up, and ongoing care. These include a collaborative, woman-centered approach to discharge planning and diagnosis-specific discharge summaries that provide clear guidance for appropriate follow-up after pregnancy.	CASP 8/10	5

	n of optimal postnatal discharge and the transfer of care from maternity to primary care.	English were included from January 1, 2010, to June 8, 2024.				Consequences: The lack of robust transfer of care between services hampers evidence-based follow-up care after postnatal hospital discharge, particularly for women experiencing pregnancy complications.<br>Other Considerations:<br>Effective discharge continuity and communication mechanisms that meet the needs of primary care providers are required. Universal processes, supported by a broader redesign of healthcare infrastructure, are urgently needed to address this barrier to optimal postnatal and long-term preventive healthcare.<br>Other Considerations:<br>The attributes of the care concepts during the patient-centered transition process: follow-up care, integrated care, and continuity of care are described.<br>CASP 8/10 5		
Groenvynck et al, 2021<br><br>Netherlands & Belgium	This study, conducted within the framework of the European research consortium TRANS-SENIOR, proposes a model to optimize the transition from home to a nursing home, based on the experiences of older adults and informal caregivers.	A review of relevant literature was obtained from the CINAHL and PubMed databases. Studies were included in which older adults or informal caregivers identified experiences, needs, barriers, or facilitators during the transition from home to a nursing home.	Twenty articles were included	The data extracted from the included studies were assigned to the different stages of the transition (pre-transition, mid-transition, and post-transition), thus creating the TRANSCIT model. Finally, the results were discussed with a panel of experts, leading to the final proposal of the TRANSCIT model.	The TRANSCIT model identified that older adults, and informal caregivers expressed a general need for collaboration during the transition from home to a nursing home.<br><br>She identified four key components along the transition trajectory (i.e., pre-transition, mid-transition, and post-transition):<br><br>(1) support,<br>(2) communication,<br>(3) information, and<br>(4) time.<br><br>The TRANSCIT model could advise policymakers, practitioners, and researchers in the development and evaluation of future transitional care interventions.	Subject: Patient, informal caregivers, and healthcare providers<br>Goal: Ensure coordination and continuity of care to optimize the process.<br>Context: Transition is divided into three phases: pre-transition, mid-transition, and post-transition.<br><br>Nursing/Service: Information, communication, and support must be guaranteed, and the time required for each stage of the process must be anticipated and guaranteed. Consequences: The model allows for the creation of a partnership to ensure care during the transition.<br>Other considerations<br><br>The transition from home to a nursing home can be stressful and	CASP 10/10	5

					It can serve as a guide for assessing the needs of older adults and their informal caregivers, emphasizing the need for collaboration, thereby reducing fragmentation in transitional care and optimizing the transition from home to a nursing home.	traumatic for both older adults and informal caregivers and is often associated with negative outcomes. Furthermore, transitional care interventions often lack a comprehensive approach, which can lead to fragmented care. To avoid this fragmentation and optimize transitional care, a comprehensive and theoretical model is essential. This must include the needs of both older adults and informal caregivers.<br><br>Monitoring and strengthening autonomy are part of the outcomes of an adequate process.		
Hestevik et al 2019<br>Norway	To systematically summarize qualitative findings describing older adults' experiences adapting to everyday life at home after hospital discharge.	A meta-summary of the qualitative findings was used using the Sandelowski and Barroso method	13 studies.	The following categories emerged:<br>(1) Experiencing an uncertain and insecure transition,<br>(2) Adapting to a new situation at home,<br>(3) What would they do without me as their informal caregiver?<br>(4) Experience of a paternalistic medical model.	Understanding older adults' experiences with their own care is crucial for identifying and addressing problems related to the transition from hospital to home and can help reduce impairments and facilitate more satisfactory healthcare.<br><br>The results emphasize the importance of assessment and planning, information and education, preparation of the home environment, involvement of the older adult and their caregivers, and support for self-management in the discharge and home follow-up processes.	Subject: Patient, informal caregiver, healthcare personnel<br>Goal: Positive clinical outcomes decreased risk of readmission.<br>Context: Hospital-Home Transition<br>Nursing/Service: Optimize the hospital-home transition experience for the patient and their informal caregiver.<br>Consequences<br>A key challenge in transitional care is providing medical care tailored to the needs of older adults, as perceived by them rather than as defined by healthcare professionals.<br>Challenges remain in improving older adults' satisfaction with the healthcare services included in transitional care.<br>Other considerations:<br>Hospitalization can reduce older adults' ability to manage their home life after hospital discharge.<br>Inadequate discharge practices can be associated with adverse outcomes and an increased risk of readmission.<br>Better communication between older adults, hospital	CASP 10/10	5

						professionals, and home care professionals is needed to improve care coordination and facilitate recovery at home.<br>Organizational structures may need to be redefined and reorganized to ensure continuity of care and the well-being of older adults during transitional care situations.		
Hewner et al., 2021<br>USA	To explore the literature on service integration and coordination for high-need, high-cost (HNHC) patients to answer the following questions: What transitional care models are used for the management of HNHC patients in the United States? And how effective are they in reducing low-value utilization and improving continuity?	A scoping review using a stepwise approach guided by JBI and PRISMA guidelines	Nineteen studies	The studies focused on Medicaid, Medicare, dual-eligible patients, veterans, and the uninsured or underinsured. High-need, high-cost patients were identified based on previous patterns of inpatient and ED visit utilization, high cost, multiple chronic medical diagnoses, or a combination of these factors. The tools used to identify these patients included the Hierarchical Condition Category Predictive Model, the Older Adult Risk Assessment, and the 4-Year Prognostic Index score. Most studies combined features of multiple case management models with varying levels of impact.	Care coordination and case management were the primary strategies used to address the care needs of HNHC patients.<br>Interventions should reflect a strategy to efficiently identify and direct HNHC patients to the most appropriate resources.<br>The full potential of current technological offerings in the science of care coordination has not been realized.<br>Care management interventions must evolve to connect multiple healthcare settings and community organizations through communication and collaboration.<br>Continuity of care is vital during the immediate post-discharge period. However, monitoring continuity as an outcome remains poorly defined and does not reflect actual practice.	Subject: Patient, family, healthcare providers<br>Goal: Guide the transition intervention, usually in combination with multidisciplinary team-based care.<br>Hospital-Home Transition Context<br>Nursing/Service: a comprehensive discharge needs assessment, comprehensive education on illness self-management and behavior change, behavioral health interventions, increased contacts, medication review and management, increased access to community resources, and quality outcome monitoring.<br><br>Other Considerations<br>Regardless of the model employed, studies implemented complex interventions that utilized multiple components. Most studies used an identified individual (APRN, RN, social worker, or navigator) to guide the transition intervention, usually in combination with multidisciplinary team care.<br><br>The use of the following care management models is reported:<br>IBH = integrated behavioral health and primary care.<br>ECM = integrated care management.	CASP 8/10	5

						CCM = centralized care management.<br>TCM = transitional care management.		
Hirschman et al.<br>2015<br>USA	Summarize the evidence base for the MTC (Transitional Care Model), its 9 components, and their measurement and impact.	Presentation of the Model and its evidence.	Applicable to the adult population over 65 years of age with chronic health conditions during the hospital-to-home transition.	MTC is a nurse-led intervention for older adults at risk of poor outcomes as they move between healthcare settings and between physicians.	MTC has 9 components: 1) Assessment of adults transitioning from hospital to home at high risk of adverse outcomes. 2) Staffing.<br>Utilizes Nurse Practitioner Nurses (APRNs) who assume primary responsibility for care management during episodes of acute illness. 3) Relationship Management. This establishes and maintains a trusting relationship with the patient and family caregivers involved in the patient's care.<br>Patient and Caregiver Engagement.<br>4) Involves older adults in the design and implementation of a care plan aligned with their preferences, values, and goals. 5) Risk and symptom assessment/management<br>Identifies and addresses the patient's priority risk factors and symptoms. 6) Education/self-care promotion prepares older adults and family caregivers to quickly identify and respond to worsening symptoms. 7) Collaboration<br>Promotes consensus on the care plan among older adults and members of the care team. 8) Promotion of continuity prevents interruptions in care from the hospital to the home by having the same clinician participate in all these facilities.<br>And 9) Promotion of coordination promotes communication and connections between health professionals and community professionals.	Subjects: older adults, family caregivers, and health care personnel.<br>Goal: Maintain maximum functionality and clinical stability to improve quality of life while reducing care burden.<br>Context: Transition from hospital to home, institutions, community.<br>Nursing/Service: Assessment, coordination, ensuring available resources, appropriate communication, promoting autonomy and participation in care plans, evaluation, and collaboration to facilitate expected outcomes.<br>Consequences: Measuring changes over time in key outcome areas, such as patient symptoms, functional status (e.g., cognitive, physical, emotional), and quality of life, as well as family caregiver outcomes, such as burden, is important for comparing evidence of the impact of TCM and continuously improving its performance (see Table 2).<br>Evaluating the perspectives of patients, family caregivers, and collaborating clinicians on the model (e.g., overall experience with care and specific dimensions such as continuity of care) provides important additional dimensions of the perceived value of TCM. Changes in resource use (e.g., time to first readmission, total number of all-cause hospitalizations, total number of hospital days) are critical to fostering support for investment in MTC. Both program implementation costs (e.g., time spent by nurse practitioners	JBI<br>8/8	2

					In three randomized controlled trials, it has been shown to reduce costs, complications, and rehospitalization.	implementing transitional care services) and avoided costs (e.g., number of ED visits or readmissions avoided) are valuable data for demonstrating the effects of MTC.<br>Other Considerations<br>The components of MTC are:<br>1. Screening<br>This is intended for adults transitioning from hospital to home and who are at high risk for poor outcomes.<br>2. Available Staffing<br>This utilizes APRNs who assume primary responsibility for managing care during episodes of acute illness.<br>3. Maintaining Relationships<br>This establishes and maintains a trusting relationship with the patient and family caregivers involved in the patient's care.<br>4. Involve Patients and Caregivers<br>Involve older adults in the design and implementation of the care plan aligned with their preferences, values, and goals.<br>5. Risk and Symptom Assessment/Management<br>Identify and address the patient's priority risk factors and symptoms.<br>6. Educate/Promote Self-Management<br>Prepare older adults and family caregivers to quickly identify and respond to worsening symptoms.<br>7. Collaborate<br>Promote consensus on the care plan among older adults and care team members.<br>8. Promote Continuity<br>Avoid gaps in care from the hospital to the home by having the patient present.		
Jones et al., 2022<br>USA	To collect data on patients' care transition	This was an exploratory descriptive	1257 people were interviewed	Eleven percent of patients reported not receiving a phone number to call for post-discharge	An examination of the experiences of patients discharged from a hospital or	Subject: Patients and Caregivers	MMAT 5/5	6

	experiences and factors associated with follow-up appointment completion.	study based on a survey of participants in a collaborative quality initiative.		questions. Nearly 80% of patients (977 patients) received a follow-up phone call, and most found it valuable. Twenty percent of patients (255 patients) reported at least one social determinant of health concern. Lack of transportation was associated with a lower likelihood of completing a follow-up visit, decreasing the odds of completing follow-up by nearly 70% (odds ratio [OR], 0.31; 95% CI, 0.18–0.53; P < .001). Black patients were less likely to report completing a postdischarge follow-up visit (OR, 0.49; 95% CI, 0.36-0.67; P < .001) or receiving prescribed medical equipment (OR, 4.23; 95% CI, 1.30-13.83; P = .02).	medical facility identified inconsistencies in care transition processes, social determinants of health issues that need to be addressed after discharge, and racial disparities among patients attending follow-up appointments.<br><br>These findings negatively impact patients' experience during the transition.	Goal: Improve the experience of patients and caregivers during the hospital-home transition.<br>Context: Hospital-home transition.<br>Nursing/Service Discharge Service<br>Consequences: Understanding the patient's perspective on their care transition process from the hospital or skilled nursing facility (SNF) to home can highlight gaps in care and inform system improvements.<br>Other Considerations: The transition process is variable and significantly influenced by social determinants.<br>Health systems seek to optimize care by improving the patient experience and reducing costs. A key aspect is the post-hospital care transition, which includes education, medication, follow-up, and home care. Common problems include confusion, lack of resources, and poor communication, leading to hospital readmissions. Studies seek to improve this transition, including the patient's perspective and factors such as social determinants of health and racial disparities.		
Kast et al., 2021 Germany	To verify the scientific evidence of the cost-effectiveness of the American hospital-home transition model (TCM).	Systematic review in six databases over a 26-year period	. 3 studies.	The economic effect of TCM changes over time. The costs of a care coordinator could not be determined because little detailed information was provided.<br>TCM may have negative consequences for hospitals.<br>The results are not transferable to Germany.	There is no scientific evidence of the cost-effectiveness of TCM. The optimal duration of this model needs to be clarified based on a detailed description with units and prices and additional consideration of the hospital's perspective. A full economic analysis is needed under German conditions or for similar European countries.	Subject: Patient and caregiver<br>Goal: Reduce readmission and care costs<br>Context: Hospital-home transition in different settings<br>Nursing/Service: Advanced practice registered nurse who assumes primary responsibility for care management during episodes of acute illness.<br>Coordination, communication, and connections between healthcare professionals and community professionals are encouraged.	CASP 9/10	5

						The goal is to promote seamless continuity of care from the hospital to the home by having the same clinical professional involved in all these centers.<br>Educates and promotes self-management.<br>Involves patients and caregivers.<br>Articulates the care plan between older adults and members of the care team.<br>Maintains relationships.<br>Evaluates risk and symptom management.<br>Consequences: Reduce unnecessary complications and costs.<br>Other considerations:<br>To evaluate the hospital-home transition, it is important to consider:<br>Cost-effectiveness:<br>Mortality rates<br>Functionality<br>Patient satisfaction during the period and at three months<br>Number of readmissions<br>Length of hospital stay<br>New health problems requiring hospitalization<br>Resources expended<br>Financial results: costs per patient<br>Average number of total and scheduled visits per patient<br>Emergency room visits<br>Number of phone calls<br>Total cost per patient/time<br>Readmission costs<br>Direct program costs by amount of visits/time.		
Kim et al 2023 Korea	To describe and select the best indicators reported in the literature for improving the quality of transitional care services	A scoping review was conducted to derive core concepts and materials/evidence for transitional care services.	Ten articles were collected from 3,961 participants Three rounds of Delphi studies were conducted	The components of the transitional care service were derived (assessment of the need for the transitional care service period, multiprofessional team, connection to community resources, etc.). The themes for the outcome indicators were: effectiveness, patient safety,	This study suggests practical implications for the service with high relevance and need for the transition period. It also presented outcome indicators for the transitional care service to contribute to an improvement in the quality of care.	Subject: Patient, family caregiver, healthcare team.<br>Goal: To provide an effective and safe hospital-home transition with patient-centered care that improves functionality and health, and strengthens the support network, thereby	CASP 10/10	5

	for discharged patients.	For the derived elements of the transitional care service and assessment indicators a three-round Delphi study was conducted with experts in the fields of healthcare/medicine/nursing/social welfare.,	with 29 participants..	patient-centeredness. When classifying the elements for assessment according to the hospitalization and transition period, the transitional care service elements during the hospitalization period were: the presence/absence of a caregiver and the need for a caregiver, activities of daily living, and the need for home care services. Regarding patient safety, the risk of falls and fractures during hospitalization and the need for medication reconciliation were identified as elements of high importance. For the transition period, the following high-priority elements were derived: the provision of education on appropriate emergency response and management, the provision of information and guidance on service implementation for beneficiaries of the basic livelihood security program, and education for patients' self-management skills. Third, for the derivation of outcome indicators for the transitional care service, in the "effectiveness" category, experts rated a reduction in the 30-day readmission rate as highly important, along with a decrease in emergency department visits and a reduction in avoidable admissions as highly relevant indicators.<br>In terms of "patient safety," a decrease in adverse drug reactions and a reduction in the incidence of falls and pressure ulcers were identified as high-priority indicators. Finally, for the "patient-centeredness" category, the evaluation of the patient experience, the level of satisfaction with the service reported by the dyad, and the		improving overall quality of life.<br>Reduce the burden on caregivers.<br>Phased hospital-home transition, including the hospitalization period, transfer, and home stay.<br>Nursing/Service: To provide a service centered on the patient and their caregiver, with high safety standards. This includes assessment, coordination, support, education, and motivation.<br>Consequences: To improve the quality of life of the dyad.<br>Other Considerations<br>Indicators for Evaluating the Quality of Care in Transition<br>1. Effectiveness<br>Reduced mortality rate<br>Reduced 30-day readmission rate<br>Reduced ED visits<br>Reduced length of hospital stay<br>Reduced avoidable admissions<br>Reduced medical expenses<br>Reduced delayed diagnosis of diseases<br>Improved patient self-efficacy<br>Improved ability to use disease information<br>Improved skills to respond to/manage treatment and disease-related adverse events<br>2. Patient Safety<br>Reduced incidence of falls and incidence/aggravation of pressure ulcers<br>Reduced adverse drug reactions<br>3. Patient-Centered Care<br>Assessment of the patient experience for post-discharge care<br>Reported level of satisfaction with the service provided (SSP)<br>Reported level of satisfaction with the service provided (SSP)<br>Caregivers<br>Reduced caregiver burden<br>4. Related to functionality and health<br>Improved frailty index		

				reduction in the burden on caregivers were identified as high-priority indicators.		Improved activities		
Le Berre et al., 2017<br>Canada	To determine the effectiveness of interventions targeting hospital-to-primary care transitions for older patients with chronic conditions.	Systematic review of randomized controlled trials.	92 of 10,234 studies	. Compared with usual care, significantly better outcomes were observed: lower mortality at 3 months (RD: 0.02 [0.05, 0.00]; NNT: 50), 6, 12, and 18 months after discharge, lower emergency department visit rate at 3 months (RD: 0.08 [0.15, 0.01]; NNT: 13), lower readmission rate at 3 months (RD: 0.08 [0.14, 0.03]; NNT: 7), 6, 12, and 18 months, and shorter mean days of readmission at 3 months (MD: 1.33 [2.15, 0.52]), 6, 12, and 18 months. No significant differences were observed in quality of life	In conclusion, interventions aimed at improving the hospital-home transition are beneficial for older patients and should be included in the reorganization of health services.	Subject: Patient and Healthcare Professionals<br>Target: The interventions seek coordination and continuity of care; structured and pre-established follow-up after discharge, with at least one follow-up beginning within 30 days of discharge.<br>Context: Hospital-home Nursing/Service. Transitional care interventions include: Self-management education, discharge planning, structured follow-up, and coordination among different healthcare professionals.<br>Consequences:<br>Lower mortality rates, emergency department visits, readmissions, and mean days of readmission. No significant differences in quality of life were observed.<br>Other considerations:<br>Transitions from hospital care to primary care for older patients with chronic diseases (CD) are complex and lead to increased mortality and service use.	PRISMA 27/27	1
Lee et al 2017<br>Korea	To systematically review the content and effects of nurse-led transitional care programs for patients discharged from hospital to home	This systematic review with meta-analysis of randomized controlled trials published between 2005 and 2015 was conducted in PubMed, Embase, Cochrane (Central Register of Controlled	Nine studies	Studies included patient assessment, education, and discharge planning in the pre-discharge phase. Nurses performed referral, communication, and care planning in the transition phase. Home and telephone visits, follow-up, and multidisciplinary counseling were included in the post-discharge phase. Several outcome measures, such as hospital utilization (30-day readmission and emergency department visit), quality of life, and cost, were used to identify the effectiveness of nurse-led	The results indicated that the nurse-led transitional care program is effective in reducing unnecessary hospital utilization. However, the small sample size and risk of performance bias are limitations of this study. Therefore, we suggest that well-designed randomized controlled trials should be conducted.	Subject: Patient<br>Goal: The program is designed to help adults and older patients with a variety of medical conditions, reduce ED readmissions, improve quality of life, and reduce unnecessary costs.<br>Hospital and Home Context.<br>Nursing/Service: The service is led by nurses. It is classified into three stages with different activities:<br>Pre-discharge: assessment, education, discharge planning, coordination with the multidisciplinary team	PRISMA 27/27	1

		Trials), and CINAHL		transitional care programs. 30 days<br>Readmission (OR = .73, 95% CI 0.54, 0.98; p = .03) and emergency department visits (OR = .67, 95% CI 0.50, 0.88; p = .005) were statistically significant in the meta-analysis. However, participant blinding was not performed in seven studies, posing a risk of performance bias.		Transition – Referral: strengthening communication and preparing a care plan<br>Post-discharge: conducting a home visit, follow-up phone call, outpatient consultation, assessment, care coordination, education, monitoring, and counseling with the multidisciplinary team<br>During the hospital-home transition, nurses:<br>1. Identified the general characteristics of the patient and the characteristics of the intervention.<br>2. Confirmed the effectiveness of these programs in reducing the use of unnecessary health services.<br>3. Assessed the patient's needs from the moment of hospital admission and upon discharge.<br>4. Their intervention establishes a plan and connects each patient with appropriate resources.<br>5. Sees the need to standardize indicators and measurement tools to determine the program's effectiveness.<br>6. They performed referral, communication, and care planning during the transition phase.<br>7. They followed up with home and telephone visits in coordination with the multidisciplinary team during the post-discharge phase.<br><br>Consequences: A transition program needs to be developed to promote satisfaction and, in addition,<br>Standardized outcome indicators and measurement tools to determine the program's effectiveness.		

						Other considerations: Several outcome measures were used: hospital utilization (30-day readmission and ED visit); quality of life; cost.		
Leithaus et al 2022<br>Belgium	This review summarizes the use of integrated care components to address hospital-to-home transition care, provides an overview of reported outcomes, and describes the impact of the identified components on hospital readmission and emergency department visit outcomes	This study is part of the European TRANS-SENIOR project. PubMed, CINAHL, and Embase were searched for studies in English, German, and Dutch that described an ECM for frail older patients that included pre- and post-discharge components	17 transition model studies.	Fifteen ECMs were reported. All ECMs describe a person-centered, personalized, proactive, and continuous transition care service. Components such as a small care team, intensive follow-up, shared decision-making, and informal caregiver involvement are likely associated with a reduction in hospital readmissions and emergency department visits. Twenty-seven transition care outcomes were reported: 19 service outcomes, six patient outcomes, and two provider outcomes.	In conclusion, the findings of this systematic review suggest that a multi-component MCT, including pre-discharge follow-up and intensive post-discharge follow-up, can reduce hospital readmissions and emergency department visits. Components such as shared decision-making, the involvement of an informal caregiver, and a small, personalized care team with a designated coordinator may increase the success of MCTs. However, the heterogeneity detected in MCTs, as well as poor reporting at the meso- and macro-levels, and the lack of detail in the micro-level reporting of the SELFIE framework, prevent generalized conclusions from being drawn. Future research should focus on robust methodological designs that provide detailed reporting of MCT components and more rigorous and comprehensive process evaluations to understand the impact of each component within these complex multi-level interventions and thus define clear recommendations for practice. Furthermore, deficiencies in patient and healthcare professional outcome measurements have been identified, which require a more robust approach in future research.	Subject: Older adult, caregiver, healthcare provider.<br>Goal: Reduce hospital readmission, mortality, emergency department visits, length of stay, total costs of the intervention, patient health-related quality of life, caregiver burden, and help improve the quality of service for providers.<br>Context: Hospital-Home Transition<br>Nursing/Service: Person-centered, personalized, proactive, and continuous transition care service.<br>Consequences<br>The most used indicators were:<br>Outcome:<br>hospital readmission<br>mortality<br>emergency department visits<br>length of stay<br>total costs of the intervention<br>patient health-related quality of life<br>care burden<br>provider feedback<br>Process:<br>Adherence to the overall intervention protocol or individual intervention components.<br>Structure:<br>Health Technologies and Products<br>Electronic medical records were used to facilitate communication between healthcare providers and to inform the study coordinator about new patient admissions.<br>Various assistive technologies to facilitate TCM.<br>Assistive technologies promoted patients' ability to participate in follow-up care at home by	CASP 10/10	5

						providing a pedometer to record steps and distance in a diary and audio-recording patient teaching sessions so that patients and caregivers could review the tapes with provided recorders during the intervention.<br>An algorithm to create a list of evidence-based recommendations for each patient using a web-based database that covered all documented patient information.<br>Inclusion of the nurse practitioner's contact information in the hospital-wide text messaging system.<br>Other Considerations<br>Transitional care models should be multicomponent and include:<br>1. Pre-discharge and post-discharge follow-up components to reduce hospital readmissions and ED visits.<br>2. Components such as shared decision-making.<br>3. Patient and provider outcome measures.		
Liebzeit et al, 2021<br>USA	To examine the current scope of hospital-to-home transitional care interventions that impact health-related outcomes, as well as other key components, such as the engagement of older adults and their caregivers.	Scoping review of randomized controlled trials.	44 of 577 studies.	The most common components of transition interventions were:<br>Continuity and coordination of care<br>Medication management<br>Symptom recognition<br>Self-management<br>Few studies reported a focus on caregiver needs or goals.<br>Common intervention modalities included telephone, in-person care during the patient's hospitalization, and in-person care in the community after hospital discharge. The most common outcomes were readmission and mortality	To improve outcomes beyond healthcare use, a paradigm shift in the design and study of care transition interventions is required. Future interventions should explore novel methods or interventions for caregiver engagement; Promote an interdisciplinary team or care coordination center with the participation of underrepresented specialties, such as social work and occupational therapy; and examine opportunities for interventions specifically designed to address the needs of older adults and their caregivers, as well as their well-being.	Subject: patient, caregiver, healthcare team.<br>Goal: Address the needs of older adults and their caregivers, as well as their well-being, through actions that improve continuity, self-management, and timely response to early symptom alerts.<br>Context: Hospital, Home, Community<br>Nursing/Service: Telephone, in-person care during the patient's hospitalization, and care related to ensuring continuity, medication management, and in-person care in the community after hospital discharge.<br>Consequences:<br>There is a need to consolidate an interdisciplinary team and a care coordination center that works with patients and caregivers.	PRISMA 27/27	1

						Other Considerations:<br>Few studies reported a focus on caregiver needs or goals.		
Liebzeit et al., 2024<br>USA	To examine the process of caregivers' collaboration with others, including family members, healthcare professionals, and community, social, and professional networks, during older adults' hospital-to-home transitions.	This qualitative grounded theory	The study included 16 participants.	A model was developed that illustrates the participants' process through three stages:<br>1) identifying and learning the caregiver role.<br>2) collaborating with others to provide care and support to the older adult, while also supporting themselves, during the transition from hospital to home.<br>3) supporting the older adult's progress in regaining independence or planning for long-term care and support.	Participants described multiple approaches to collaborating with others:<br>Caring independently.<br><br>Caring within a network.<br><br>Caring with healthcare professionals.<br><br>Caring with social and professional networks.	Subject: patient, family members, healthcare professionals, community, social, and professional networks.<br>Goal: Strengthen autonomy<br>Context: Hospital-to-home transition.<br>Nursing/Service: 1) Identify and learn the caregiver's role, 2) Collaborate to provide care and support, 3) Support progress and recovery of independence or plan for long-term care and support.<br>. Consequences: Implications include the need to recognize and promote the use of care networks, as they can help address negative outcomes associated with caregiving. We also identified opportunities to further leverage caregivers' social and professional networks and pay greater attention to caregivers' needs during healthcare encounters.<br>Other considerations:<br>Unpaid/family caregivers provide crucial support during hospital-to-home transitions for older adults, but they lack the time and training.<br>Evidence on the importance of caregiver collaboration during the transition is limited.	CASP 8/10	6
Malley et al., 2015<br>USA	To identify nursing contributions to care transitions in the perioperative setting and the role of preoperative assessment in this transition.	Qualitative descriptive design with focus groups	24 nurses.	The nurses identified the following categories:<br>1) understanding patient vulnerabilities,<br>2) multidimensional communication,<br>3) managing patient expectations, and<br>4) nursing's role in compensating for deficiencies.	Nursing's relevant role in preoperative assessment during the transition of preoperative care is that of an advocate who identifies patient needs and risk factors that may be affected by surgical experience.	Subject: Patients, caregivers, nurse, healthcare team<br>Goal: Prepare the patient for their return home. Context: In-hospital transitions as preparation for a healthy transition home. The context is dynamic and complex. hospital and home.<br>Nursing/Service: Assess to identify vulnerabilities and warning signs; articulate information; Educate; advise;	CASP 10/10	6

						communicate; coordinate care; advocate; facilitate.<br>Consequences: Reduce the burden of care through communication and coordination.<br>Other considerations: This study suggests that preoperative nursing assessment can be useful in identifying and defining patient risk factors, not only for surgery but for the entire perioperative care trajectory.		
Markiewicz et.al, 2019<br>U.K.	Identifying key threats to the safety of patient transitions from hospital to primary care	Delphi method with three rounds	70 clinical and non-clinical staff from 39 primary care centers.	Threats include:<br>1) Age<br>2) Medical problems<br>3) Social factors<br>4) Medication.<br><br>Notable among these are:<br>1) Communication problems<br>2) Service organization<br>3) Medication supply<br>4) Caring for patients at higher risk<br>5) Poor quality of transfer instructions from secondary to primary care teams received the highest rating (mean rating in round three = 8.43) and a 100% consensus that this was a "very important" threat.<br>6) Older people (97%) and<br>7) patients with complex medical problems taking more than five medications (80%) were considered the most vulnerable.<br>8) Anticoagulants (77%) were considered to pose the greatest risk to patients.	This study identified specific threats to safe patient transitions from hospital to primary care, providing policymakers and healthcare professionals with targets for quality improvement strategies.	Subject: Patient, family, nursing, healthcare team<br>Goal: Reduce the threats to patients during their hospital-home transition.<br>Context: Hospital-home transition in a context that includes determining factors.<br>Nursing/Service: Assess, communicate, advocate, prevent, facilitate, coordinate, and develop policies.<br>Consequences: Identifying risks allows for alerts to be raised so that the transition can be safer.	MMA T<br>5/5	5
Miller et al., 2019<br>USA	To describe suggestions for improving care transitions for patients with stroke and their caregivers	Expert opinion based on the Transitions of Care (TOC) model from a group of experts	1 experts' group	.. Improving hospital-to-home transitions for people with stroke and their caregivers requires:<br>1) improving communication processes.<br>2) utilizing transition specialists.<br>3) implementing a patient-centered discharge checklist.<br>4) utilizing standardized outcome measures.	A comprehensive and standardized approach to implementing protocols for achieving a healthy transition during post-stroke rehabilitation is needed. Specifically, this article reviews current guidelines and provides a summary of the evidence for several commonly cited approaches (early supported	Subject: Stroke patient, caregiver, family, and community.<br>Goal: A comprehensive, patient-centered transition at discharge to maximize functioning and health.<br>Context: Hospital-home transition. Analyzed within a community setting.<br>Nursing/Service	JBI<br>6/6	7

				5) establishing collaborations with community wellness programs.	discharge, planned pre-discharge home visits, discharge checklists) for managing TOC, followed by a description of documented barriers to effective transitions. Standardized patient-centered transition management can improve community integration, performance in activities of daily living, and quality of life for stroke survivors, while reducing hospital readmission rates during the transition from hospital to home and community.	Consequences: To be able to identify strategies to reduce hospital stays and readmissions, as well as improve patient, caregiver, and healthcare professional satisfaction.<br>Other considerations: It is necessary to review current practice and evidence regarding transitions of care in stroke rehabilitation; identify gaps in transitional care and offer guidance for program development; provide relevant assessments and tools; Formulate recommendations based on a theoretical framework and evidence to improve the coordination of care for people with stroke from the hospital to the home and community.		
Mitchell et al.,2018<br>USA	To describe the experiences of patients and caregivers during care transitions and to characterize the outcomes desired by patients and their caregivers during care transitions	Qualitative descriptive focus group study	138 patients and 110 family caregivers recruited from six health networks in the United States.	Patients and caregivers identified three desired outcomes from care transition services: 1) feeling cared for and cared for by medical professionals; 2) having unequivocal accountability from the health system; and 3) feeling prepared and capable of implementing care plans. The professional behaviors that were linked to achieving these outcomes:<br>1) using empathetic language and gestures,<br>2) anticipating patient needs to support self-care at home,<br>3) collaborative discharge planning,<br>4) providing practical information,<br>5) providing uninterrupted care with minimal handoffs..	Clear accountability, continuity of care, and caring attitudes throughout the care continuum are important outcomes for patients and caregivers When these outcomes are achieved, care is perceived as excellent and reliable. Otherwise, the transition of care is experienced as transactional and unsafe, leaving patients and caregivers feeling abandoned by the healthcare system.	Subject: Patients and caregivers; healthcare staff<br>Goal: Improve functionality, community integration, and quality of life for patients and their caregivers.<br>Context: Hospital-home transition.<br>Nursing/Service: Provide warm treatment and appropriate communication; Anticipate patient needs to support self-care at home; collaborate on the discharge plan; inform and facilitate; ensure continuity of care; maintain an inclusive attitude.<br>Consequences: The patient-caregiver dyad feels cared for by medical professionals; supported by the system; and has adequate preparation and capacity to transfer care plans.<br>Other Considerations<br>Patient-Centered Checklist for Discharge to the Community:<br>1. Ask patients or caregivers about goals, resources,	CASP 10/10	6

						preferences, and concerns regarding the transfer of care.<br>2. Explain the discharge summary to patients or caregivers (use feedback if needed).<br>3. Train patients or caregivers on care (e.g., self-care or coping techniques, communication strategies, health prevention and maintenance, safe swallowing or dietary modifications, behavior management, and behavioral health issues).<br>4. Notify and provide the patient/caregiver, pharmacy, primary care physician, and home care agency with a discharge summary plan, medication reconciliation form, and hospital staff contact information.<br>5. Establish a health and social care plan agreed upon by all stakeholders, and the patient or caregiver knows who to contact in case of difficulties.<br>Follow-up<br>6. Confirm and identify the patient's primary care physician; alert the healthcare team if there is none or initiate a search.<br>7. Ensure follow-up by a member of the stroke rehabilitation team within 72 hours of discharge to verify the needs of the patient or caregiver.<br>8. Ensure periodic review by the healthcare team of patients to assess their recovery, prevent deterioration, maximize functional and behavioral health outcomes, and improve their quality of life (e.g., first week, 6 months, 12 months).<br>9. Reassess patients who experience a change or deterioration in their functional status, even months after the stroke. Medication Management and Health		

						10. Teach patients or caregivers how to properly use medications upon discharge and how they relate to their previous medications.<br>11. Assess the patient's ability to manage medications.<br>12. Prevent with interventions and resources for safe and accurate medication management (e.g., low vision, decreased dexterity, cognitive deficits).<br>13. Monitor risk factors for a second stroke (e.g., blood pressure, blood glucose, cholesterol, smoking cessation, stress, exercise, weight control, sedentary lifestyle).<br>• Home Readiness<br>14. Evaluate in patients with mild or moderate stroke, assess whether early discharge with support is feasible (availability of comprehensive multidisciplinary rehabilitation). 15. Assess patients and the need for a home visit (e.g., occupational therapy, physical therapy, nursing, family) to ensure functioning and safety in self-care activities.<br>16. Assess caregivers and the need for home modifications (e.g., grab bars, bathtub stool, ramps) and discuss plans/resources with patients or caregivers.<br>17. Ensure appropriate equipment (e.g., specialized seating, wheelchair) is available in the patient's home by the discharge date.<br>18. Provide referrals and resources for aids or equipment and support services to maximize recovery and facilitate participation (e.g., transportation, support groups, day programs).		
Monkong et al.<br>2020	To describe the evidence-based practice	This is a descriptive, exploratory	Nine healthcare professionals	Key components were considered:	The project demonstrated positive changes in transition care for	Subject: Patient, family caregivers, healthcare team	JBI<br>6/6	6

Canada	of transition care for older adults from hospital to home.	study with a multifaceted approach based on eight evidence-based clinical audit criteria for transition care, with follow-up to verify change.	were interviewed	Patient-centered discharge instructions; telephone follow-up; and preparation of family caregivers.<br>Improvements in clinical practice were identified regarding healthcare professionals' knowledge of transition care, patient care needs during the transition, and a multifaceted approach to service delivery.	older adults from hospital to home.<br>A multifaceted approach with patient-centered discharge instructions, telephone follow-up, and preparation of family caregivers is essential for older adults to successfully navigate the transition process from hospital to home.	Goal: Achieve a successful transition.<br>Context: Hospital-hospital transition.<br>Nursing/Service: A multifaceted approach with patient-centered discharge instructions, telephone follow-up, and preparation of family caregivers.<br>Consequences<br>Other considerations<br>Types of transitional care programs identified:<br>1. Home-based team approach<br>A coordinated approach for interdisciplinary health care teams working across the health care system that supports older adults to live in their own homes in the community for as long as possible. Requires a case manager. This approach seeks to reduce admission and readmission to acute care and expedite discharge. Services begin in the hospital and continue after discharge for a period. They often require intensive home care. Staff may be based in acute or community settings.<br>1. Patient navigation<br>Individuals provide support to patients, their family members, and caregiver friends as they access medical care and other services. Navigators can participate in assessment, provide information and referrals, and ensure appropriate services are provided in a timely manner. Navigators can be based in a hospital or community setting and focus on a particular population, such as older adults, Indigenous people, or those with a specific diagnosis.<br>2. Transitional Care Units<br>Intensive services and support provided in a healthcare facility,		

						such as a hospital or long-term care facility, for those who do not require hospital care but are not ready to return home to the community. An interdisciplinary healthcare team works to help patients regain independence and ensure appropriate community supports are provided prior to discharge. Services may include wound care, medication management, care plan development, physical therapy, and occupational therapy.<br><br>Facilitators and barriers to the hospital-home transition are:<br><br>1. The scope of the program.<br>A vision, mission, and program objectives are required to inform program design and the care model to best meet the needs of patient populations and community settings based on the existing resources available within a community.<br>2. Program structure.<br>This includes defining the human talent required to address transitions, defining their roles, and developing collaborative actions. This structure should include ongoing education and training, knowledge sharing among team members, and flexibility for healthcare providers to provide a wide range of services.<br>3. Continuity of care and individualized care.<br>The program must have the capacity to operate as a "hospital without walls," facilitating the meeting of individualized needs and continuity of care during discharge planning and the transition from one service to the next. Individualized care is key to		

						setting goals, providing education, enabling patients to become more independent, installing equipment, and then discharging patients from the program. Efforts must be made to adapt discharge plans within the community and connect patients with appropriate resources in the timeliest manner possible.<br>4. Funding.<br>Ensure sufficient and ongoing funding to address patients' often complex needs.<br>5. Health system infrastructure.<br>These programs must:		
Mora et al, 2017<br>USA	To compare transitional care model (TCM) interventions described in the literature and led by professional nurses (PNs) with standard care to reduce hospital readmission rates in older adults.	Literature review	: 8 studies including 11,085 patients	Interventions included post-discharge follow-up phone calls, home visits, and information transfer to the patient's primary care physician	Interventions led by advanced practice nurses decreased hospital readmission rates.	Subject: Patient, caregiver, nurse, healthcare provider.<br>Goal: Reduce hospital readmissions<br>Setting: Hospital-home transition<br>Nursing/Service: Care coordination, post-discharge follow-up phone calls, home visits, and information transfer to the patient's primary care physician.<br>Consequences: Interventions led by advanced practice nurses decreased hospital readmission rates.<br>Other considerations<br>More evidence is needed to confirm how advanced practice nurses impact the reduction of readmissions.	CASP 10/10	5
Morkisch et al 2020<br>Germany	To identify the components of the Transitional Care Model implemented with geriatric patients with multimorbidity to reduce readmissions	Systematic search 1994–2019 in English or German	3 articles.	Three articles met the inclusion criteria. One of the included trials applied the nine components of the Transitional Care Model (TCM) described by Hirschman and colleagues and achieved a high-intensity intervention level in the intensity assessment. This and another trial reported reductions in the readmission rate ($p < 0.05$), but the third trial reported no significant differences between groups in the longer	High-intensity multidisciplinary and multicomponent interventions are likely effective in reducing readmission rates in geriatric patients without increasing costs.	.Subject: Patient, caregiver, family caregiver, healthcare team<br>Goal: Ensure continuity of care, reduce hospital readmissions.<br>Setting: Transition: Hospital, home, community.<br>Nursing/Service according to the Transition Care Model consists of 9 components, as follows:<br>1 Assessment<br>2 Staffing<br>3 Maintaining Relationships	JBI 8/8	6

				follow-up period (up to 12 months).		4 Engaging Patients and Caregivers<br>5 Risk and Symptom Assessment/Management<br>6 Education/Promoting Self-Management<br>7 Collaboration<br>8 Promoting Continuity<br>9 Encouraging Coordination<br>Consequences<br><br>Other Considerations<br>The MTC model applied can reduce readmissions and costs.		
Morrison et al., 2016<br>USA	To describe the approaches and outcomes of two distinct transitional care programs serving different populations: one delivered by master's-level clinical nurse specialists (CNSs) focusing on chronic disease self-care; the other by palliative care physicians (PCPs)..	This was a retrospective comparative cohort study.	170 participants: 98 for the nursing program and 72 for the palliative care program	Patients in the CNS intervention had significantly fewer ED visits ($p < 0.005$) and hospitalizations ($p < 0.005$) in the 4 months following the intervention than in the 4 months prior to the intervention. Patients in the primary care program (PCP) had a nonsignificant reduction in ED visits ($p = 0.327$) and a significant reduction in hospitalizations after the intervention ($p = 0.03$).	Both transitional programs are useful in reducing rehospitalizations. The CNS intervention also significantly reduced emergency department visits in its target population. Transitional care programs have the potential to prevent unnecessary healthcare use during critical transition periods that leave patients vulnerable to adverse events and poor outcomes	Subject: Patient, Family, Healthcare Provider.<br>Goal: To reduce preventable adverse events and associated healthcare use (readmissions, emergency department visits, and costs) through timely follow-up after hospital discharge.<br>Context: Hospital-home transition<br>Nursing/Service: Post-hospitalization home visits with a focus on self-care or palliative care.<br>Consequences<br><br>Other considerations<br>Existing research has shown that transitional care programs with intensive follow-up reduce hospitalizations, emergency department visits, and costs.	MMAT 5/5	4
Murray et al., 2019<br>UK	To develop a dynamic and interactive model of patient engagement in care	A systematic literature search was conducted	16 of 3060 studies.	Patients and caregivers occupy and transition through multiple states of engagement in response to perceived interactions with healthcare professionals as they attempt to resolve health and well-being-related goals. "Non-engagement," "informative action," "challenge and pursue," and "autonomous action" were the main states of engagement.	The model suggests that engagement is highly challenging for older adults during care transitions. Moving forward, interventions seeking to support patient engagement should address dynamic states of engagement and their mediating factors. Studies indicate that patients desire to participate in their care. This review and	Subject: Patients, caregivers, health professionals.<br>Goal: Greater patient participation in care.<br>Context: Hospital-home transition<br>Nursing/Service: Maintain a good patient-professional relationship, dedicate adequate time, support access to the complex healthcare system (facilitate), prepare for return, listen, and assist.	CASP 10/10	5

				Feeling disengaged because of perceived exclusion leads patients to act autonomously, creating the potential for harm.	interaction model support this and show that nonparticipation is not a desired state for most patients, but rather a consequence of system-level forces and other contextual factors that act to erode efforts to participate.	Consequences: Greater closeness protects patients and caregivers from greater adverse risks. There is a need to understand how they engage in their own care.<br>Other considerations:<br>Future interventions require:<br>1. a more nuanced approach that helps staff recognize all states of patient participation as valid<br>2. reflection on how professional behaviors may influence participation<br>3. understanding how these may affect patient safety and experience.<br>4. For those who choose not to participate, a deeper understanding of the factors that perpetuate this status will be necessary.<br>Respect these individuals' wishes while countering the potential for widening health inequalities.		
Naylor et al., 2017<br>USA	To identify the components of transitional care (TC) that generate desired outcomes for patients and caregivers.	A multi-method study included results from previous studies and focus groups.	Literature review: 33 articles	Eight components of TC were identified:<br>1. Patient engagement.<br>2. Caregiver engagement.<br>3. Complexity/medication management.<br>4. Patient education.<br>5. Caregiver education.<br>6. Patient and caregiver well-being.<br>7. Continuity of care.<br>8. Accountability.	Health systems must address all eight components to ensure optimal TC for all beneficiaries.	.Subject: Patients, caregivers, healthcare personnel.<br>Goal: Improve patient health outcomes and reduce hospital readmissions.<br>Context: Hospital-home transition.<br>Nursing/Service: Assist, educate, facilitate, coordinate, prevent, seek continuity, promote self-care, and assume accountability.<br>Consequences: Transitional care can be improved when dyad participation is encouraged, and continuity of care is ensured.<br>Other considerations:<br>Little is known about the key elements of effective transitional care and how they relate to the needs and experiences of patients and caregivers.<br>The degree of attention paid to each component varies according to the specific needs of patients and caregivers.	MMAT<br>5/5	4

Naylor et al, 2018<br>USA	To describe and classify common local adaptations of the Transitional Care Model (TCM) and explore how and why it was adapted	A mixed-method study with sequential quantitative and qualitative phases was conducted	A survey was completed by 582 transitional care clinicians. 24 professionals were interviewed	96% reported an average of 4.4 adaptations to the 10 TCM components.<br>Nine out of ten respondents (94%) reported contextual adaptations, while content adaptations were reported less frequently (58%).<br>The first three reported context-related adaptations (i.e., providing services from hospital to home, using advanced practice nurses, and fostering continuity of care). There are several reasons for these modifications.	The findings reinforce the need to invest in the science of adaptation and suggest hypotheses to guide a rigorous analysis of the association between adaptations of TCM components and desired outcomes.<br>The three most frequently reported local adaptations of TCM components were: 1) providing services from the hospital to the home; 2) utilizing nurse practitioners (NPs) to implement services; and 3) promoting continuity of care (e.g., involving the same clinician in all settings).<br>According to the Stirman System, each of these modifications would be classified as a contextual adaptation.	Subject: Patient, caregiver, healthcare team<br>Goal: Improve the patient's experience with healthcare; improve clinical outcomes; facilitate processes in collaboration with community partners; reduce rehospitalization; reduce hospital stay; reduce costs<br>Context: Hospital-home transition in the context of return to the community.<br>Nursing/Service<br>Provide services from the hospital to the home; screen older adults at risk; Build trust in nurse practitioners (NPs); Promote continuity, coordinate care, collaborate with patients, caregivers, and staff, and maintain a good relationship with patients and caregivers.<br>Patient and caregiver engagement<br>Management of symptoms and other risks<br>Education and promotion of self-care<br>Consequences: Improve patient experience with care, health outcomes and quality of life, and total cost of care.<br>Other considerations: The following hypotheses are postulated from the study:<br>Adaptions to contextual components of MTC will have a greater influence on desired outcomes than content adaptations.<br>Organizations where staff perceive a high level of compatibility of MTC components with existing mission and objectives will perform better on desired outcomes.<br>The more closely the care program during transition is aligned with the evidence-based	MMAT 5/5	4

						MTC protocol, the greater the likelihood of achieving desired outcomes.<br>The longer organizations have been implementing MTC, the greater the likelihood of achieving desired outcomes.		
Nordmark et al 2016 Sweden	To explore the integration of the discharge planning process (DPP) from the perspective of registered nurses, district nurses, and home care organizers	The study design was exploratory, using standardized process theory (NPT) as a framework to explore DPP integration.	Analysis of discharge plans from five hospital areas. Comparison with results from three primary care centers and two municipalities.	Using NPT as a framework to explore the integration of discharge planning after 10 years of use showed that staff had reached consensus on the process (coherence) and how they evaluated it (reflective monitoring). However, there was no consensus on who carried it out (cognitive engagement) or how it was carried out (collective action). This could be interpreted as a lack of standardization of the process in the observed practice.	The result shows the need to observe the implementation of traditional practices to better understand the needs of new ones before developing and implementing new practices or support tools in healthcare to achieve the development objective and achieve sustainable implementation. The NPT offers a generalizable analytical framework that can explain and shape the implementation process of traditional practices before continuing to develop new practices or support tools.<br>Keywords: Discharge planning process, Implementation, Normalization Process Theory, Qualitative research.	Subject: Patient, nurse, home care organizers.<br>Goal: Ensure continuity of care during the transition, avoid delayed discharges, hospital readmissions, and inadequate post-discharge care.<br>Context: Hospital-home transition<br>Nursing/Service: Consistent service, reflective participation and monitoring, and collective action.<br>Consequences: Staff have consensus on the process (consistency) and how they evaluated it (reflective monitoring).<br>Lack of consensus on who conducts the planning process or how it is carried out (collective action).<br>It is important to understand traditional practices before developing and implementing new support tools.<br>Other Considerations<br>Discharge planning is a healthcare process that seeks to ensure the smooth transition of care for patients from home to hospital and back home.<br>Information sharing and collaboration among healthcare professionals are essential, but shortcomings are common.<br>Over the past three decades, various initiatives have been developed and implemented to improve the discharge planning process. However, high rates of medical errors and adverse events	MMAT 4/5	6

						related to failures in discharge planning remain.<br>The use of theoretical frameworks such as Normalization Process Theory (NPT) can support evaluations of complex interventions and processes in healthcare.		
O' Donell et al., 2021<br>Australia	To describe transition interventions that provide pre- and post-discharge support for older adults and evaluate their implementation and effectiveness in improving health and well-being.	A systematic literature review	20 studies	There are four types of interventions: educational, goal-oriented, exercise, and social support interventions.<br>Educational and goal-oriented interventions were effective in improving health and well-being outcomes.<br>The impact of the interventions on mitigating hospital readmissions was inconclusive.	Only five studies examined implementation. Older adults transitioning from hospital to home would benefit from personalized education and goal-oriented interventions that promote their self-care capacity.	Subject: Patient, nurse, healthcare team.<br>Goal: Promote self-care capacity, improve health and well-being.<br>Context: Transition from hospital to home<br>Nursing/Service: Educate, guide for therapeutic achievements, assist, and support.<br>Educational and goal-oriented interventions were effective in improving health and well-being outcomes.<br><br>Consequences<br>Interventions that support older adults' transition from hospital to home can address geriatric needs. However, this evidence base is fragmented and can affect well-being and quality of life outcomes, as well as impede continuity of care.	CASP 8/10	5
Paudel et al., 2022<br>Nepal (Asia)	To explore existing transitional care models used to improve the quality of care and outcomes of stroke survivors.	Integrated literature review	22 studies.	There are six main types of transitional care models focused on the transition of care for stroke survivors:<br>1) Naylor's Transitional Care Model (TCM),<br>2) Coleman's Transition of Care Interventions (TCI), 3) Reengineered Discharge Project (RED Project), 4) Better Outcomes by Optimizing a Safe Transition (BOOST Project), 5) Enhanced Discharge Planning Program, and 6) Comprehensive Post-Acute Stroke Services (COMPASS).	While all models differ in design, each can provide a framework for managing health conditions from the hospital to the home, in collaboration with the patient and their family. Therefore, transitional care models are effective in bridging the care gap between hospital and home care and improving the quality of medical care and outcomes for stroke survivors.	Subject: patient, family caregiver, healthcare professional.<br>Goal: Quality of care during the transition and avoiding complications and cost overruns.<br>Context: hospital-home transition<br>Nursing/Service<br>Consequences: The outcomes measured for the evaluation are: readmission rate; length of hospital stay; and net cost.<br>No studies have directly addressed process measures for evaluation, which are directly related to the quality of nursing care, such as patient outcomes: safety, satisfaction, and functional status.	CASP 7/10	5

						Other process-related outcomes, such as satisfaction with the care process, reduction of adverse events, and prevention of medical complications, are directly related to the quality of care.<br>Other considerations: Health systems use various transitional care models to improve the transition of care for patients. Six main types of transitional care models focused on the transition of care for stroke survivors are reported:<br>1) Naylor's Transitional Care Model (TCM),<br>2) Coleman's Transition of Care Interventions (TCI),<br>3) Reengineered Discharge Project (RED Project),<br>4) Better Outcomes by Optimizing a Safe Transition (Boost Project),<br>5) Enhanced Discharge Planning Program, and<br>6) Comprehensive Post-Acute Stroke Services (COMPASS).<br><br>Most of the study protocol content addresses patient self-management, as well as symptom management and medication reconciliation for patient safety. Very few studies have measured the outcomes of family caregivers; however, family caregivers participate equally in the care process and transitions with patients, and they experienced confusion and crisis in patient care.		
Pollack et al<br>2016<br>USA	To explore the challenges patients, face when transitioning from hospital care to self-management and the	A qualitative study using qualitative interviews and observations of patients during and after their hospitalization	28 patientes	We identified three key elements in this process: knowledge, resources, and self-efficacy. We describe how both system and individual factors contribute to failures that lead to ineffective patient management. This work expands our understanding of the unique challenges patients face	Despite their best efforts, hospital systems do not utilize their resources in ways that adequately support patients during the transition period.<br>Patients lack the key elements of knowledge, resources, and self-efficacy required for self-management outside the hospital.	Subject: patient, caregiver, healthcare provider.<br>Goal: Support patients and caregivers to achieve better health outcomes. The goal is to improve the opportunity to focus less on living with an illness and simply on living.	MMAT 5/5	5

	processes implemented to support and develop these skills.	s was conducted		during this difficult transition and uncovers important design opportunities to support patients' crucial, yet unmet, needs.<br>Patients discharged from the hospital are often unprepared for the transition.<br>Developing knowledge, resources, and self-management skills is necessary.	Finally, we describe design opportunities for new systems that support patients in their self-management process.	Context: Transition from hospital to home.<br>Nursing/Service: Mechanisms to strengthen their self-management and self-efficacy.<br>Consequences: Transition often exposes patients to unnecessary risks related to inadequate preparation before leaving the hospital, which can lead to errors and patient harm.<br><br>Other considerations: While patients who self-manage achieve better health outcomes and greater self-efficacy, little is known about the processes implemented to support and develop these skills in patients leaving the hospital.		
Rathert et al., 2015<br>USA	To empirically examine a conceptual model of patient-centered care using patient perception survey data.	Descriptive study with regression analysis.	Survey data from patients with overnight stays in142 hospitals.	Regression analysis found significant support for the theoretical model. Perceptions of emotional support showed the strongest relationship with overall ratings of care. Care coordination and physical comfort also showed a strong relationship.	Understanding how patients experience their care can help better understand what patients consider patient-centered and how care processes relate to important patient outcomes.	Subject: patient, caregiver, healthcare professional.<br>Goal: Providing patient-centered care.<br>Context: Hospital-home transition from hospital admission.<br>Nursing/Service: Facilitate transition, educate, inform, prevent, support network, therapeutic plan indications.<br>Consequences: A better understanding of patient-centered care from the patient's perspective will contribute to the advancement of research and practice.<br>Other considerations: Patient-centered care is one of the Institute of Medicine's goals for improving health care in the 21st century.<br>A better understanding of the relationships between dimensions of care and overall quality ratings can contribute to a greater understanding of how patient-centered care influences important outcomes. By developing a sound theory,	MMAT 5/5	4

						measurements can be improved and specific care outcomes can be better predicted.<br>In this way, practice can be improved because research will provide evidence that organizational leaders can use to justify changes for improvement.<br>List of indicators to measure patient-centered care:<br>1. Respect for the patient's preferences, values, and needs expressed.<br>2. Speaking in front of the patient, including them in the communication.<br>3. Respectful and dignified treatment<br>4. Coordination of care<br>5. Organization of the admission process<br>6. Waiting times<br>7. Consensuality of opinions among professionals<br>8. Scheduled tests and procedures performed on time<br>9. Information, education, and communication<br>10. Receiving explanations about unforeseen events<br>11. Receiving understandable explanations from healthcare personnel<br>12. Physical comfort level<br>13. Support in activities of daily living<br>14. Response time to doorbell<br>15. Timeliness in administering medications<br>16. Emotional support (being able to talk about anxieties and fears).<br>17. Feeling confident in professionals.<br>18. Being able to express concerns.<br>19. Involvement of family and friends.<br>20. The family had sufficient opportunity to talk with professionals.		

						21. Information was provided to the family about the condition or treatment.<br>22. Necessary information was given.<br>23. Continuity and transition.<br>a. The staff explained the purpose of the medications to be taken at home.<br>b. The staff explained possible side effects.<br>c. The staff explained the danger signs to watch for.<br>d. The staff explained when the patient could resume normal activities.<br>e. The patient knew who to call if they had questions after leaving.<br>24. Overall ratings of the quality of care.<br>a. Availability of health professionals.<br>care received from nurses, doctors, and the hospital.		
Rezapour-Nasrabad<br>2018<br>Iran	Describe the various aspects of the transitional care model, its benefits, and core components	Literature review:	5 of 80 articles from 2000–2017.	The transitional care model has several benefits, eight core elements, and seven core components, including: screening, staffing, maintaining relationships, assessing risks and symptoms, education, promoting continuity, and fostering coordination.	The transitional care model is a type of nursing-managed intervention to meet the care needs of patients with chronic illnesses during the hospital-to-home transition process.	Subject: The focus is on the patient, family, caregivers, physicians, and nurses.<br>The goal is to meet the care needs of patients with chronic illnesses during the hospital-to-home transition process.<br>Hospital-Home Transition Context<br>Nursing/Service managed care with an emphasis on continuity of care, prevention and avoidance of disease complications, and active patient and family participation in coordinated care with healthcare providers.<br>Consequences<br>- Fewer rehospitalizations and longer life expectancy<br>- Improved physical performance and quality of life<br>- Increased patient satisfaction<br>- Reduction in repeat hospital visits<br>- Reduction in healthcare costs	JBI<br>6/6	7

						Other Considerations<br>Model Components:<br>1. Screening: Patients with chronic illnesses who transition from hospital to home and are at high risk for poor outcomes.<br>2. Providing nursing staff with responsibility for care management during the initial stages.<br>3. Maintaining Relationships: Establishing and developing a trusting relationship with the patient and family.<br>4. Risk and Symptom Assessment/Management: Identifying and treating priority risk factors and symptoms and attempting to overcome them.<br>5. Education/Promotion of Self-Management: Preparing the patient and family to quickly identify and respond to worsening symptoms.<br>6. Promoting Continuity: Preventing disruptions in care from the hospital to home and ensuring continuity of care.<br>7. Promoting Coordination: Promoting connections between hospital-based healthcare facilities and the community.<br>Key Elements of the Transitional Care Model:<br>1. The transitional care nurse is the primary care coordinator to ensure the integrity of care delivery from the beginning of the program.<br>2. Regular visits to the patient by the nurse in their home or by telephone for at least 2 months after hospital discharge.<br>3. Home medical treatment continues through home visits by the transitional care nurse.<br>4. Focusing on the patient's holistic needs and addressing them based on evidence.		

						5. Involving the patient and family in care services, including education and supportive treatment.<br>6. Emphasis is placed on rapid disease identification and response to health risks and symptoms to achieve positive outcomes and prevent unwanted and negative events that lead to rehospitalization.<br>7. It is a team approach that includes the patient, family, caregivers, physicians, and nurses.<br>8. Establishing communication between the patient, family, and the healthcare team and other professional groups.		
Salehí et al., 2021<br>Canada	To model and analyze hospital-to-home transition processes in frail older adults to identify challenges in this process	A multiphase, multisite, mixed-methods design was used. Phase 1 included data collection from semi-structured interviews and focus groups, and Phase 2 consisted of six prospective case studies of patient-caregiver dyads	Six patient-caregiver case studies were used in the FRAM model testing process.	The results of building the FRAM model showed that five categories of functions contributed to the transition model: admission, assessment, synthesis, decision-making, and readmission. Results from using the customized version of the FRAM revealed challenges affecting the transition process, such as waiting lists for geriatric units, team-based care, lack of a discharge planner, financial concerns, and follow-up plans. The hospital-to-home transition process is a complex procedure that encompasses patient admission, assessment, treatment, discharge, and follow-up.	The process connects the hospital, home, and community to improve patient health and safety.<br>A wide range of perspectives, including healthcare professionals, patients, and caregivers, were engaged in the modeling phase to address the complexity of the process. The comprehensive model presented in this study allowed the research team to better understand the daily operations of caring for frail older adults during transitions. The model encompassed the admission process, patient assessment and treatment, the discharge process, follow-up plans, and readmission. The results of this study revealed how variability in outcomes for activities of daily living/functions created adversities for patients in the healthcare system.<br><br>Waitlist conditions, team-based care, discharge planners, Appendices. Appendix A Interview guide for patients and caregivers	Subject: Patient, Caregiver, Healthcare Professional<br>Goal: Identify and manage the strengths and weaknesses of transitions to improve patient health.<br>Context: The process links the hospital, home, and community to improve patient health and safety.<br>Nursing/Service: Activities related to the transition process (Guidelines/Policies/Standards; Referral and admission processes; Assessments/Evaluations/Monitoring; Goal achievement; Patient and family education; Interdisciplinary team collaboration and communication; Accessibility; Decisions; Plan of care; Interventions; Time; Use of technology; Coordination with municipal health services/discharge planning; Equipment/Service/Supply needs; Medication reconciliation and medication review; Patient communication and patient participation; Coordination with family members and primary care	MMAT 5/5	4

					Financial concerns and follow-up plans should be monitored and improved to address the adversities of the transition process. The results of this research will help healthcare professionals and decision-makers identify and manage the strengths and weaknesses of these transitions to improve patient health.	provider; Discharge planning and discharge criteria; Documentation and transfer/exchange of information; Resources; Problems/Variabilities; Outcomes; Follow-up care; Quality and safety; Other factors related to the transition process).<br>Consequences: The five categories of functions contributed to the transition model: admission, assessment, synthesis, decision-making, and readmission.<br>Other considerations<br>The findings of this study could help managers and other decision-makers improve transition processes for frail older adults by addressing these challenges. The FRAM method employed in this study can be widely applied to identify successful work practices, so that procedures and practices can be adapted to move healthcare processes toward paths that produce better outcomes.		
Saragosa et al., 2023<br>Canada	To further explain the complex social processes that people with dementia and their family caregivers undergo in response to care transitions.	Grounded theory	25 people. 4 people with dementia and 21 caregivers.	Duringand after the care transition, the caregiver is forced to take charge and connect the dots. While the caregiving experience is fraught with traumatic and very challenging situations, many caregivers manage to overcome them and reconcile their suffering with the desire to help their family member and others who may be going through similar experiences	During the care transition, the family caregiver is forced to take charge and connect the dots. Although the caregiving experience is fraught with traumatic and very challenging situations, many family caregivers overcome them. They can reconcile their suffering with the desire to help their family member and others who may be going through similar experiences.	Subject: Health Professionals, Patients, and Caregivers<br>Goal: To facilitate the health experience of patients with dementia and their caregivers during hospital-home transitions through quality services.<br>Context: Hospital-home transition.<br>Nursing/Service: Listening, empathy, support, support network, prevention.<br>Consequences: Evidence has shown that care transitions mark crucial changes in the lives of older adults, including significant and far-reaching changes for family caregivers.<br><br>Other Considerations<br>This study contributes theoretically to the literature on	MMA T 4/5	6

						care transitions by not only clarifying the visible work performed by people with dementia and their family caregivers during the process, but also by shedding light on the ongoing processes developed by family caregivers who interact within the health and social care systems with their family member with dementia.		
Solan et al., 2015<br>USA	To describe hospital-home transitions from the perspective of parents of hospitalized children.	Qualitative focus group study.	61 parents or caregivers of children discharged from the hospital. One focus group	87% of participants were women and 46% were non-White. 38% were the only adults in their household, and 56% resided in census tracts with ≥15% of residents living in poverty. Participant responses generated a conceptual model describing key elements of families' experiences with the hospital-to-home transition. Four main concepts were defined: 1) "In the fog" (obstacles to processing and acting on information),<br>2) "What I wish I had" (desired information and suggestions for improvement), 3) "Am I ready to go home?" (discharge readiness), 4) "I'm home, now what?" (confidence and post-discharge care).	The transition from hospital to home affects families' lives in ways that can impact patient outcomes after discharge Caregivers are key to a successful transition, and a family perspective can inform interventions that support families and facilitate a smoother return to home	Subject: Children, family members or caregivers, healthcare team.<br>Goal: Facilitate the hospital-home transition.<br>Context: Hospital-home transition<br>Nursing/Service: Inform, facilitate, guide, educate, anticipate, assist, accompany, support.<br>Implications: This model integrates our findings and clarifies the connections between experiences, beliefs, challenges, and needs. It incorporates the family's voice and can now be used to inform the design of interventions aimed at improving existing systems.<br><br>Other considerations: Hospital-to-home transitions can be difficult for patients and their families. Family characterization of this vulnerable period can facilitate the identification of interventions to improve transitions home. Our goal was to develop a comprehensive understanding of hospital-to-home transitions from a family perspective.	MMA T 5/5	6
Sun et al, 2023<br>Philippines	To support understanding of the factors that may influence health transitions in	A systematic search with a meta-synthesis and analysis based on Meleis's Transition	17 studies	The identified facilitators and inhibitors focused on the individual and community1. Resilience of older adults.<br>2. Relationships and connections.<br>3. Uninterrupted supply chain of care transfer.	The identified facilitating and inhibiting factors in transition for older adults can inform the development of interventions targeting resilience in adapting to a new family environment, human relationships and	Subject: Patient, caregiver, family, health professionals.<br>Target: Resilience in adapting to a new family environment. Foster human relationships and bonds to build partnerships, as well as an	CASP 8/10	5

	older adults from multiple perspectives, including older chronically ill patients, caregivers, and health professionals	Theory was conducted.			connections for developing partnerships, and an uninterrupted supply chain of care transfer in home birth.	uninterrupted supply chain for care transitions.<br>Context: Hospital-to-Home Transition<br>Nursing/Service: Comprehensive perspective, patient-centered care, interaction and coordination with the healthcare team, guidance, inspiration, motivation, and facilitation.<br>Consequences: Unhealthy care transition practices may be associated with a higher risk of adverse outcomes and readmission rates.<br>Other Considerations: Chronic illnesses are long-term, recurrent, and prolonged, requiring frequent travel to and from the hospital, the community, and home to access different levels of care.<br>The transition from hospital to home poses a challenge for older patients with chronic illnesses. The safety and quality of care transitions have received worldwide attention, and healthcare professionals have a responsibility to help older adults achieve a smooth, safe, and healthy transition.		
Takahashi et al.<br>2020<br>USA	Analyze common models for post-hospital transition and describe the Mayo Clinic model for care transition.	Analytical description of a hospital-home transition model.	One model	Providing transitions of care across different healthcare facilities is considered a high-cost and high-risk event, particularly for frail and high-risk older adults.<br>Some models utilize a nurse practitioner or nurse practitioner to reduce 30-day hospital readmissions.	The Mayo Clinic hospital-home transition model has demonstrated some success in providing care to high-risk adults in one US county.<br>Program interventions designed to improve outcomes as well as reduce costs have yielded mixed results and depend on how well the program meets the medical, functional, and psychosocial needs of its target population.	Subject: Patient, caregiver, healthcare team.<br>Goal: Better to meet patient needs.<br>Context: hospital-home transition.<br>Nursing/Service Educate, coordinate, assess, guide, facilitate.<br>Consequences Reduce hospital readmissions<br>Other considerations<br>The models are designed to lower healthcare costs by reducing 30-day hospital readmissions and improving chronic disease management.<br>Meta analysis have proven useful in identifying the most effective	6/6<br>JBI	6

						program elements designed to reduce 30-day hospital readmissions. A clustered, multidisciplinary approach best meets patient needs.<br>Successful care teams also emphasize self-empowerment for both patients and caregivers.<br>There are two general types of practice: Mary Naylor's, where an advanced practice provider cares for the patient for a set period, including home visits. Eric Coleman's, a transitions coach, who can be a registered nurse, a social worker, or a trained volunteer, acts as a healthcare coach while improving self-efficacy. Both models have been successful.<br>The Mayo Clinic's, with 7 years of experience, has been shown to reduce costs and improve patient satisfaction.		
Testa et al, 2020<br>Australia	Investigate care models designed to prevent or improve transitions of older adults from long-term care homes to hospital settings.	Integrative review	21 studies.	To prevent or improve the transition, the following are suggested:<br>1. Improved primary care in homes by implementing quality mechanisms.<br>2. Implementation of comprehensive hospital-based care.<br>3. Provision of outreach services with information transfer or a combination of outreach services and comprehensive hospital care.<br>As standalone interventions:<br>1. Standardized communication tools between services.<br>Standardized communication tools, as standalone interventions, can improve information transfer between nursing homes and hospital services.	For more complex care models, higher-quality evidence suggests that interventions that provide quality improvements and access to residential care facilities can prevent some types of hospital admissions if they incorporate the following into their design:<br>care coordination.<br>Access to qualified health professionals.<br>Capacity building and collaborations between services.	Subject: Patient, healthcare provider<br>Goal: Prevent or improve transitions of older adults from long-term care homes to hospital settings<br>Context: Nursing home-hospital transition<br>Nursing/Service: Educate, collaborate, facilitate, support, coordinate, assess, prevent, evaluate.<br>Consequences: Care transitions for older adults moving from residential aged care facilities (RACF) to hospital settings are associated with greater challenges and worse outcomes	CASP 8/10	5
Trasher, et al., 2018<br>USA	Effect of a multimodal discharge	Case report:	87 family caregivers of ventilator-	The simulation-based program was well received by participants.	Simulation training can be incorporated into post-discharge	Subject: patient, family caregiver, healthcare professional	JBI 6/6	6

	preparation curriculum incorporating simulation for family caregivers of children with complex medical conditions requiring long-term mechanical ventilation.		dependent children.	A trend toward a reduction in readmissions within 7 days of discharge has been observed since the implementation of the revised program.	training for families of children in transition.<br><br>Rehearsing emergency management in a simulated clinical setting increases caregivers' confidence in assuming care for their ventilator-dependent child.	Goal: Increase caregivers' confidence in assuming care during the transition to home.<br>Context: Hospital-home transition; Simulation context.<br>Nursing/Service: Educate, guide, facilitate, coordinate, anticipate.<br>Consequences: No statistically significant difference was observed in readmissions.<br><br>Other Considerations<br>Home medical emergencies are challenging. Opportunities for family caregivers to practice crisis management in a controlled environment before discharge are limited.		
Tutar et al., 2022<br>Turkey	To evaluate the psycholinguistic and psychometric properties of the Turkish version of the Transition Questionnaire, based on the Hospital-Home Transition Model.	This cross-sectional methodological study of linguistic validity, content validity, and construct validity.	380 parents in the neonatal intensive care unit	The content validity index was determined to be 0.89. The sample size was determined to be adequate for factor analysis (with a KMO value of 0.779 and a χ^2 (276) value in Bartlett's test of sphericity of 4026.181; $p < 0.05$). As a result of the exploratory factor analysis, 12 items were omitted from the questionnaire. The fit indices of the new three-factor structure of the questionnaire, formed after item deletion, were assessed using confirmatory factor analysis (χ^2/df = 4.783; RMSEA = 0.076; GFI = 0.776; AGFI = 0.726; CFI = 0.707; SRMR = 0.097). The reliability coefficient was found to range from 0.871 to 0.716 for the questionnaire subdimensions and 0.795 for the overall questionnaire.	The Turkish version of the Transition Questionnaire was determined to be valid and reliable for post-discharge assessments of parents with premature infants in Turkish society.	Subject: Newborn, parents, nurses, physician, friends.<br>Goal: To assess the hospital-to-home transition.<br>Context: Transition from the NICU to hospital to home. Includes websites.<br>Nursing/Service: Improve information, reduce anxiety, prevent problems, anticipate, coordinate,<br>Consequences: Models developed in the field of nursing play a crucial role in providing systematic, planned, and scientifically based care.<br>Other considerations: Parents face numerous problems related to home care for their infants in the post-discharge period and require professional nursing support. Professional support provided during this process should address all areas of care and be systematic and planned. Field-specific measurement tools are needed to accurately and systematically determine the needs of parents in home care for premature infants, who constitute a vulnerable population.	MMAT 575	4

Tyler et al., 2019<br>UK	To identify the evidence base for interventions that support continuity of care and safety in the transition from acute mental health hospitalization to community services at discharge.	Systematic review with narrative synthesis.	45 studies.	Some interventions followed a specific approach (e.g., Critical Moment Intervention, Transitional Discharge Model), others were grouped based on their key components (e.g., peer support, pharmacist involvement). The main problems the interventions sought to address were reducing readmissions, improving well-being, reducing homelessness, improving treatment adherence, accelerating hospital discharge, and reducing suicide. The 69 outcomes reported across the studies were heterogeneous, making it difficult to conduct comparative meta-analysis or quantitative synthesis.	The interventions reviewed span a spectrum from addressing a single problem within a single agency with a single solution to multiple solutions addressing multi-agency problems. We recommend that future research seek to improve consistency in reporting outcomes.	Subject: Patient, caregiver, nurse, other members of the healthcare team.<br>Goal: Reduce risks and anxiety to promote a successful transition.<br>Context: The transition from acute mental health hospitalization to community care<br>Nursing/Service: Information, support, assessment, care, psychoeducation, follow-up, promote adherence, anticipation, coordinate<br>Consequences: The main problems the interventions sought to address were reducing readmissions, improving well-being, reducing homelessness, improving treatment adherence, accelerating hospital discharge, and reducing suicide.<br>Other Considerations<br>Transition is assessed by:<br>Readmission<br>Length of stay<br>Mental health/psychopathology symptoms<br>Quality of life<br>Treatment adherence<br>Adherence to outpatient visits/appointments<br>Global functioning in the residence<br>User satisfaction with discharge<br>Medication adherence<br>Depression<br>Knowledge of their own illness<br>User satisfaction with treatment.<br><br>In this transition, individuals may experience additional risks and anxiety.<br>A better understanding of the elements of interventions that effectively reduce risk factors requires a more structured approach to evaluating interventions. This could be implemented through a generated	CASP 9/10	5

						standard; with greater clarity to explicitly state the problem (or latent risk factor) that an intervention aims to address (or each element of a multicomponent intervention). Most of the implementation research findings in the reviewed articles lack an underlying theory (more specifically, a theory of change) and an articulation of what is needed within a complex system for the intervention to be successful.<br><br>Conceptualizing these problems from a patient safety and systems thinking perspective, and with an explicit theory of change, can facilitate:<br>1) describing the specific problem that the interventions aim to address.<br>2) understanding the elements of an intervention that are effective in producing the desired medium- to long-term outcomes; and<br>3) understanding what long-term outcomes would indicate an effective intervention.		
Ulin et al., 2015 Sweden	To assess whether proactive care planning based on the Gothenburg person-centered care (gPCC) model improves the efficiency of discharge procedures compared to usual care in patients hospitalized for worsening	Experimental design with pre-post measurement	248 patients were included: 123 in the usual care group and 125 in the gPCC intervention group.	Notifications to the community home help service or 24-hour home nursing care services were more frequent in the gPCC group (33.8%) compared to patients in the usual care group (12.1%). The confirmed discharge planning meeting began within the first five days in the gPCC group, while in the usual care group, the duration ranged from one to 28 days. Compared with the usual care group, the gPCC group spent fewer days hospitalized (11 vs. 35) before discharge.	The gPCC improves discharge processes because patients are considered competent to participate in their care planning. gPCC involves creating a partnership between patients and healthcare professionals in the planning and delivery of care. In the hospital, this significantly improves the transfer process for patients with worsening CHF who require municipal home care services or 24-hour home nursing care services. The Gothenburg person-centered care model improves discharge processes because patients are considered competent individuals to	. Subject: Patients, caregivers, healthcare team.<br>Goal: Improve the efficiency of discharge procedures.<br>Context: Hospital-home transition.<br>Nursing/Service: Approaching patients and their caregivers as competent individuals improves the discharge process and invites them to participate in this planning.<br>Consequences: Discharge planning is important to facilitate the transition between hospital and home. Patients had fewer hospitalization days.<br>Other considerations	MMAT 5/5	3

	chronic heart failure.				participate in their care planning. This finding contributes to the growing evidence that the Gothenburg person-centered care model is more effective than usual care.	Many patients with chronic heart failure are often frail elderly patients with comorbidities and functional decline due to increased symptom burden.<br>A structured person-centered care approach in Gothenburg can promote better discharge planning.<br>Gothenburg's person-centered care model makes it easier for healthcare professionals to identify and utilize patient resources.<br>Gothenburg's person-centered care model improves the transfer process for patients with worsening chronic heart failure who require municipal care services.<br>Viewing patients as competent individuals improves the discharge process because they subsequently participate in planning their own care.		
Weeks et al 2021 Canada	To explore and describe the functioning of Canadian transitional care programs to support older adults and their family or friend caregivers.	Exploratory descriptive study	Nine key informants from different Canadian provinces	The main facilitators and barriers that influence the development and long-term success of transitional care programs include:<br>1. Program scope and structure.<br>2. Continuity of care.<br>3. Funding<br>4. Health system infrastructure.	Types of transitional care programs that support older adults with multiple health conditions in various settings are identified:<br>1. Those with a team-based home care approach.<br>2. Those with individualized patient guidance.<br>3. Transitional care units.	Subject: older adults and their family or friend caregivers, healthcare professionals, and an interdisciplinary team.<br>Goal: To meet patient needs and provide services using resources available in the community.<br>Context: Transition: Hospital/Home.<br>Nursing/Service leadership, coordination, establishment of a support network.<br>Consequences: Transitional care programs are crucial for improving health outcomes for some of the most vulnerable older adults and patients with chronic conditions, as well as for reducing the high-cost burden associated with unnecessary healthcare spending resulting from potentially avoidable use of the acute care system.<br>Other considerations:	MMAT 5/5	6

						Future research is needed to identify how a wide range of transitional care programs work and to disseminate knowledge among healthcare leaders and decision-makers to ensure that transitional care programs are integrated as essential services within the health system.		
Weis et al 2015 USA	To describe a model for conceptualizing the components of hospital discharge preparation to ensure attention to the full range of processes required for a comprehensive hospital discharge strategy.	Narrative description of the generated model.	One model.	The conceptual model for hospital discharge preparation merges the many existing components used in discharge and transition of care processes into a comprehensive framework for hospital discharge activities.<br>The compilation of components of transition of care models that begin in the inpatient care phase provides a menu of options for selecting a discharge process package for each component of the model.	The use of structured tools to support and evaluate the discharge preparation process will create a more standardized approach to measuring processes and outcomes.<br>This model should not replace well-tested discharge transition models and programs but should serve as a framework for operationalizing the discharge preparation process on nursing units and throughout the hospital.	Subject: patients, family, system<br>Goal: achieve adequate discharge preparation. Preventing readmissions and improving treatment adherence<br>Context: hospital-home transition.<br>Nursing/Service: plan, coordinate, educate, plan, collaborate, lead, maintain closeness, facilitate.<br>Consequences: The model allows for a more standardized approach to measuring processes and outcomes.<br>Other considerations:<br>Transitional care models focus on the coordination and continuity of care for high-risk populations who require some form of discharge preparation.<br>These include discharge preparation, discharge education, and discharge coordination.<br>These components need to be operationalized, along with their roles and interrelationships.<br>Outcomes of discharge preparation include healthcare professionals and encompassment dimensions such as physical and emotional state on the day of discharge, knowledge of personal and medical self-care, ability to function at home after discharge, and expected support in the post-discharge period.<br>Intermediate outcomes include successful management of patient and family care needs at home after discharge, with or without formal care services, or transfer to	6/6 JBI	6

						an appropriate post-acute care facility for continued care.<br>Coping difficulties or the development of post-discharge problems are a risk factor for poor performance.<br>Unplanned returns to the hospital for an emergency department visit or readmission should be avoided.<br>Adherence to the medical care plan, improved attendance at medical appointments, and fewer readmissions within 30 days should be achieved.		
Werner et al, 2019<br>USA	To identify and understand the performance determinants (PDFs) related to the success of the hospital-to-home transition in older adults.	A descriptive study with secondary analysis of interviews with recently discharged patients and their informal caregivers.	Interview data from 31 patients and 13 informal caregivers was used.	The four themes related to PDFs were:<br>1. The transition from hospital to home was a complex, multi-phased process: the process unfolded over several months and required substantial and ongoing investment and effort.<br>2. There were unmet needs for specialized tools: the information and resources provided upon discharge from hospital did not meet the requirements for a successful transition.<br>3. Alignment of self-care routines with transition needs is necessary: pre-hospitalization routines could be supportive or disruptive and could deteriorate or be reestablished.<br>4. Changes in work demand and capacity levels during the transition. Demand often exceeded capacity, resulting in work overload.	The findings highlight that the transition is not an episodic event, but a longitudinal process that extends beyond the days following hospital discharge. Transition interventions to improve the hospital-to-home transition for older adults must consider this complex multiphase process. Future interventions should be developed to support older adults and informal caregivers in establishing and reestablishing routines and managing work demands and capacity during the transition process.	Subject: patient, caregiver, healthcare team.<br>Goal: To reestablish routines after discharge during the phases of the hospital-to-home transition.<br>I frame the hospital-to-home transition not as an episodic event, but as a longitudinal process that extends beyond the days following hospital discharge.<br>Nursing/Service: educate, facilitate, coordinate, motivate, inform, guide, and accompany.<br>Consequences: Facilitating a successful transition from hospital to home for older adults remains a persistent challenge.<br><br>Other Considerations<br><br>The transition is multi-component and multi-phased.	MMAT 5/5	6
Wyer et al., 2016<br>USA & Canada	Implement a transition program based on evidence-based practice for people with heart	A three-phase protocol was developed. Readmission rates, adverse clinical outcomes, and	14 healthcare team members	The protocol emphasized patient education, medication reconciliation, and transition to community care. Senior management approved the hiring of a full-time HF nurse coordinator. 30-day HF readmissions decreased from	Training a multidisciplinary hospital team in the use of a knowledge transfer model, combined with ongoing facilitation, led to the implementation of a budget-neutral program that reduced HF readmissions.	Subject: HF patient, caregiver, healthcare team, educator.<br>Goal: To reduce 30-day readmissions of patients admitted to a community teaching hospital for heart failure (HF).<br>Context: Hospital-Home Transition	JBI 6/6	6

	failure. Hospital healthcare staff attended an innovative, three-day capacity-building conference in evidence-based care	program costs were tracked.		23.1% to 16.4% (adjusted OR = 0.64; 95% CI = 0.42–0.97) during the year following implementation. The corresponding rates at another hospital serving the same population but not part of the program were 22.3% and 20.2% (adjusted OR = 0.87; 95% CI = 0.71–1.08). Adherence to required HF quality measures improved. After an initial cost of $15,000, program expenses offset potential savings from decreased HF readmissions.		Nursing/Service: Training to develop evidence-based practice following a protocol.<br>Consequences: readmission rates, adverse clinical outcomes, and program costs. The training program returned to evidence-based practice, and improvements in the quality of health services were defined as educational outcomes.<br>Other Considerations<br>Evidence-based practice (EBP) training programs frequently fail to translate their content into practice changes or improved care.<br>We linked multidisciplinary EBP training to an initiative to reduce 30-day readmissions of patients admitted to a community teaching hospital for heart failure (HF).		
Xu et al, 2024 China	To explore the current scope of hospital-to-home transitional care programs for stroke survivors.	Scoping review.	49 studies.	This study identified transitional care programs for stroke survivors, as well as areas for future consideration and further exploration to help improve transitional care for stroke survivors in their transition from hospital to home.	This study demonstrates that multidisciplinary collaboration becomes an integral part of the transitional care model for stroke survivors, providing them with comprehensive and accurate medical care.	Subject: Patient, caregiver, healthcare team, community.<br>Goal: To improve the quality of care during the transition, reduce service use, and improve quality of life or well-being.<br>Hospital-to-home transitional setting for stroke survivors from before discharge to 12 months. Most studies provide 3-month follow-up with telephone calls.<br>Nursing/Service: Assistance, education, counseling, medication management, follow-up, visits, coordination, disease management, emotional support, community interaction, discharge assessment, home visit, telephone call, care and treatment plan records, functional exercise, home environment assessment, nutritional guidance, establishment of guidelines for healthcare team, rehabilitation training, discharge preparation,	CASP 10/10	5

						coaching, telephone and email follow-up, respect for culture, counseling.<br>Consequences<br>1. The study measures the use of health services:<br>Readmission<br>Length of stay<br>Hospital cost<br>2. Quality of care:<br>Satisfaction<br>Caregiving skills<br>Readiness for hospital discharge<br>Nursing quality<br>Caregiver psychological status<br>3. Health and well-being:<br>Quality of life<br>Psychological well-being<br>Activities of daily living<br>Physical functioning<br>Status Functional<br>Caregiver burden<br>Patient adherence<br>Incidence of complications<br>Mortality<br>Functional disorders<br>Health status<br>Cognitive functions<br>Blood pressure<br>Glucose levels<br>Lipid profile monitoring<br>Social regression (coping)<br>Uncertainty about the illness<br>Disability and falls.<br>To review the facilitators and inhibitors of transition, consider:<br>1. In the implementation process:<br>The multidisciplinary team<br>2. In the external environment<br>Interpersonal support<br>3. In the characteristics<br>Financial support<br>4. In the subjects<br>Trust<br>5. In the internal environment<br>Good communication.<br>The transitional care model has been shown to be effective in reducing readmissions and			

						mortality, thereby improving health outcomes and patient satisfaction among stroke survivors.<br>Other considerations<br>Stroke survivors face the dilemma of resolving numerous complex issues that put them at high risk of readmission upon discharge from the hospital.		
Ye et al., 2016 China	To review the literature on the development of TCM in China, its current status, problems, and strategies related to its implementation, and to suggest strategies related to the future of TCM in China.	Systematic review:	26 articles.	There is a paucity of updated information<br>on the development and implementation of TCM in mainland China. However, the spectacular growth of transitional care in the last 5 years has had a vital impact on society and the development of nursing.	TCM has the potential to achieve a successful and beneficial impact in China. Evidence is lacking on education and training, as well as policies for nurses in this context.	Subject: Patient, family, caregiver, nurse, healthcare team.<br>Goal: To support caregivers in their needs, meet the demand for nursing services and their continuity, improve activities of daily living, maintain or improve quality of life, improve adherence, meet services required by patients, psychological well-being, improve judgment, improve clinical outcomes, reduce risk factors, improve self-care capacity, improve satisfaction, maintain or improve functionality.<br>Hospital-Home Transition Context.<br>Nursing/Service: Telephone and home follow-up, problem-solving, education, care planning, care coordination, comprehensive instruction, counseling, empowerment for self-management, prevention of complications, anticipation of required support, evaluation of progress, clear communication, establishment or assistance in achieving a support network, record-keeping, support, promotion of healthy habits, counseling, and guidance in health care processes.<br>Consequences: There is a lack of evidence on education and training, as well as policies for	CASP 10/10	5

						nurses in the field of transitional care between hospital and home.<br>Other Considerations<br>Challenges<br>1. Development of robust guidelines on the efficient implementation of TCM.<br>2. Related policies that can manage emergency conditions during the follow-up process and ensure the safety of nursing staff.<br>3. Improve communication between the community and hospitals.<br>4. Availability of funding for the operation is also important.<br>5. Joint efforts of physicians, nurses, social workers, and other professionals.<br>6. Evaluation and implementation of multidimensional interventions.<br>Strategies<br>Develop policies that support the interests of participants in post-discharge processes.<br>Create a standard training program and evaluation of staff, such as nurse practitioners, dietitians, physical therapists, and psychologists.<br>Require certification for those who manage MTC.<br>Standardize performance evaluation in this field, with compensation for nurses who receive favorable feedback from patients.<br>Coordination between the hospital and the referral community.<br>Home visits include patients and caregivers.		
Zupanc et al, 2020<br>USA	Provide practical information on developing a transition	Critical reflection on the topic.	N.A:	The following variables should be considered in the transition of care from adolescent to adult with neurological disorders:	There are numerous obstacles to implementing existing transition guidelines:	Subject: Adolescents in the process of transitioning to adulthood, age 13 or older, with a neurological disorder, their parents, and healthcare team.	JBI<br>6/6	7

	program, as well as a possible clinical model for transition of care.			1. Complexity of the young person's neurological condition.<br>2. Comorbidities of the young person's neurological condition, including behavioral problems, autism, sleep disorders, intellectual disabilities, and eating disorders.<br>3. Availability of subspecialty services.<br>Location and availability of adult subspecialists: rural vs. urban areas.<br>1. Insurance coverage.<br>The following are barriers to a successful transition within the family:<br>1. Separation anxiety between the patient and family.<br>2. Differences between the pediatric model of care and the adult model of care, with a more family-centered, multidisciplinary, and "medical home" approach in the pediatric setting.<br>3. Adult neurologists have fewer resources than pediatric neurologists, such as social workers, case managers, and psychologists.	1. Adequate number of trained and willing healthcare professionals<br>2. Patient and family anxiety about the transfer<br>3. Changes in health insurance<br>4. Inadequate reimbursement<br>5. Inefficient communication systems to facilitate a smooth transition<br>Three key measures of success have been identified:<br>1. A joint clinical visit between the pediatric neurologist, the adult care provider/subspecialist, and the patient's current primary care provider(s) (usually the parents).<br>2. A written transition plan; and<br>3. A patient care coordinator.<br><br>Possible additional metrics that could be used to measure a successful transition include:<br>1. Appointment completion rate with the adult care provider.<br>2. Patient and family satisfaction;<br>3. Stable or improved neurological condition; 4. Rate of unscheduled emergency room visits and/or hospitalizations during the first 1 to 2 years after transitioning to adult care.<br>5. Recovery rate with a pediatric neurologist	Goal: Ensure a successful transition, reduce risks, costs, and unnecessary service utilization.<br>Context: Hospital-Home Transition<br>Nursing/Service: Accompany, educate, guide, facilitate, anticipate, coordinate, monitor, assess, adjust, and provide reassurance.<br>Consequences: Without a structure that addresses the transition of medical care, adolescents and young adults with neurological disorders are likely to experience disruptions in their care, resulting in a greater need for emergency care and hospitalization.<br>Other Considerations<br>A systematic and consistent review of the transition process is necessary to determine the effectiveness of adolescent transitions to adult care.<br><br>Additional helpful tips for a successful transition:<br>1. We provide our personal cell phone numbers to our adult colleagues, so they do not feel alone in managing the patient. This is especially important in the early stages of the transition.<br>2. We also provide educational materials about the patient's diagnoses and medications, especially if the diagnosis is relatively rare, the adult care provider is unfamiliar with the diagnosis, or the prescribed medications are not commonly used in adult medical practice.<br>3. We also ask our families to contact us after the initial visit with the adult care provider to tell us how it went, what information they think the provider needs, and other details.		

						4. We also use this phone call to obtain feedback from the patient and family about their satisfaction with the new provider.<br>5. Sometimes the fitness is not perfect, and adjustments need to be made.<br>6. We fully trust our case manager to make these phone calls.<br>7. We usually know when the appointment is scheduled, so if the family doesn't contact us, we contact them.<br><br>There are significant barriers to transition, but they are not insurmountable. Educating each patient/family about potential barriers to transition is critical to success. The transition should be gradual, with a transition plan developed by age 14, adjusting it over the years leading up to the transition.<br>A case manager, preferably a nurse or nurse practitioner, is very important, probably essential to managing a successful transition of care.<br>Establish and maintain a list of adult health care professionals willing to accept adolescents/young adults with special needs into their practice.<br>Education for adult health care professionals is critical to a successful transition, with continuing medical education credits provided at the local, regional, and national levels. Our national professional organizations should lead this effort and emphasize its importance.<br>Education on reimbursement strategies and CPT codes is equally important for success.		

Zwart et al 2021 Nether--lands USA	To deepen the understanding of the functioning of care transition interventions between hospital and home by analyzing the contextual elements and mechanisms that may have influenced the success of these interventions, and by developing a conceptual model of how these components relate to each other.	A qualitative study using semi-structured face-to-face interviews based on realist evaluation methods.	26 participants.	Three main contextual factors:<br>1. Internal environment.<br>2. External environment.<br>3. Patient population.<br><br>Seven working mechanisms: simplification, verification, connection,<br>translation,<br>coaching,<br>monitoring and anticipation were found to be relevant to the outcome of care transition interventions.<br><br>Context, Intervention, Mechanism, and Outcome (CIMO) configurations revealed that, in response to these contextual factors, care transition interventions activated one or more of the mechanisms, in turn generating outcomes, including a safer transition of care.	We developed a conceptual model that explains how care transition interventions work in different contexts and believe it can contribute to the successful future implementation of care transition interventions.<br>Keywords: care transition interventions, context, mechanisms, quality of care, conceptual model.	Subject: patient, caregiver, healthcare team<br>Goal: To provide a safer care transition.<br>Context: Hospital-home transition<br>Nursing/Service: Simplification, verification,<br>connection, translation, coaching, monitoring, and anticipation<br>Consequences: Failure to implement safe care transitions after hospital discharge leads to unnecessary worsening of symptoms, a prolonged period of illness, or hospital readmission.	MMA T 5/5	6

Source: Own elaboration, 2026.
