# Supplementary material for: Adaptation Model for Patient and Caregiver Dyads in Hospital-to-Home Transition: Theory Development and Content Validation
Source: Healthcare (Basel). 2026 Sep 8;14(18):2905. doi: 10.3390/healthcare14182905 (PMC13606408; doi:10.3390/healthcare14182905)
Supplement: Supplementary file 1 [file healthcare-14-02905-s001.zip › Supplementary material S5 18 09 2026.PDF]

Supplementary Material S5. Experts' profile

Judge No.	Gender	Education	Current Role	Years of Experience	Country
1	Female	Bachelor's degree in nursing, Specialist in Nephrology and Dialysis Nursing, master's degree in social Gerontology, PhD Candidate in Health Sciences	Director of Postgraduate and Continuing Education Programs	27	Chile
2	Female	Nurse, Cardiorespiratory Nursing Specialist, master's degree in nursing with an emphasis on cardiovascular health care, PhD in Nursing.	Full Professor	35	Colombia
3	Female	Nurse, master's in nursing	Subdirector of Nursing in a Hospital Network	32	Colombia
4	Male	Nurse, master's degree in Basic Biomedical Sciences, Doctoral Student in Nursing	Nursing Coordinator at a University Hospital	33	Colombia
5	Female	Nurse, Doctor in Nursing	Director of the Nursing School	32	Mexico
6	Female	Nurse, Specialist in Cardio-Respiratory Care, Doctor of Nursing	Director of Postgraduate Studies in Nursing	43	Colombia
7	Female	Nurse, Critical Care Specialist, Public Health Specialist. Master's degree in health administration. Master's degree in organizational leadership.	Subdirector of Nursing at a University Hospital.	25	Colombia
8	Female	Nurse, Specialist in adult critical care, Doctor of Nursing.	Director of the undergraduate nursing program.	29	Chile
9	Female	Pharmaceutical chemistry. Doctor of Natural Sciences.	Director of the Department of Pharmacy and the therapeutic evidence research group.	25	Colombia
10	Male	Systems Engineer, master's in computer science, Doctor in Applied Sciences	Associate Professor	30	Colombia
11	Female	Nurse, Doctor of Nursing.	Full professor and researcher	30	Peru

Source: Own elaboration, 2026.
