# Supplementary material for: Adaptation Model for Patient and Caregiver Dyads in Hospital-to-Home Transition: Theory Development and Content Validation
Source: Healthcare (Basel). 2026 Sep 8;14(18):2905. doi: 10.3390/healthcare14182905 (PMC13606408; doi:10.3390/healthcare14182905)
Supplement: Supplementary file 1 [file healthcare-14-02905-s001.zip › Supplementary material S6 18 09 2026.pdf]

Supplementary Material S6. Expert Feedback During the *Adaptarte Model* Content Validation.

Category	Experts' Observations	How researchers address the observation
1. Conceptual and Epistemological Foundation	Explain the type of model and its classification within the knowledge hierarchy.	It is clarified that this is a nursing model classified within the knowledge hierarchy as a middle-range theory.
	Make nursing philosophy explicit and its contribution to interdisciplinary work.	The nursing philosophy underlying the model and its contribution to interdisciplinary work were included.
	It could later be complemented with other related theories or models.	The analysis is complemented with the theories of Roy and Meleis, and a follow-up plan for the model's development will be maintained.
	Review coherence between philosophy and concepts (possible concept analysis).	Reviewed. This analysis was included in the previous work, which clarified the concept of adaptation of the patient and family caregiver during the H–H transition.
	Include reference theories: Roy, Meleis, and social innovation.	These theories are included as part of the analysis.
	Highlight the originality of the model.	The article explicitly states that this is an original work.
2. Terminological and Semantic Clarity	For the title, consider a subtitle to indicate its application to nursing care in the H–H transition.	It is explicitly stated that this is a nursing model.
	In the definition of concepts, it would be useful to specify adaptation indicators for the transition, e.g., coping, adherence to treatment, risks, etc.	Adaptation indicators for the transition are included in later developments such as the *Adaptarte* tool.

	Some assumptions require refinement. For example:<br>• The first assumption could be interpreted as a total delegation of care to the caregiver, overlooking the nursing and health system's responsibility. Suggested wording: "The patient and their family caregiver are key agents in their health care, in shared responsibility with the nursing team and the health system."<br>• The second assumption may not always hold, as the patient might resist or refuse adaptation—variability of responses in the transition process should be considered.<br>• The third assumption is too general and does not make the nurse's facilitating role explicit.<br>• The fifth assumption is not explicit regarding the changing context—it could be improved by specifying, for example, family environment, health condition, etc.	The assumptions are reviewed and refined where appropriate; however, they are confirmed as valid.
	Logical propositions could be organized by dimensions, e.g., patient–caregiver; nursing role; outcomes–experience.	Propositions were reviewed and restructured.
	In formulating hypotheses, considering that the model may require future validation might be useful to classify hypotheses as primary and secondary.	The hypotheses were reviewed, but classification was deemed unnecessary.
	Replace *patient* with *person* or *care subject*.	The dyad composed of the *patient* and *family caregiver* is described.
	Replace *face* with *cope with*in the assumptions.	The verb *cope* was used in the assumptions.
	Include a glossary of key terms, especially *safe transition*.	The concept's attributes are described in its definition, and the operational definition of *safe transition* is included.
3. Scope and Context	Explain intrahospital transitions as well.	Intrahospital transitions are explicitly included.

	Add a summary of the Latin American context and challenges.	A summary of the Latin American context and challenges was incorporated.
	Acknowledge as a limitation the current applicability to other Latin American contexts.	The limitation regarding applicability to other contexts is acknowledged.
	Expand on barriers to adaptation in transition: resources, policies, resistance.	Barriers to adaptation were reviewed and complemented.
	Explicitly highlight the importance of context and institutional support.	The importance of context and institutional support is explicitly stated.
	Include suggestions for public policies and regulatory frameworks.	Suggestions for public policies and regulatory frameworks were included.
	In the future, it could be expanded to other transition scenarios such as palliative care, rehabilitation, etc.	A projection of the model's applicability to different fields was established.
4. Limitations and Projection	Address limitations in the literature regarding methodology, structure, and context.	Limitations of the literature in methodology, structure, and context were included.
	Identify biases or gaps in transferring the model to other contexts and propose strategies (human resource training, progressive adoption, teamwork, follow-up meetings, inter-institutional validation).	Biases and gaps in transferring the model to other contexts were identified, and strategies were proposed (human resource training, progressive adoption, teamwork, follow-up meetings, inter-institutional validation).
	Define longitudinal projection strategies.	Longitudinal projection strategies were specified.
	Adaptation of the model to different contexts.	A projection of the model's applicability to various contexts was established.
	State current and forthcoming developments (tools, interventions in different contexts).	Current and forthcoming developments (tools, interventions in different contexts) were described.
5. Design and Applicability	Illustrative diagram.<br>Suggestion: as it is a process, it should reflect input elements – transition process – and outputs (results). In other words: environment – process – impact.	The diagram was reviewed and revised to depict a process rather than a cycle, making the central elements of the H–H transition and its outcomes (effect and impact) evident.
	Present the diagram in a flow format rather than a cycle.	The diagram was adjusted to a flow format.

	Define the model's structure, processes, and products, including outcome metrics and cost–benefit analysis.	The relevance of the model's structure, processes, and products was clarified, along with outcome metrics and cost–benefit analysis, to guide future development.
	Develop an implementation guide with phases, roles, indicators, expected standards, and training for implementation leaders.	A commitment was made to develop an implementation guide with phases, roles, indicators, expected standards, and leadership training to advance the model's development.

Source: Own elaboration, 2026.
